# Postoperative complications of anterior cervical discectomy and fusion: A comprehensive systematic review and meta-analysis

**DOI:** 10.1016/j.xnsj.2025.100596

**Published:** 2025-02-08

**Authors:** Roozbeh Tavanaei, Ali Ansari, Amirali Hatami, Mohammad Javad Heidari, Mohammadreza Dehghani, Ahmad Hajiloo, MirHojjat Khorasanizadeh, Konstantinos Margetis

**Affiliations:** a Functional Neurosurgery Research Center, Shohada Tajrish Comprehensive Neurosurgical Center of Excellence, Shahid Beheshti University of Medical Sciences, Tehran, Iran; b Student Research Committee, Shiraz University of Medical Sciences, Shiraz, Iran; c Health Policy Research Center, Institute of Health, Shiraz University of Medical Sciences, Shiraz, Iran; d Student Research Committee, Lorestan University of Medical Sciences, Khorramabad, Iran; e Student Research Committee, Fasa University of Medical Sciences, Fasa, Iran; f Department of Neurosurgery, Mount Sinai Hospital, Icahn School of Medicine, New York City, NY, United States

**Keywords:** Anterior cervical discectomy and fusion, Meta-analysis, Cervical spine, Spinal surgery, Spine, Fusion, Complication, Safety

## Abstract

**Background:**

Anterior cervical discectomy and fusion (ACDF) is one of the most frequently performed spine procedures for different indications in the cervical spine. Various postoperative complications have been reported following the ACDF. This systematic review and meta-analysis aimed to calculate the incidence rate of different postoperative complications associated with ACDF surgery and also identify underlying risk factors for each complication.

**Methods:**

A systematic review of the literature was performed in PubMed/MEDLINE, Embase, and the Cochrane Library for observational studies published between January 1996 and March 2023 and reporting postoperative complications associated with ACDF. Randomized controlled trials and interventional investigations were not included in this study. Meta-regression was also performed using generalized linear mixed models with a binomial probability distribution on various potential predicting factors.

**Results:**

A total of 222 studies reporting the rate of complications associated with ACDF in 50,584 patients were included in the present study. The overall postoperative complication rate was 16%. The most common complications were excessive neck swelling (11.3%), pseudarthrosis (10.0%), dysphagia (9.5%), cage/graft subsidence (9.4%), worsening myelopathy (7.7%), and hoarseness (2.3%). The rate of nonhome discharge, readmission, and mortality were 13.8%, 3.7%, and 0.1% respectively. Based on meta-regression, more levels of fusion and increased age were significantly associated with an increase in the pooled overall postoperative complication rate. Moreover, the rate of some postoperative complications was significantly associated with a number of perioperative characteristics.

**Conclusions:**

To our knowledge, this study has been the most extensive meta-analysis conducted on the existing literature regarding ACDF-related complications and potential risk factors. However, future high-quality prospective studies or clinical trials are highly required to provide further evidence and also validate the present findings.

## Introduction

The anterior cervical discectomy and fusion (ACDF) is one of the most commonly performed spine procedures for the treatment of a variety of indications in the cervical spine [1]. This procedure is generally used to treat nerve root or spinal cord compression by decompressing these structures in the cervical spine, which is followed by vertebral stabilization. Given its effectiveness and safety profile, ACDF has been regarded as the gold standard for the treatment of various degenerative cervical spine pathologies since its introduction in 1958 [2]. Prior reports have demonstrated a substantial increase in the overall annual number of spine procedures performed for degenerative indications, such as ACDF over the last decade, which is due mainly to population aging [3,4]. Therefore, clinical optimization in ACDF surgery is of paramount importance to both improve patient care and reduce healthcare costs [[5], [6], [7]].

Despite its well-established effectiveness, ACDF is associated with a number of postoperative complications, such as dysphagia, hoarseness, instrumentation failure, infection, or neurovascular injury [8,9]. Prior observational studies have shown different rates for overall postoperative complications in ACDF, ranging from 13% to 19% [8,[10], [11], [12]]. Moreover, several reports have demonstrated the incidence of different ACDF-related postoperative complications specifically [9]. Nevertheless, to our knowledge, no meta-analysis has been devoted to postoperative complication rates of ACDF and potential associated risk factors thus far. In addition, due to differences in patient characteristics, surgical techniques, and current guidelines, no consensus exists on the best strategies for complication management in ACDF. Further, identifying potential risk factors could significantly prevent postoperative complications and help in developing effective guidelines for their management.

Therefore, the present systematic review and meta-analysis were designed to determine the complication rates after the ACDF surgery as well as potential underlying risk factors for each complication based on the available literature.

## Material and methods

The present systematic review and meta-analysis were performed according to guidelines provided in the Cochrane Handbook for Systematic Reviews. Additionally, to improve the reporting quality, the Preferred Reporting Items for Systematic Reviews and Meta-Analyses (PRISMA) guidelines were followed in the present study.

### Search strategy

A systematic search and literature review were conducted in PubMed/MEDLINE, Embase, and the Cochrane Library using search terms selected based on PICOT (population, intervention, comparison, outcome, time) format. Search terms were found through literature review and using MeSH as well as Excerpta Medica Tree (EMTree) terms. Different combinations of the following search terms were used in this study: “cervical spine surgery,” “ACDF,” “anterior cervical discectomy,” “anterior cervical discectomy and fusion,” “complication,” “complications,” “safety,” “risk factor,” “risk factors,” “dysphagia,” “hematoma,” “recurrent laryngeal nerve palsy,” “Horner's syndrome,” “Dural perforation,” “infection,” “dehiscence,” “pneumonia,” “subsidence,” “hoarseness,” “axial pain,” “C5,” “nonunion,” “cerebrospinal fluid leak,” “pharyngeal perforation,” “esophageal perforation,” “feeding tube,” “thromboembolism,” “respiratory,” “pulmonary,” “myelopathy,” “radiculopathy,” “neck swelling,” “pseudarthrosis,” “cardiac,” “myocardial infarction,” and “mortality.”

All peer-reviewed articles published between January 1996 and March 2024 that reported rates of postoperative complications associated with ACDF were evaluated for eligibility. Merely observational studies, either retrospective or prospective, written in English were included in this study, and animal studies, editorials, case reports, case series with fewer than ten patients, clinical trials or interventional investigations, national surveys or reports from national registry programs, and review studies without quantitative results were excluded. Clinical trials or interventional studies were excluded since they only include a specific sample and do not represent the general population of patients undergoing ACDF, given the use of inclusion/exclusion criteria based on their design.

Furthermore, previous findings have demonstrated that the inclusion of both trials and observational studies in meta-analyses could affect the reliability of results [13,14]. National surveys or results from registries were also excluded mostly due to the potential risk of including duplicates and increasing the heterogeneity. Reference lists of systematic reviews and meta-analyses were manually checked for potentially eligible results. Studies that only included populations with a specific condition potentially affecting the complication rates, such as age group (>65 years), nondegenerative primary indication (e.g., tumor, trauma, or infection), or comorbidities (e.g., osteoporosis) were also excluded. Further, any study that included a population of patients with a specific complication in all of them (e.g., dysphagia), was excluded. In addition, studies with heterogeneous populations in terms of anterior surgical approach (e.g., anterior cervical corpectomy and fusion [ACCF]) with no subgroup analysis on the ACDF group were excluded. If a group of authors published multiple reports of the same cohort of patients, merely their most recent publication with the largest sample size was included in this study. Based on the study criteria, 2 reviewers screened each article independently. The screening was first performed at the title and abstract level and then at the full-text level if the article was deemed relevant.

### Data extraction

Five authors (AA, AH, AH, MJH, MD) extracted the data, and 2 authors (RT, MK) performed the data review independently. The full text of all articles was thoroughly reviewed and the following data were extracted: study ID, study country, publication year, DOI or PMID, first author, study design, recruitment period, follow-up duration, loss to follow-up (percentage), mean age, sex (percentage), comorbidities reported, surgical indication, fused levels, number of levels of fusion, implant type, ACDF technique, overall sample size, mean length of stay (LOS), postoperative complication rates, nonhome discharge rate, readmission rate, and mortality rate. Implant types included structural allograft, titanium cage, and PEEK cage. The ACDF technique was defined as one of the following 3 techniques: use of cage without plating, use of the plating system with cage/graft use, or use of integrated or standalone cages for ACDF.

The criteria developed by the Oxford Centre for Evidence-Based Medicine and also modified by Wright et al. were used for methodological quality assessment in the present study [15,16]. According to this criteria, prospective cohort studies, retrospective cohort studies, and case series are graded as I, II, and III, respectively. The level of evidence (LoE) of studies was downgraded by 1 grade in case of loss to follow-up of more than 20%. Moreover, the LoE of studies that had not reported ACDF-related complications based on any of the number of fused levels, use of either cage or graft, implant characteristics, or ACDF technique, was downgraded by 1 grade. In order to observe all the possible complications following the ACDF, a minimum of 90 days was considered as the adequate follow-up duration. Studies with a follow-up duration of less than 90 days or no report of follow-up duration were downgraded by 1 grade accordingly. In case of disagreement between reviewers, another senior author performed the extraction and review independently and discussed the results with reviewers to reach a consensus.

### Statistical analysis

Data extraction and curation were performed using Excel software (Microsoft Corp., Redmond, WA, USA), and data analyses were performed using “metafor” and “meta” packages in the “R” version 3.6.3 (R Foundation for Statistical Computing, Vienna, Austria). For the overall complication rate and each complication individually, the meta-analysis was performed, and the pooled proportion with a 95% confidence interval (CI) was calculated. Chi-square test and I2 statistics were used to evaluate the statistical heterogeneity among studies, and substantial heterogeneity was considered as a p-value ≤ .1 or an I2 >50% in the present study. All meta-analyses in this study were conducted via the random effect model and DerSimonian and Laird method. The minimum number of studies required to perform a meta-analysis was 3 in the present study. p-values < .05 were considered statistically significant in this study. Potential publication bias in each meta-analysis was also evaluated using Egger's regression test for asymmetry of the funnel plot.

In addition to the overall meta-analysis, subgroup analysis was performed based on the number of levels of fusion (1-, 2-, 3-, and 4-level), implant characteristics (structural allograft, titanium cage, and PEEK cage), and ACDF technique (use of cage without plating system, use of plating system with cage/graft, and use of standalone or integrated cages). For each subgroup, complication rates (pooled proportions) were calculated, similar to the meta-analysis of the total population. Furthermore, meta-regression was performed using generalized linear mixed models (GLMMs) with a binomial probability distribution on potential predicting factors, including age, number of fused levels, and surgical or implant characteristics. Logit-transformed proportions were used as the outcome measure for GLMM, and odds ratios with 95% CIs and p-values were estimated to evaluate the significance of the correlation between different potential predictors and complication rates. The Omnibus test was also performed to evaluate the significance of the whole model. Additionally, to evaluate the potential effect of study quality, including high- (LoE I/II) and low-quality (LoE III/IV) on the overall ACDF-related complication rate, a sensitivity analysis was performed through meta-regression.

## Results

A total of 2,558 publications were initially identified through the systematic review of the literature, and after initial screening based on title and abstract, a total of 388 articles remained for full-text review. Finally, based on the eligibility criteria, a total of 222 studies were selected for data extraction and meta-analysis. Figure includes a flow diagram demonstrating the study process.

**Figure:**
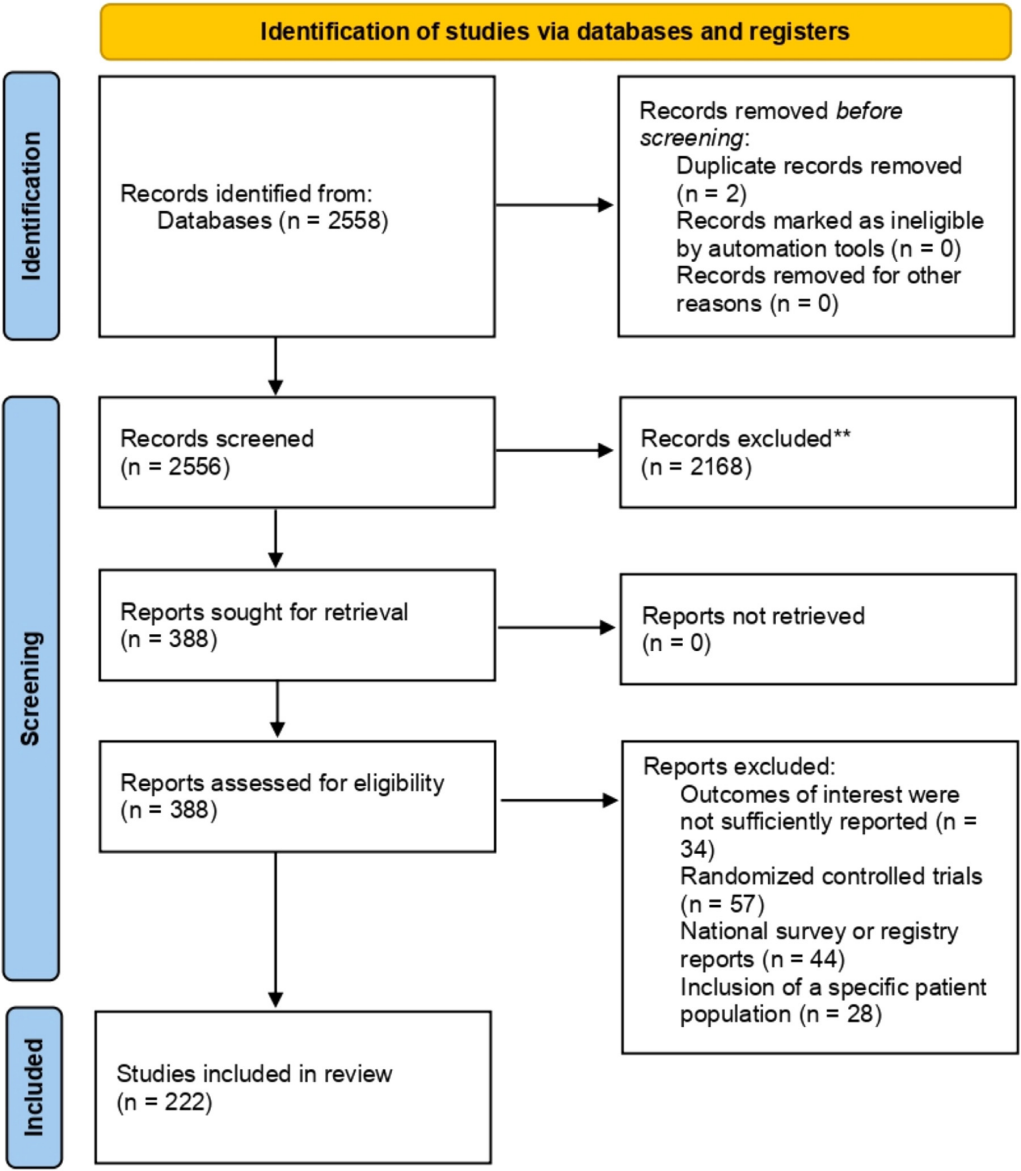
The PRISMA workflow of the study.

### Study characteristics

The selected studies included 51 prospective cohort studies and 171 retrospective cohort studies (references have been included in the supplementary material). A total of 50,584 patients in 267 cohorts were included in the present meta-analysis (Table 1). Studies were published between 1996 and 2024 and recruited patients from 24 countries. The median follow-up duration of the studies included in this meta-analysis was 16.7 months (IQR, 6.0–26.9). The weighted mean age of patients was 53.1 years ranging from 39 to 68 years among included studies. The overall percentage of male patients in this meta-analysis was 52.0% ranging from 0.4 to 100.0% among included studies. The mean LOS in patients was 4.6 days ranging from 0.3 to 15.4 days among included studies.

**Table 1:** Characteristics of included studies.

Characteristics	Value	No. of patients included in the analysis
No. of included studies	222	50,584
No. of included cohorts	267	-
Level of evidence – No. of studies		
1	2	523
2	70	12,055
3	102	15,068
4	48	22,938
Study types – No. of studies		
Prospective cohort	51	7,871
Retrospective cohort	171	42,713
Loss to follow-up – Percentage	4.2%	22,341
Follow-up duration – Mean (SD)	28.9 (3.1)	34,556
Age – Mean (SD)	53.1 (0.4)	33,537
Sex – percentage of males	52.0%	43,334
LOS – Mean (SD)	4.6 (0.6)	14,695
Number of levels of fusion – No. of studies		
1-level	44	4,633
2-level	32	3,270
3-level	24	1,459
4-level	8	432
Implant characteristics – No. of studies		
Structural allograft	14	1,716
Titanium cage	10	1,054
PEEK cage	66	4,920
ACDF technique – No. of studies		
Cage alone	31	1,971
Cage with plating system	81	7,076
Standalone/integrated cage	52	2,646

LOS, length of stay.

### Quality assessment

Based on the methodological quality assessment, 2 (0.9%) studies provided level I evidence, 70 (31.5%) provided level II evidence, 102 (45.9%) provided level III evidence, and 48 (21.6%) provided level IV evidence. Therefore, given the high percentage of studies with level III/IV evidence (67.5%), the overall quality of the included studies was poor. The poor study design was the source of bias in 171 (77.0%) studies, as they were retrospective cohorts. A total of 99 (44.6%) studies had not mentioned prognostic factors or complication rates for subgroups based on potential risk factors. Further, the follow-up duration was less than 3 months in 77 (34.7%) studies. A total of 4 (1.8%) studies also had a loss to follow-up rate of more than 20% in this meta-analysis. The Egger precision-weighted regression test (bias coefficient = 0.0420, p = .9810) demonstrated the symmetry of funnel plot of the overall ACDF-related postoperative complication rate with no evidence of publication bias (Supplementary Fig. 1). Nevertheless, given the overall poor quality (level III/IV evidence) of the majority of included studies, subgroup analysis, meta-regression of various potential risk factors, and sensitivity analysis were performed to minimize the heterogeneity. Sensitivity analysis demonstrated no significant association between study quality and overall postoperative complication rate (p = .3856). Supplementary Table 1 demonstrates the rate of postoperative ACDF-related complications based on the quality of studies (level I/II vs. level III/IV evidence).

### Total postoperative complication rates

A total of 212 studies reported overall ACDF-related complication rates and included 47,172 patients among which a total of 6,157 cases of postoperative complications occurred. The pooled overall postoperative ACDF-related complication rate was 16.0% (95% CI, 13.9%–18.1%) in the total study population. The most common postoperative complications were excessive neck swelling (11.3% [95% CI, 2.4%–20.1%]), pseudarthrosis (10.0% [95% CI, 7.7%–12.4%]), dysphagia (9.5% [95% CI, 7.3%–11.6%]), cage/graft subsidence (9.4% [95% CI, 5.4%–13.4%]), worsening myelopathy (7.7% [95% CI, 2.1%–13.3%]), hoarseness (2.3% [95% CI, 1.7%–3.0%]), C5 nerve root palsy (2.1% [95% CI, 1.5%–2.6%]), recurrent laryngeal nerve (RLN) palsy (2.0% [95% CI, 0.9%–3.1%]), pulmonary complications (1.5% [95% CI, 1.0%–2.1%]), feeding tube placement requirement (1.5% [95% CI, 0.4%–2.7%]), and pneumonia (1.3% [95% CI, 0.7%–2.0%]). The pooled postoperative rates of nonhome discharge, readmission, and mortality in the total included population were 13.8% (95% CI, 0.0%–32.7%), 3.7% (95% CI, 2.5%–4.9%), and 0.1% (95% CI, 0.1%–0.2%), respectively (Table 2). Supplementary Figs. 2-31 demonstrate the forest plots for all meta-analyses performed on the total study population.

**Table 2:** Incidence rates of postoperative complications in the total study population.

	Number of studies	Number of incidents	Subgroup size	Rate (%)	I^2^
Dysphagia	110	1,889	22,405	9.46 (7.32–11.6)	99.33%
Pseudarthrosis	92	1,492	14,106	10.04 (7.71–12.38)	99.32%
Wound dehiscence	16	66	12,347	0.23 (0.13–0.32)	17.4%
General SSI rate	68	199	24,878	0.61 (0.45–0.76)	66.72%
Superficial SSI	47	116	11,170	0.71 (0.48–0.95)	52.41%
Deep SSI/abscess	27	13	7,531	0.15 (0.06–0.23)	0%
Wound hematoma/seroma	41	153	10,950	1.15 (0.76–1.54)	81.31%
Epidural hematoma	17	26	4,750	0.57 (0.27–0.87)	20.92%
Horner's syndrome	12	26	7,180	0.18 (0.08–0.28)	0%
RLN palsy	24	147	6,366	2.04 (0.93–3.14)	97.55%
Hoarseness	39	205	8,612	2.34 (1.69–2.99)	87.75%
Cage/graft subsidence	51	461	13,237	9.39 (5.43–13.35)	99.98%
C5 nerve root palsy	24	78	2,992	2.05 (1.48–2.62)	12.07%
CSF leak	49	131	12,230	0.67 (0.49–0.86)	27.6%
Pneumonia	21	179	9,847	1.34 (0.73–1.96)	94.82%
Pulmonary complications	19	183	11,181	1.53 (1.01–2.06)	87.08%
Pharyngeal/esophageal perforation	14	11	5,213	0.14 (0.03–0.24)	1.78%
Feeding tube placement requirement	3	8	422	1.51 (0.35–2.66)	0.31%
DVT	17	61	7,836	0.57 (0.29–0.86)	68.8%
PE	18	29	8,303	0.28 (0.13–0.42)	28.87%
VTE	8	24	7,153	0.22 (0.09–0.36)	32.41%
Worsening myelopathy	13	92	1,936	7.68 (2.07–13.3)	99.52%
Worsening radiculopathy	29	60	6,941	0.37 (0.16–0.57)	34.89%
Excessive neck swelling	11	336	2,908	11.25 (2.38–20.11)	99.6%
UTI	21	127	13,276	0.72 (0.46–0.98)	74.55%
Cardiac complications	21	102	13,107	0.2 (0.12–0.28)	0%
Nonhome discharge	9	301	2,875	13.8 (0.00–32.68)	99.97%
Readmission	30	594	13,664	3.69 (2.49–4.89)	96.58%
Mortality	27	95	17,230	0.12 (0.07–0.17)	0%
Overall complication rate	212	6157	47,172	16 (13.89–18.1)	99.37%

CSF, cerebrospinal fluid; DVT, deep venous thrombosis; PE, pulmonary embolism; RLN, recurrent laryngeal nerve; SSI, surgical site infection; UTI, urinary tract infection.

### Complication rates and number of fused levels

A total of 70 studies reported the rate of postoperative ACDF-related complications, including 1,849 cases in 9,652 patients based on the number of fused levels. The pooled overall postoperative complication rate for 1-level ACDF was 20.5% (95% CI, 15.7%–25.2%). This rate was 20.3% (95% CI, 14.3%–26.3%) for 2-level ACDF, 28.0% (95% CI, 22.6%–33.5%) for 3-level ACDF, and 53.0% (95% CI, 29.5%–76.5%) for 4-level ACDF (Table 3). Meta-regression showed a significant association between the increase in the number of fused levels and an increase in the postoperative rate of dysphagia (p = .0003), pseudarthrosis (p = .0073), feeding tube placement requirement (p = .0132), worsening myelopathy (p = .0006), worsening radiculopathy (p < .0001), and overall complication rate (p =.0015). More fused levels, however, were associated with lower incidence rates of cage/graft subsidence (p = .0004) (Table 4).

**Table 3:** Incidence rates of postoperative complications based on the number of fused levels.

	1-level		2-level		3-level		4-level	
	Rate	Number	Rate	Number	Rate	Number	Rate	Number
Dysphagia	4.81 (2.83–6.79)	151/2,828	10.54 (5.99–15.09)	199/2,281	16.94 (10.79–23.09)	185/1,132	34.21 (9.04–59.37)	23/343
Pseudarthrosis	9.68 (6.74–12.61)	244/2,245	13.27 (7.63–18.91)	237/1,564	13.83 (7.77–19.89)	175/980	40.27 (17.04–63.49)	61/127
General SSI rate	0.23 (0.00–0.49)	2/1,259	0.31 (0.00–0.71)	2/708	1.52 (0.03–3.01)	1/254	-	-
Superficial SSI	1.79 (0.34–3.24)	1/309	-	-	0.00 (0.00–3.35)	0/183	-	-
Deep SSI/abscess	0.00 (0.00–3.72)	0/178	-	-	1.52 (0.03–3.01)	1/254	-	-
Wound hematoma/seroma	2.2 (0.44–3.97)	38/1,602	2.05 (0.00–4.39)	25/1,064	2.98 (1.06–4.9)	8/297	-	-
Epidural hematoma	0.2 (0.00–0.49)	2/953	-	-	-	-	-	-
Cage/graft subsidence	22.42 (16.31–28.54)	303/1,668	12.53 (4.92–20.13)	57/789	1.87 (0.4–3.34)	19/519	-	-
C5 nerve root palsy	-	-	-	-	1.62 (0.00–4.29)	3/133	-	-
CSF leak	0.15 (0.00–0.36)	4/1,469	0.25 (0.00–0.57)	3/938	0.8 (0.00–2.14)	3/308	-	-
Pulmonary complications	1.12 (0.46–1.79)	12/964	0.53 (0.00–1.1)	3/646	-	-	-	-
Pharyngeal/esophageal perforation	0.12 (0.00–0.3)	1/1,376	0.25 (0.00–0.57)	3/938	-	-	-	-
Worsening radiculopathy	0.18 (0.00–0.44)	1/1,001	0.39 (0.00–0.88)	3/630	2.35 (0.00–4.94)	3/129	-	-
Readmission	1.38 (0.64–2.11)	14/964	0.73 (0.07–1.38)	9/646	-	-	-	-
Mortality	-	-	0.28 (0.00–0.69)	1/619	-	-	-	-
Overall complication rate	20.47 (15.73–25.22)	783/4,547	20.3 (14.31–26.29)	550/3,214	28.02 (22.55–33.49)	420/1,459	52.97 (29.48–76.46)	96/432

CSF, cerebrospinal fluid; SSI, surgical site infection.

**Table 4:** Results of meta-regression analysis evaluating the association between number of fused levels and postoperative complication rates.

	Number of fused levels*		1-level vs. 2-level†		1-level vs. 2 or more levels†		1 or 2 levels vs. 3 or 4 levels†		2-level vs. 3 or 4 levels†	
	OR (95% CI)	p	OR (95% CI)‡	p	OR (95% CI)§	p	OR (95% CI)║	p	OR (95% CI)¶	p
Dysphagia	2.0 (1.3–3.0)	**.0003**#	2.2 (1.0–5.5)	.0565	3.7 (1.6–8.2)	**.0019**#	3.3 (1.5–6.7)	**.0023**#	2.0 (.9–5.0)	.1032
Pseudarthrosis	1.5 (1.1–2.0)	**.0073**#	1.3 (0.7–2.7)	.4302	1.8 (0.9–3.3)	.0761	2.0 (1.0–3.7)	**.0419**#	1.6 (0.7–3.7)	.2129
General SSI rate	2.2 (1.0–5.5)	.06	1.8 (0.2–12.2)	.5645	2.5 (0.4–13.5)	.2793	3.7 (0.7–20.1)	.1463	2.5 (0.4–18.2)	.3525
Superficial SSI	0.7 (0.1–4.1)	.6673	4.1 (0.2–66.7)	.3289	1.2 (0.1–18.2)	.9048	-	-	-	-
Deep SSI/abscess	10.0 (0.7–148.4)	.0885	-	-	-	-	-	-	-	-
Wound hematoma/seroma	1.2 (0.4–4.1)	.742	1.6 (0.1–20.1)	.6912	1.5 (0.2–10)	.6817	1.2 (0.1–11.0)	.8917	0.9 (0.1–10.0)	.9587
Epidural hematoma	0.7 (0.1–6.0)	.7389	0.8 (0.1–9.0)	.8779	0.7 (0.1–8.2)	.8075	-	-	-	-
RLN palsy	1.1 (0.7–1.6)	.758	1.1 (0.5–2.2)	.8634	1.1 (0.5–2.2)	.7944	1.1 (0.4–2.7)	.7963	1.1 (0.4–3.0)	.87
Hoarseness	0.5 (0.2–1.5)	.2316		-	0.3 (0.0–1.8)	.1783	0.5 (0.1–3.0)	.4179	0.9 (0.1–10.0)	.9132
Cage/graft subsidence	0.3 (0.2–0.6)	**.0004**#	0.4 (0.1–1.2)	.1035	0.2 (0.1–0.5)	**.0017**#	0.1 (0.0–0.4)	**.0017**#	0.2 (0.0–1.5)	.1117
C5 nerve root palsy	1.0 (0.3–0.4)	.9616	-	-	0.7 (0.1–6.7)	.7987	0.7 (0.1–6.7)	.7987	-	-
CSF leak	2.0 (0.7–6.0)	.21	1.1 (0.2–6.7)	.8884	2.2 (0.3–18.2)	.4542	4.1 (0.6–24.5)	.1403	3.7 (0.4–30.0)	.2175
Pulmonary complications	0.7 (0.2–1.8)	.4484	0.4 (0.1–1.3)	.1248	0.5 (0.2–1.5)	.218	22.2 (2.5–200.3)	**.0066**#	44.7 (3.7–492.7)	**.0024**#
Pharyngeal/esophageal perforation	1.6 (0.4–6.7)	.468	4.5 (0.4–40.4)	.1989	3.7 (0.4–36.6)	.2572	-	-	-	-
Feeding tube placement requirement	4.5 (1.3–14.9)	**.0132**#	-	-	-	-	12.2 (1.2–121.5)	**.033**#	4.5 (0.4–44.7)	.1874
Worsening myelopathy	3.7 (1.6–7.4)	**.0006**#	-	-	-	-	-	-	-	-
Worsening radiculopathy	4.5 (2.2–9.0)	**<.0001**#	5.0 (0.5–44.7)	.1756	14.9 (1.1–200.3)	**.0405**#	13.5 (3.7–49.4)	**.0001**#	6.7 (1.6–30.0)	**.0083**#
Excessive neck swelling	0.8 (0.1–9.0)	.8433	0.8 (0.1–9.0)	.8433	0.8 (0.1–9.0)	.8433	-	-	-	-
Nonhome discharge	1.6 (0.3–9.0)	.5996	-	-	-		-	-	-	-
Readmission	1.6 (0.9–3.0)	.079	1.0 (0.2–4.1)	.9925	1.5 (0.3–6.0)	.6081	4.5 (1.1–18.2)	**.0392**#	4.1 (0.5–33.1)	.2022
Overall complication rate	1.5 (1.2–2.0)	**.0015**#	1.1 (0.6–2.0)	.7662	1.6 (1.0–2.7)	.0511	2.5 (1.3–4.1)	**.0016**#	2.2 (1.2–4.1)	.0098*

P values less than 0.05 were considered significant, and all of them are correct.

CSF, cerebrospinal fluid; OR, odds ratio; RLN, recurrent laryngeal nerve; SSI, surgical site infection.

⁎ Number of fused levels was considered as a continuous variable, and the odds ratio represents the increase or decrease in odds for every 1 unit change in the number of fused levels.

† Number of fused levels was considered as a categorical variable, and the odds ratio represents the comparison of odds between the 2 subgroups.

‡ 1-level subgroup was the reference.

§ 1-level subgroup was the reference.

║ 1- or 2-level subgroup was the reference.

¶ 2-level subgroup was the reference.

# Indicates statistically significant.

No significant difference was found between 1- and 2-level ACDF in the overall complication rate (p = .7662) or each complication. A significant difference was found in the rate of dysphagia (p = .0019), cage/graft subsidence (p =.0017), and worsening radiculopathy (p = .0405) between 1-level ACDF and 2 or more levels. Overall complication rate, however, was not significantly different (p = .0511) between 1-level ACDF and 2- or more levels. In the comparison between 1- or 2-level and 3- or 4-level ACDF, postoperative rates of dysphagia (p = .0023), pseudarthrosis (p = .0419), cage/graft subsidence (p = .0017), pulmonary complications (p = .0066), feeding tube placement requirement (p = .033), worsening radiculopathy (p = .0001), readmission (p = .0392), and overall complications (p =.0016) were significantly different between the 2 subgroups. The postoperative rates of pulmonary complications (p = .0024), worsening radiculopathy (p = .0083), and overall complications (p = .0098) were significantly different between the 2-level ACDF and 3 or more levels (Table 4).

### Complication rates and implant characteristics

A total of 76 studies reported postoperative complication rates (1775/7038) based on the type of implant used in ACDFIn terms of implant type, the pooled overall complication rate was 27.3% (95% CI, 18.6%–36.0%) for the structural allograft subgroup, 27.1% (95% CI, 9.6%–44.6%) for the titanium cage subgroup, and 23.7% (95% CI, 19.8%–27.7%) for the PEEK cage subgroup (Table 5).

**Table 5:** Incidence rates of postoperative complications based on the type of implant used.

	Structural allograft		Titanium cage		PEEK cage	
	Rate	Number	Rate	Number	Rate	Number
Dysphagia	22.66 (3.2–42.12)	63/247	-	-	11.72 (9.14–14.31)	456/3,370
Pseudarthrosis	24.95 (15.14–34.76)	363/1,373	11.09 (0.69–21.49)	39/368	12.13 (8.78–15.48)	364/2,554
Deep SSI/abscess	-	-	-	-	0.00 (0.00–2.34)	0/531
Wound hematoma/seroma	-	-	-	-	1.36 (0.48–2.23)	5/655
Epidural hematoma	-	-	-	-	1.38 (0.29–2.46)	3/436
Horner's syndrome	-	-	-	-	0.83 (0.00–1.7)	2/414
Hoarseness	-	-	-	-	2.5 (1.58–3.43)	40/1,124
Cage/graft subsidence	-	-	9.90 (3.28–16.53)	38/368	15.61 (10.69–20.54)	256/1,508
C5 nerve root palsy	-	-	-	-	2.15 (0.38–3.92)	5/253
CSF leak	-	-	-	-	0.95 (0.28–1.61)	6/805
Feeding tube placement requirement	-	-	-	-	1.83 (0.00–3.79)	8/300
Worsening radiculopathy	-	-	-	-	1.69 (0.55–2.84)	15/471
Readmission	-	-	-	-	3.62 (2.06–5.18)	22/549
Overall complication rate	27.28 (18.55–36.01)	494/1,716	27.12 (9.62–44.62)	111/422	23.73 (19.82–27.65)	1,170/4900

CSF, cerebrospinal fluid; SSI, surgical site infection.

A significant difference was found between the structural allograft and cage (including both titanium and PEEK cages) subgroups in the postoperative incidence rates of dysphagia (p < .0001), pseudarthrosis (p < .0001), cage/graft subsidence (p < .0001), and overall complications (p < .0001). No significant difference was found between the titanium and PEEK cage subgroups in the pooled overall postoperative complication rate (p = .8734) and specific complication rates (Table 6).

**Table 6:** Results of meta-regression analysis evaluating the association between the type of implant used and postoperative complication rates.

	Structural allograft vs. cage*		Titanium vs. PEEK cage*	
	OR (95% CI)†	p	OR (95% CI)‡	p
Dysphagia	2.2 (1.6–2.9)	**<.0001**§	0.7 (0.1–3.3)	.6402
Pseudarthrosis	2.2 (1.9–2.6)	**<.0001**§	1.5 (0.4–5.0)	.5462
General SSI rate	-	-	0.2 (0.0–3.7)	.2873
Wound hematoma/seroma	0.7 (0.1–5.8)	0.7396	-	-
Hoarseness	0.7 (0.3–1.8)	0.4489	-	-
Cage/graft subsidence	2.7 (1.9–3.7)	**<.0001**§	1.5 (0.4–5.5)	.5328
Worsening radiculopathy	-	-	4.1 (0.2–73.7)	.3596
Readmission	0.3 (0.0–2.0)	.1968	2.2 (0.3–16.4)	.4418
Overall complication rate	1.3 (1.1–1.4)	**<.0001**§	1.1 (0.4–3.3)	.8734

P values less than 0.05 were considered significant, and all of them are correct.

CSF, cerebrospinal fluid; OR, odds ratio; SSI, surgical site infection.

⁎ Odds ratios represent the comparison of odds between the 2 subgroups.

† Cage subgroup was the reference.

‡ PEEK subgroup was the reference.

§ Indicates statistically significant.

### Complication rates and ACDF technique

A total of 102 studies reported the incidence rate of postoperative ACDF-related complication rates, including 2615 cases among a total of 11,000 patients based on the technique of ACDF surgery. The pooled overall postoperative complication rates for ACDF using cage alone, cage/graft with plating system, and standalone/integrated cages were 27.8% (95% CI, 20.5%–35.1%), 23.6% (95% CI, 19.7%–27.5%), and 20.0% (95% CI, 14.1%–23.8%), respectively (Table 7).

**Table 7:** Incidence rates of postoperative complications based on the surgical technique.

	Cage without plate		Cage with plating system		Standalone/integrated cage	
	Rate	Number	Rate	Number	Rate	Number
Dysphagia	4.5 (1.87–7.13)	32/480	17.49 (14.09–20.9)	779/3,822	2.97 (2.13–3.81)	113/1,960
Pseudarthrosis	19.15 (13.05–25.25)	198/939	12.86 (9.03–16.69)	681/3,995	8.06 (5.18–10.95)	131/1,225
General SSI rate	1.17 (0.00–2.57)	1/221	0.43 (0.06–0.81)	6/1,155	0.88 (0.3–1.47)	7/979
Superficial SSI	1.59 (0.00–3.55)	1/153	0.44 (0.00–0.95)	3/748	1.27 (0.45–2.09)	3/697
Deep SSI/abscess	-	-	0.00 (0.00–1.33)	0/394	1.62 (0.48–2.77)	4/451
Wound hematoma/seroma	-	-	0.64 (0.21–1.07)	14/1,306	1.43 (0.27–2.6)	2/388
Epidural hematoma	-	-	1.49 (0.09–2.89)	3/283	0.00 (0.00–2.71)	0/247
Hoarseness	-	-	4.7 (2.57–6.83)	71/1,186	1.87 (0.6–3.14)	12/422
Cage/graft subsidence	23.24 (13.79–32.69)	160/681	11.06 (6.45–15.68)	128/1,294	19.93 (11.31–28.55)	184/1,060
C5 nerve root palsy	-	-	2.4 (0.64–4.15)	7/289	1.87 (0.00–4.37)	1/110
CSF leak	-	-	1.12 (0.43–1.82)	16/1,054	0.96 (0.08–1.85)	4/461
Feeding tube placement requirement	-	-	1.83 (−0.14 to 3.79)	8/300	-	-
Worsening radiculopathy	-	-	0.4 (0.00–0.84)	7/826	1.07 (0.05–2.09)	8/396
Readmission	-	-	2.76 (1.07–4.44)	44/1,276	-	-
Mortality	-	-	0.00 (0.00–0.71)	0/679	-	-
Overall complication rate	27.82 (20.53–35.12)	407/1451	23.63 (19.73–27.53)	1737/6,944	18.95 (14.07–23.83)	471/2,605

CSF, cerebrospinal fluid; SSI, surgical site infection.

The meta-regression demonstrated a significantly higher incidence rate of postoperative pseudarthrosis (p = .0097) in patients who underwent ACDF with the cage without a plating system compared with the 2 other techniques. The postoperative incidence rate of dysphagia (p = .0002) in ACDF using the cage/graft with a plating system was significantly higher than the 2 other subgroups. This subgroup, however, was significantly associated (p = .0497) with a lower rate of cage/graft subsidence compared with the other techniques. The use of standalone or integrated cages was significantly associated (p < .0001) with a lower incidence rate of postoperative dysphagia compared with other techniques. No significant difference (p > .05) was found between the ACDF techniques in the pooled overall incidence rate of postoperative complications (Table 8).

**Table 8:** Results of meta-regression analysis evaluating the association between the surgical technique and postoperative complication rates.

	Cage without plate*		Cage with plating system*		Standalone/integrated cage*	
	OR (95% CI)†	p	OR (95% CI)†	p	OR (95% CI)†	p
Dysphagia	0.5 (0.2–1.3)	.1714	2.5 (1.6–4.1)	**.0002**‡	0.3 (0.2–0.4)	**<.0001**‡
Pseudarthrosis	2.5 (1.2–4.5)	**.0097**‡	0.7 (0.4–1.1)	.1277	0.7 (0.3–1.3)	.2395
General SSI rate	0.5 (0.0–6.7)	.6319	0.5 (0.1–3.3)	.4515	1.3 (0.2–7.4)	.7311
Superficial SSI	1.6 (0.1–27.1)	.7263	0.6 (0.1–5.5)	.671	1.0 (0.1–7.4)	.981
Wound hematoma/seroma	-	-	3.0 (0.5–18.2)	.2067	0.4 (0.1–2.7)	.3728
Epidural hematoma	1.5 (0.4–5.5)	.548	0.9 (0.2–4.1)	.909	-	-
RLN palsy	-	-	0.9 (0.1–14.9)	.9601	0.7 (0.0–12.2)	.8203
Hoarseness	0.9 (0.2–3.7)	.9326	1.3 (0.6–3.3)	.4026	0.5 (0.2–1.1)	.0994
Cage/graft subsidence	2.5 (0.8–7.4)	.0943	0.4 (0.1–1.0)	**.0497**‡	1.2 (0.4–3.3)	.7356
C5 nerve root palsy	-	-	0.9 (0.2–4.5)	.9227	0.4 (0.0–3.0)	.3545
CSF leak	0.7 (0.4–5.0)	.5601	1.2 (0.5–2.7)	.6876	0.5 (0.2–1.6)	.2534
Worsening radiculopathy	1.3 (0.1–27.1)	.8609	1.5 (0.4–13.5)	.7224	1.5 (0.2–13.5)	.7331
Nonhome discharge	-	-	0.2 (0.0–3.7)	.2598	0.3 (0.0–16.4)	.5673
Readmission	-	-	1.5 (0.4–5.5)	.5998	0.8 (0.1–5.5)	.8529
Overall complication rate	1.5 (0.8–2.5)	.1657	1.0 (0.7–1.5)	.8996	0.7 (0.4–1.1)	.0992

P values less than 0.05 were considered significant, and all of them are correct.

CSF, cerebrospinal fluid; OR, odds ratio; RLN, recurrent laryngeal nerve; SSI, surgical site infection.

⁎ Odds ratios represent the comparison of odds between the 2 subgroups.

† Each technique was compared with the others (other techniques were considered as the reference).

‡ Indicates statistically significant.

### Complication rates and age

A total of 173 studies, including a total of 30,634 patients, reported the mean age in addition to postoperative complication rates. The meta-regression demonstrated a significant association between patients’ age and increased postoperative rate of dysphagia (p = .0301), pseudarthrosis (p < .0001), general surgical site infection (SSI) rate (p = .0006), deep SSI/abscess (p = .0002), epidural hematoma (p = .0029), pneumonia (p < .0001), deep vein thrombosis (DVT) (p = .0096), pulmonary embolism (PE) (p = .0275), cardiac complications (p = .0155), nonhome discharge (p < .0001), readmission (p = .0289), and overall complications (p = .0042) (Table 9).

**Table 9:** Results of the meta-regression analysis evaluating the association between the age and postoperative complication rates.

	Age*	
	OR (95% CI)	p
Dysphagia	1.1 (1.0–1.1)	**.0301**†
Pseudarthrosis	1.1 (1.0–1.1)	**<.0001**†
Wound dehiscence	1.1 (0.9–1.3)	.1978
General SSI rate	1.2 (1.1–1.3)	**.0006**†
Superficial SSI	1.0 (0.9–1.1)	.8934
Deep SSI/abscess	1.3 (1.2–1.6)	**.0002**†
Wound hematoma/seroma	1.0 (1.0–1.1)	.1673
Epidural hematoma	1.2 (1.1–1.3)	**.0029**†
Horner's syndrome	1.0 (0.9–1.1)	.9712
RLN palsy	1.0 (0.8–1.1)	.6328
Hoarseness	1.0 (0.9–1.1)	.6962
Cage/graft subsidence	1.1 (0.9–1.3)	.2367
C5 nerve root palsy	1.1 (1.0–1.1)	.1074
CSF leak	1.1 (1.0–1.2)	.2384
Pneumonia	1.3 (1.2–1.3)	**<.0001**†
Pulmonary complications	1.0 (0.9–1.1)	.8764
Pharyngeal/esophageal perforation	1.0 (0.8–1.2)	.8059
Feeding tube placement requirement	0.7 (0.4–1.1)	.1412
DVT	1.1 (1.0–1.2)	**.0096**†
PE	1.1 (1.0–1.2)	**.0275**†
Worsening myelopathy	0.7 (0.4–1.1)	.1371
Worsening radiculopathy	0.8 (0.7–1.0)	.0804
Excessive neck swelling	1.1 (0.6–2.0)	.7259
UTI	1.0 (0.8–1.2)	.9889
Cardiac complications	1.2 (1.0–1.3)	**.0155**†
Nonhome discharge	1.0 (1.0–1.1)	**<.0001**†
Readmission	1.1 (1.0–1.2)	**.0289**†
Mortality	1.0 (0.9–1.1)	.8935
Overall complication rate	1.1 (1.0–1.1)	**.0042**†

P values less than 0.05 were considered significant, and all of them are correct.

CSF, cerebrospinal fluid; DVT, deep venous thrombosis; OR, odds ratio; PE, pulmonary embolism; RLN, recurrent laryngeal nerve; SSI, surgical site infection; UTI, urinary tract infection.

⁎ Age was considered as a continuous variable, and the odds ratio represents the increase or decrease in odds for every 1 unit change in the age.

† Indicates statistically significant.

### Multivariate analysis

Based on results of multivariate analysis (Table 10), the rate of cage/graft subsidence was significantly associated with ACDF using cage/graft with plating system (p = .0084) and number of levels of fusion (p < .0001). No such a significant association was found between the postoperative rate of cage/graft subsidence and the use of structural allograft (p = .1539). The rate of dysphagia was significantly associated with cage/graft with plating system (p = .0242), the use of standalone/integrated cage (p = .0075), the number of levels of fusion (p < .0001), use of structural allograft (p < .0001), and age (p = .0138). Since both structural allograft and cage without a plating system were significantly associated with the rate of pseudarthrosis, 2 separate multivariate regression analyses were performed to find potential predictors. In the first model, the postoperative rate of pseudarthrosis was significantly associated with age (p = .0033) and the number of levels of fusion (p = .0194), yet no such a significant association with the structural allograft use (p = .7188) was observed. In the second model, the postoperative rate of pseudarthrosis was significantly associated with age (p = .0388), the number of levels of fusion (p < .0001), and cage without a plating system (p < .0001). The overall postoperative complication rate was significantly associated with the number of levels of fusion (p = .0032) and age (p = .0048). However, no significant association was found between the overall complication rate and use of structural allograft (p = .5725) in the multivariate analysis.

**Table 10:** Results of the multivariate meta-regression analysis.

	Cage/graft subsidence*		Dysphagia*		Pseudarthrosis (first model)*		Pseudarthrosis (first model)*		Overall complication rate*	
	OR (95% CI)	p	OR (95% CI)	p	OR (95% CI)	p	OR (95% CI)	p	OR (95% CI)	p
Number of fused levels†	0.4 (0.3–0.5)	**<.0001**¶	2.0 (1.5–2.5)	**<.0001**¶	1.2 (1.0–1.5)	**.0194**¶	1.6 (1.3–2.0)	**<.0001**¶	1.2 (1.1–1.4)	**.0032**¶
Use of structural allograft‡	1.9 (0.8–4.7)	.1539	6.4 (2.9–14.5)	**<.0001**¶	0.9 (0.4–2.0)	.7188	-	-	0.9 (0.4–2.0)	.5725
Age§	-	-	1.1 (1.0–1.1)	**.0138**¶	1.1 (1.0–1.1)	.0033*	1.0 (1.0–1.1)	**.0388**¶	1.0 (1.0–1.1)	**.0048**¶
Cage without a plating system║	-	**-**	-	-	-	-	4.1 (2.8–6.0)	**<.0001**¶	-	-
Cage/graft with plating system║	0.6 (0.4–0.9)	**.0084**¶	1.7 (1.1–2.8)	**.0242**¶	-	-	-	**-**	**-**	**-**
Use of standalone/integrated cage║	-	-	0.5 (0.3–0.8)	**.0075**¶	-	-	-	-	-	-

P values less than 0.05 were considered significant, and all of them are correct.

OR, odds ratio.

⁎ The multivariate analysis was performed on variables with significant association based on the univariate meta-regression.

† Number of fused levels was considered as a continuous variable, and the odds ratio represents the increase or decrease in odds for every 1 unit change in the number of fused levels.

‡ Odds ratios represent the comparison of odds between the 2 subgroups (cage subgroup was the reference).

§ Age was considered as a continuous variable, and the odds ratio represents the increase or decrease in odds for every 1 unit change in the age.

║ Each technique was compared with the others (other techniques were considered as the reference).

¶ Indicates statistically significant.

## Discussion

In this meta-analysis, the pooled incidence rate of different postoperative ACDF-related complications was estimated based on several previously published cohort studies. The overall pooled incidence rate of postoperative ACDF-related complications was 16%. Moreover, the meta-regression evaluated the association between various potential risk factors and different postoperative complications. In this regard, more levels of fusion and increased age were significantly associated with an increase in the pooled overall postoperative complication rate. Some complications also showed a significant association with a number of perioperative characteristics.

The overall morbidity rate of ACDF in prior reports has been ranging between 13.2% and 19.3%, according to a previous review [8]. Moreover, a meta-analysis including 107 studies (*n* = 8,612) demonstrated an overall rate of 20.1% for complications after cervical spine surgery for cervical compressive myelopathy [17]. However, that study analyzed a heterogeneous population of patients in terms of surgical technique, including corpectomy, ACCF, or laminoplasty, besides ACDF. To our knowledge, the present study has been the most extensive meta-analysis regarding the complications of ACDF specifically.

By descending order, the most common complications after ACDF were excessive neck swelling (11.3%), pseudarthrosis (10.0%), dysphagia (9.5%), cage/graft subsidence (9.4%), worsening myelopathy (7.7%), hoarseness (2.3%), C5 nerve root palsy (2.1%), RLN palsy (2.0%), pulmonary complications (1.5%), feeding tube placement requirement (1.5%), pneumonia (1.3%), and wound hematoma/seroma (1.2%). Merely 2 prior systematic reviews have reported pooled incidence rates of complications for all types of cervical spine surgery and not the ACDF in particular, which prevents us from making an accurate comparison between the results [8,9]. Moreover, high heterogeneity due to inclusion criteria, smaller sample size for a number of complications, high number of eligible articles published recently, and lack of previous pooled data for some complications further limits the comparability of incidence rates.

Based on the results of meta-regression, age was a significant independent risk factor for the incidence rate of various postoperative ACDF-related complications. Previous results have been conflicting regarding the association between age and ACDF-related complications. In a previous analysis of the results of a registry, Lawless et al. [18] found no significant association between age and any complication after ACDF surgery. By contrast, similar to this meta-analysis, Buerba et al. [19] showed that age is an independent predictor for longer hospitalization and greater morbidity, such as respiratory complications, thromboembolism, and overall complications after ACDF. This increase in the incidence of postoperative complications and readmission rates could exert a significant burden on both patients and the healthcare system. Thus, these findings could substantially assist in improving the existing guidelines and also developing plans for cost management, especially in the field of cervical spine surgery, given the increasing trend in the number of these procedures, the age of patients, and associated costs [20,21].

Furthermore, a greater number of fused levels was significantly associated with a higher incidence rate of overall postoperative complications as well as dysphagia, pseudarthrosis, need for feeding tube placement, and worsening myelopathy and radiculopathy. Consistent with our findings, prior reports have demonstrated the correlation between a greater number of fused levels and dysphagia and pseudarthrosis [8,9,12,22,23]. Interestingly, the greater number of fused levels was significantly associated with a lower incidence of postoperative cage/graft subsidence after ACDF surgery in this meta-analysis. This finding might be due in part to the higher percentage of patients undergoing cage without plating in the 1- or 2-level subgroups (19.1% vs. 0.0%) and a lower percentage of those undergoing cage with the plating system (55.9% vs. 76.8%) compared with 3- or 4-level subgroups.

The multivariate analysis also suggested a significant association between the use of cage with a plating system and a lower rate of cage/graft subsidence. Concerning this, previous studies have shown that the cage with plating or the use of screws could significantly reduce the subsidence rate compared with other techniques (e.g., cage alone or standalone cage) [[24], [25], [26], [27]]. Different risk factors for cage subsidence, such as intraoperative distraction, cage type or location, and end-plate preparation have been suggested previously, which might have also contributed to the observed difference between the study subgroups [25,28,29].

Moreover, 3- and 4-level ACDF were significantly associated with higher incidence rates of overall postoperative complications, dysphagia, pseudarthrosis, pulmonary complications, need for feeding tube placement, and worsening radiculopathy and a lower rate of graft/cage subsidence compared with other levels. Similarly, previous reports have shown that multilevel ACDF is an independent risk factor for the feeding tube placement requirement [30,31]. In addition, some risk factors for postoperative pulmonary complications have been demonstrated previously, such as surgical exposure of ≥3 vertebral levels, prolonged operative duration, and myelopathy, which were more common in multilevel ACDF among the included studies [[32], [33], [34]].

The use of structural allograft was also significantly associated with the increased rate of dysphagia compared with the cage use. However, no significant difference was observed in the overall postoperative complication rate as well as cage/graft subsidence and pseudarthrosis rates between the structural allograft and cage subgroups. The titanium and PEEK cages were also comparable in terms of the overall and specific postoperative complication rates in this meta-analysis. Similar to this study, a prior registry, including 17,783 patients, found a significantly higher dysphagia rate in patients undergoing ACDF with structural allograft compared with the synthetic cage group [35]. Likewise, Menon et al. [36], in their report of a national database, including 8,103 patients, observed that patients who underwent ACDF with structural allograft show a significantly higher rate of dysphagia compared with the synthetic cage group. Caution must be taken while interpreting these findings, given the differences in risk factors for dysphagia among the studies, such as the operating time and cervical levels fused [37,38].

Concerning the cage/graft subsidence, although data was limited, multivariate meta-regression analysis demonstrated no significant association between the implant type and its rate. In a similar vein, Peng et al. [39], in a recent meta-analysis, found no significant difference between the structural allograft and PEEK cage in the rate of subsidence. Additionally, regarding the effect of structural allograft use on pseudarthrosis rate, prior studies have shown varying results. Similarly, Menon et al. [36] found no significant difference in pseudarthrosis rate between the structural allograft and cage groups after propensity score matching based on many potential influencing factors and comorbidities. Some studies, however, have demonstrated that the use of structural allograft is significantly associated with a higher rate of pseudarthrosis compared with synthetic cages [[39], [40], [41]]. One possible explanation for this heterogeneity in results is differences in many uncontrolled risk factors among the studies and their potential effects on findings, which mandates further investigation in this regard [36].

Among different ACDF techniques, the cage without plating was significantly associated with a higher incidence rate of pseudarthrosis compared with other techniques. A previous report of a national registry also demonstrated that the use of anterior instrumentation could significantly reduce the risk of pseudarthrosis following cervical spine fusion surgery [42]. In addition, the cage with a plating system was significantly associated with a higher rate of dysphagia. The use of standalone/integrated cages, on the other hand, was significantly associated with a lower rate of dysphagia in this meta-analysis. Similarly, Yang et al. [43], in a previous meta-analysis including 10 studies demonstrated that zero-profile spacer was significantly associated with a lower rate of postoperative dysphagia compared with the cage and plate technique. No significant difference in the overall rate of postoperative complications between the ACDF techniques was found. These findings could significantly help in preoperative planning to avoid potential complications and related costs depending on the patient's risk profile.

Despite its strengths, this meta-analysis had some limitations. First, the included studies were observational, with the majority having a low-quality presenting LoE III/IV. Moreover, substantial heterogeneity exists among the included studies, especially in different characteristics of their populations of patients. Several measures were taken to minimize the source of heterogeneity and effects of low-quality studies. However, a number of potential risk factors, such as surgical indication, obesity, osteoporosis, use of bone morphogenetic protein, operative levels, or inpatient/outpatient ACDF still have not been clearly and adequately reported in prior studies to allow for their inclusion in meta-regression analysis. Additionally, substantial differences exist among studies in reporting and evaluating the number or severity of the complications, which could significantly affect the overall pooled incidence rate. Second, many complications have not been reported previously based on different subgroups, which leads to a small sample size in some subgroup meta-analyses or a lack of subgroup data for a number of complications. This mandates future investigations to provide further evidence regarding the association between complications and perioperative characteristics. Further, merely a limited number of included studies have provided information about all the perioperative characteristics. This significantly prevented us from performing a multivariate analysis for many postoperative complication rates. Finally, the results of this meta-analysis should be interpreted cautiously, given all the aforementioned limitations and sources of bias. These findings could be a guide for designing future clinical trials to address several existing gaps and also improve current guidelines for perioperative practices in ACDF surgery.

## Conclusions

The overall ACDF-related morbidity rate was 16% among the included studies. Excessive neck swelling, pseudarthrosis, dysphagia, cage/graft subsidence, worsening myelopathy, hoarseness, C5 nerve root palsy, and RLN palsy were the most common postoperative ACDF-related complications. Additionally, several perioperative characteristics were significantly associated with the overall and some specific complication rates after the ACDF surgery. To our knowledge, this study has been the most extensive meta-analysis conducted on the existing literature regarding ACDF-related complications and potential risk factors. However, there is still a lack of data concerning the potential risk factors for a number of specific ACDF-related complications. Therefore, future high-quality prospective studies or clinical trials are highly required to provide further evidence and validate the present findings.

## Funding

None.

## Declarations of competing interests

The authors declare that they have no known competing financial interests or personal relationships that could have appeared to influence the work reported in this paper.

## Appendix Supplementary materials

Image, application 1
