# Supplementary material for: Postoperative complications of anterior cervical discectomy and fusion: A comprehensive systematic review and meta-analysis
Source: N Am Spine Soc J. 2025 Feb 8;21:100596. doi: 10.1016/j.xnsj.2025.100596 (PMC11938155; doi:10.1016/j.xnsj.2025.100596)
Supplement: Supplementary file 1 [file mmc1.pdf]

Supplementary Tables

Supplementary Table 1. Incidence rates of postoperative complications based on the quality of studies included.

	Poor-quality studies (LoE III/IV)		High-quality studies (LoE I/II)	
	Number of incidents	Rate (%)	Number of incidents	Rate (%)
Dysphagia	1325/13894	10.90 (7.70 - 14.10)	564/8511	6.80 (4.92 - 8.69)
Pseudarthrosis	888/8966	10.33 (6.80 - 13.87)	604/5140	9.63 (6.79 - 12.47)
Wound dehiscence	65/12247	0.22 (0.13 - 0.32)	-	-
General SSI rate	174/19807	0.68 (0.48 - 0.88)	25/5071	0.24 (0.10 - 0.38)
Superficial SSI	103/9928	0.70 (0.44 - 0.97)	13/1242	0.84 (0.29 - 1.39)
Deep SSI/abscess	11/6928	0.14 (0.05 - 0.22)	2/603	0.96 (0.19 - 1.73)
Wound hematoma/seroma	81/7076	0.83 (0.50 - 1.16)	72/3874	1.36 (0.51 - 2.21)
Epidural hematoma	22/2862	0.66 (0.29 - 1.03)	4/1888	0.37 (0.00 - 0.80)
Horner's syndrome	23/4589	0.39 (0.14 - 0.64)	-	-
RLN palsy	85/2638	2.13 (0.60 - 3.66)	62/3728	1.88 (0.34 - 3.42)
Hoarseness	180/6019	2.86 (1.95 - 3.76)	25/2593	1.34 (0.50 - 2.17)
Cage/graft subsidence	373/10959	10.23 (5.16 - 15.31)	88/2278	6.91 (1.72 - 12.1)
C5 nerve root palsy	68/2618	2.05 (1.42 - 2.68)	10/374	2.18 (0.52 - 3.84)
CSF leak	91/6654	1.11 (0.70 - 1.52)	40/5576	0.64 (0.28 - 1.01)
Pneumonia	177/9221	1.48 (0.76 - 2.19)	2/626	0.19 (0.00 - 0.53)
Pulmonary complications	164/9544	1.61 (1.03 - 2.18)	19/1637	0.96 (0.36 - 1.55)
Pharyngeal/esophageal perforation	7/2373	0.24 (0.02 - 0.46)	4/2840	0.14 (0.00 - 0.31)
Feeding tube placement requirement	4/222	1.25 (0.00 - 2.71)	-	-
DVT	61/7596	0.58 (0.28 - 0.87)	0/240	0.59 (0.00 - 1.56)
PE	28/7673	0.26 (0.12 - 0.41)	1/630	0.33 (0.00 - 0.77)
VTE	24/7053	0.22 (0.08 - 0.35)	-	-
Worsening myelopathy	62/1449	8.51 (0.58 - 16.44)	30/487	6.29 (0.00 - 13.37)
Worsening radiculopathy	43/4690	0.24 (0.07 - 0.40)	17/2251	0.85 (0.21 - 1.50)
Excessive neck swelling	331/1376	12.80 (2.39 - 23.22)	-	-
UTI	115/11074	0.73 (0.45 - 1.01)	12/2202	0.81 (0.00 - 1.71)
Cardiac complications	102/13107	0.20 (0.12 - 0.28)	-	-
Non-home discharge	300/2779	15.22 (0.00 - 36.1)	-	-
Readmission	545/11152	3.95 (2.47 - 5.43)	49/2512	2.06 (1.09 - 3.03)
Mortality	88/13408	0.12 (0.06 - 0.18)	7/3822	0.10 (0.00 - 0.20)
Overall complication rate	4565/34868	17.18 (14.36 – 20.00)	1592/12304	13.37 (10.72 - 16.02)

CSF: cerebrospinal fluid; DVT: deep venous thrombosis; LoE: level of evidence; PE: pulmonary embolism; RLN: recurrent laryngeal nerve; SSI: surgical site infection; UTI: urinary tract infection

Supplementary Figure 1. The funnel plot of the overall ACDF-related postoperative complication rate meta-analysis.

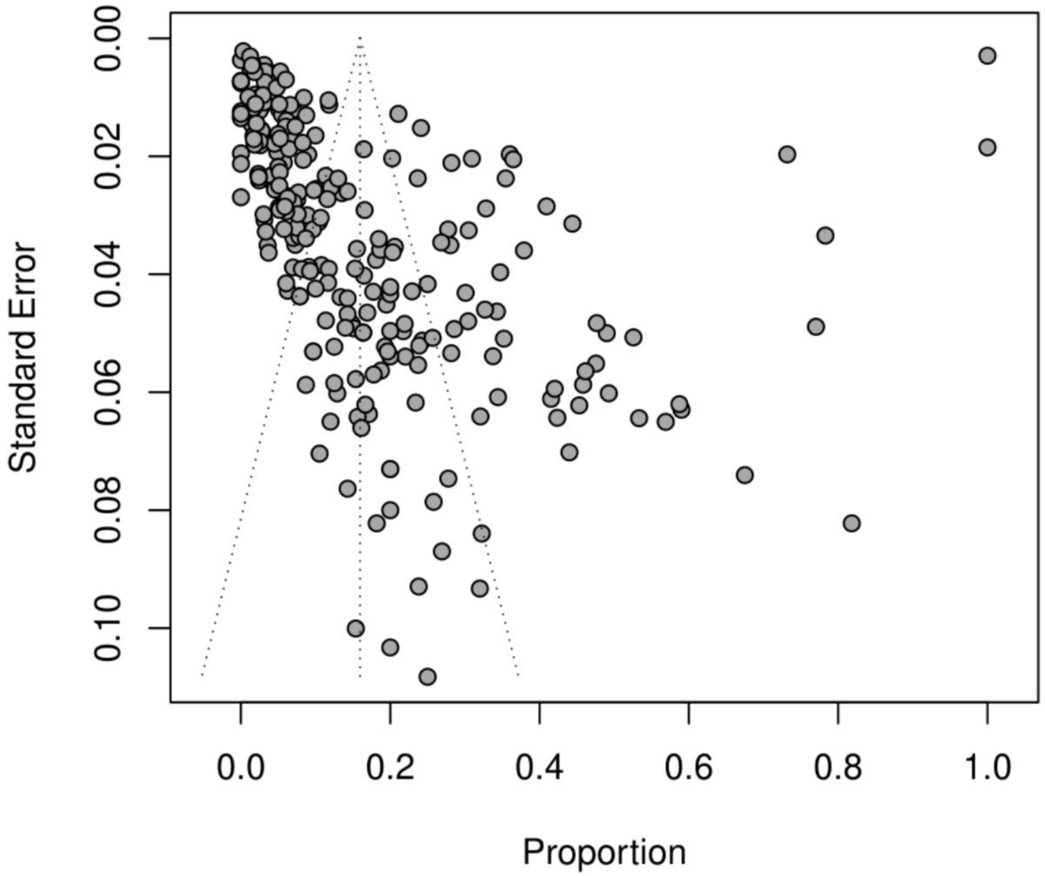

Supplementary Figure 2. Forest plot for dysphagia.

Study	Events	Total	Proportion	95%−CI	Weight
Cauthen et al., 1997	56	514	0.11	[0.08; 0.14]	0.8%
Bose et al., 2001	3	180	0.02	[0.00; 0.05]	0.8%
Bazaz et al., 2002	25	123	0.20	[0.14; 0.29]	0.7%
Tureyen et al., 2003	1	43	0.02	[0.00; 0.12]	0.8%
Nirala et al., 2003	3	69	0.04	[0.01; 0.12]	0.8%
Yue et al., 2004	34	74	0.46	[0.34; 0.58]	0.7%
Bolesta et al., 2005	128	454	0.28	[0.24; 0.33]	0.8%
Korinth et al., 2006	2	124	0.02	[0.00; 0.06]	0.8%
Lied et al., 2006	4	390	0.01	[0.00; 0.03]	0.8%
Mobbs et al., 2007	6	242	0.02	[0.01; 0.05]	0.8%
Villavicencio et al., 2007	6	103	0.06	[0.02; 0.12]	0.8%
Fountas et al., 2007	59	1015	0.06	[0.04; 0.07]	0.8%
Ruetten et al., 2008	3	100	0.03	[0.01; 0.09]	0.8%
Dogan et al., 2008	5	22	0.23	[0.08; 0.45]	0.5%
Tumialán et al., 2008	14	200	0.07	[0.04; 0.11]	0.8%
Ghahreman et al., 2009	2	55	0.04	[0.00; 0.13]	0.8%
Liu et al., 2009	1	109	0.01	[0.00; 0.05]	0.8%
Leach et al., 2009	1	123	0.01	[0.00; 0.04]	0.8%
Scholz et al., 2010	1	38	0.03	[0.00; 0.14]	0.8%
Coric et al., 2010	0	41	0.00	[0.00; 0.09]	0.8%
Song et al., 2010	4	83	0.05	[0.01; 0.12]	0.8%
Joseffer et al., 2010	47	390	0.12	[0.09; 0.16]	0.8%
Rihn et al., 2011	3	38	0.08	[0.02; 0.21]	0.7%
Lin et al., 2011	4	57	0.07	[0.02; 0.17]	0.7%
Iampreechakul et al., 2011	3	67	0.04	[0.01; 0.13]	0.8%
Sheperd et al., 2012	2	152	0.01	[0.00; 0.05]	0.8%
Song et al., 2012	3	25	0.12	[0.03; 0.31]	0.6%
Liu et al., 2012	6	103	0.06	[0.02; 0.12]	0.8%
Liu et al., 2012	8	69	0.12	[0.05; 0.22]	0.7%
Lied et al., 2012	2	96	0.02	[0.00; 0.07]	0.8%
Hofstetter et al., 2013	15	70	0.21	[0.13; 0.33]	0.7%
Lu et al., 2013	22	50	0.44	[0.30; 0.59]	0.6%
Lu et al., 2013	40	100	0.40	[0.30; 0.50]	0.7%
Davis et al., 2013	8	105	0.08	[0.03; 0.14]	0.8%
Barbagallo et al., 2013	13	85	0.15	[0.08; 0.25]	0.7%
Wang et al., 2014	9	63	0.14	[0.07; 0.25]	0.7%
Tracey et al., 2014	3	88	0.03	[0.01; 0.10]	0.8%
Kasliwal et al., 2014	0	59	0.00	[0.00; 0.06]	0.8%
Nanda et al., 2014	52	1576	0.03	[0.02; 0.04]	0.8%

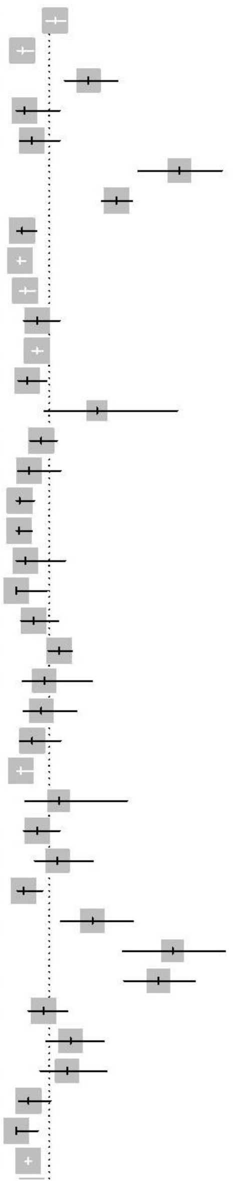

Chen et al., 2015	3	69	0.04	[0.01; 0.12]	0.8%
Wang et al., 2015	4	30	0.13	[0.04; 0.31]	0.6%
Wang et al., 2015	1	27	0.04	[0.00; 0.19]	0.7%
Liu et al., 2016	8	60	0.13	[0.06; 0.25]	0.7%
Chen et al., 2016	2	54	0.04	[0.00; 0.13]	0.8%
De La Garza−Ramos et al., 2016	17	97	0.18	[0.11; 0.27]	0.7%
Wang et al., 2016	0	52	0.00	[0.00; 0.07]	0.8%
Shi et al., 2016	11	65	0.17	[0.09; 0.28]	0.7%
Adamson et al., 2016	16	1478	0.01	[0.01; 0.02]	0.8%
Radcliff et al., 2016	3	25	0.12	[0.03; 0.31]	0.6%
Chin et al., 2016	4	55	0.07	[0.02; 0.18]	0.7%
Lovasik et al., 2016	36	191	0.19	[0.14; 0.25]	0.8%
Shi et al., 2016	4	57	0.07	[0.02; 0.17]	0.7%
Shi et al., 2016	7	68	0.10	[0.04; 0.20]	0.7%
Yang et al., 2016	14	139	0.10	[0.06; 0.16]	0.8%
Alimi et al., 2016	8	104	0.08	[0.03; 0.15]	0.8%
ElAbed et al., 2016	0	90	0.00	[0.00; 0.04]	0.8%
Qizhi et al., 2016	1	16	0.06	[0.00; 0.30]	0.6%
Li et al., 2017	3	31	0.10	[0.02; 0.26]	0.7%
Fisahn et al., 2017	14	211	0.07	[0.04; 0.11]	0.8%
Fisahn et al., 2017	20	166	0.12	[0.08; 0.18]	0.8%
Riederman et al., 2017	9	36	0.25	[0.12; 0.42]	0.6%
Riederman et al., 2017	21	96	0.22	[0.14; 0.31]	0.7%
Riederman et al., 2017	42	164	0.26	[0.19; 0.33]	0.7%
Riederman et al., 2017	30	104	0.29	[0.20; 0.39]	0.7%
Bucci et al., 2017	2	110	0.02	[0.00; 0.06]	0.8%
Mullins et al., 2017	7	1123	0.01	[0.00; 0.01]	0.8%
Yagi et al., 2017	7	307	0.02	[0.01; 0.05]	0.8%
Jack et al., 2018	7	48	0.15	[0.06; 0.28]	0.7%
Grasso et al., 2018	2	100	0.02	[0.00; 0.07]	0.8%
Wang et al., 2018	1	26	0.04	[0.00; 0.20]	0.7%
Staartjes et al., 2018	17	551	0.03	[0.02; 0.05]	0.8%
Oni et al., 2018	2	85	0.02	[0.00; 0.08]	0.8%
Yan et al., 2019	39	82	0.48	[0.36; 0.59]	0.7%
Yang et al., 2019	4	49	0.08	[0.02; 0.20]	0.7%
Yang et al., 2019	2	58	0.03	[0.00; 0.12]	0.8%
Chang et al., 2019	3	50	0.06	[0.01; 0.17]	0.7%
Helseth et al., 2019	5	1300	0.00	[0.00; 0.01]	0.8%
Xu et al., 2019	1	40	0.02	[0.00; 0.13]	0.8%
Patel et al., 2019	0	172	0.00	[0.00; 0.02]	0.8%
Patel et al., 2019	0	100	0.00	[0.00; 0.04]	0.8%
Wewel et al., 2019	0	72	0.00	[0.00; 0.05]	0.8%
Grasso et al., 2019	0	35	0.00	[0.00; 0.10]	0.8%
Grasso et al., 2019	6	35	0.17	[0.07; 0.34]	0.6%
Aguilara et al., 2019	239	239	1.00	[0.98; 1.00]	0.8%

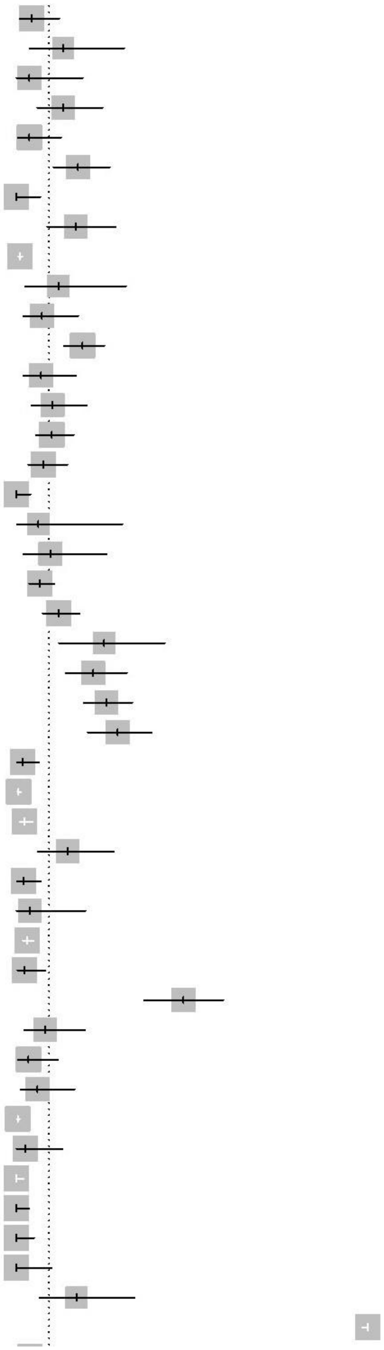

De Leo–Vargas et al., 2019	2	53	0.04	[0.00; 0.13]	0.8%
Opsenak et al., 2019	16	73	0.22	[0.13; 0.33]	0.7%
Zaki et al., 2019	11	389	0.03	[0.01; 0.05]	0.8%
Ahn et al., 2020	3	64	0.05	[0.01; 0.13]	0.8%
Narain et al., 2020	4	310	0.01	[0.00; 0.03]	0.8%
Guo et al., 2020	4	66	0.06	[0.02; 0.15]	0.8%
Houten et al., 2020	1	15	0.07	[0.00; 0.32]	0.6%
Huang et al., 2021	4	208	0.02	[0.01; 0.05]	0.8%
Sommaruga et al., 2021	7	166	0.04	[0.02; 0.08]	0.8%
Gowd et al., 2021	22	108	0.20	[0.13; 0.29]	0.7%
Fayed et al., 2021	63	321	0.20	[0.15; 0.24]	0.8%
Khalifeh et al., 2021	0	190	0.00	[0.00; 0.02]	0.8%
Nguyen et al., 2021	23	170	0.14	[0.09; 0.20]	0.8%
Wong et al., 2021	41	469	0.09	[0.06; 0.12]	0.8%
Niljianskul et al., 2021	2	31	0.06	[0.01; 0.21]	0.7%
Kaufman et al., 2022	27	131	0.21	[0.14; 0.29]	0.7%
Wei et al., 2022	27	163	0.17	[0.11; 0.23]	0.8%
Wei et al., 2022	11	171	0.06	[0.03; 0.11]	0.8%
Wei et al., 2022	26	182	0.14	[0.10; 0.20]	0.8%
Leng et al., 2022	3	73	0.04	[0.01; 0.12]	0.8%
Patel et al., 2022	3	161	0.02	[0.00; 0.05]	0.8%
Patel et al., 2022	4	161	0.02	[0.01; 0.06]	0.8%
Patel et al., 2022	13	320	0.04	[0.02; 0.07]	0.8%
Mu et al., 2022	7	77	0.09	[0.04; 0.18]	0.7%
Alsoof et al., 2022	129	792	0.16	[0.14; 0.19]	0.8%
Hao et al., 2022	5	302	0.02	[0.01; 0.04]	0.8%
Nayak et al., 2022	11	507	0.02	[0.01; 0.04]	0.8%
Kitamura et al., 2022	3	90	0.03	[0.01; 0.09]	0.8%
Guo et al., 2022	0	23	0.00	[0.00; 0.15]	0.8%
Guo et al., 2022	4	21	0.19	[0.05; 0.42]	0.5%
Niljianskul et al., 2023	10	85	0.12	[0.06; 0.21]	0.7%
Opsenak et al., 2023	13	133	0.10	[0.05; 0.16]	0.8%
Porche et al., 2023	105	250	0.42	[0.36; 0.48]	0.8%
Wang et al., 2023	4	56	0.07	[0.02; 0.17]	0.7%
Wang et al., 2023	0	50	0.00	[0.00; 0.07]	0.8%
Wang et al., 2023	0	44	0.00	[0.00; 0.08]	0.8%
Tang et al., 2023	0	31	0.00	[0.00; 0.11]	0.8%
Tang et al., 2023	3	36	0.08	[0.02; 0.22]	0.7%
Baig et al., 2023	1	65	0.02	[0.00; 0.08]	0.8%
Baig et al., 2023	2	65	0.03	[0.00; 0.11]	0.8%
Aguirre et al., 2023	5	55	0.09	[0.03; 0.20]	0.7%
Aguirre et al., 2023	19	69	0.28	[0.17; 0.40]	0.7%
Xiong et al., 2023	1	31	0.03	[0.00; 0.17]	0.8%
Xiong et al., 2023	1	21	0.05	[0.00; 0.24]	0.7%
Xiong et al., 2023	4	39	0.10	[0.03; 0.24]	0.7%

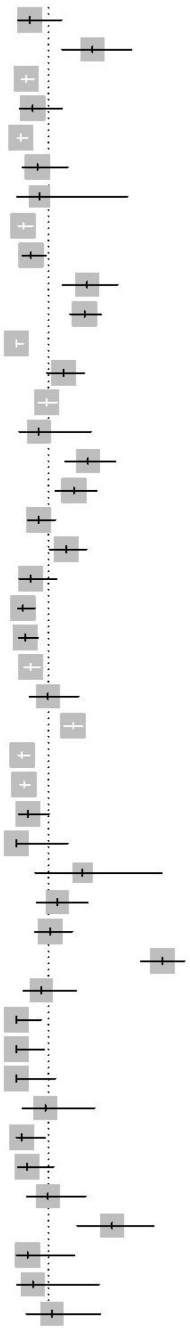

Tang et al., 2024	1	33	0.03	[0.00; 0.16]	0.8%
Zaho et al., 2024	0	13	0.00	[0.00; 0.25]	0.7%
Zaho et al., 2024	0	30	0.00	[0.00; 0.12]	0.8%
Paziuk et al., 2024	24	130	0.18	[0.12; 0.26]	0.7%
**Random effects model**		**22405**	**0.09**	**[0.07; 0.11]**	**100.0%**
Heterogeneity: $I^2 = 99\%$					

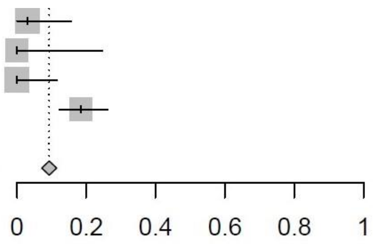

Supplementary Figure 3. Forest plot for pseudarthrosis.

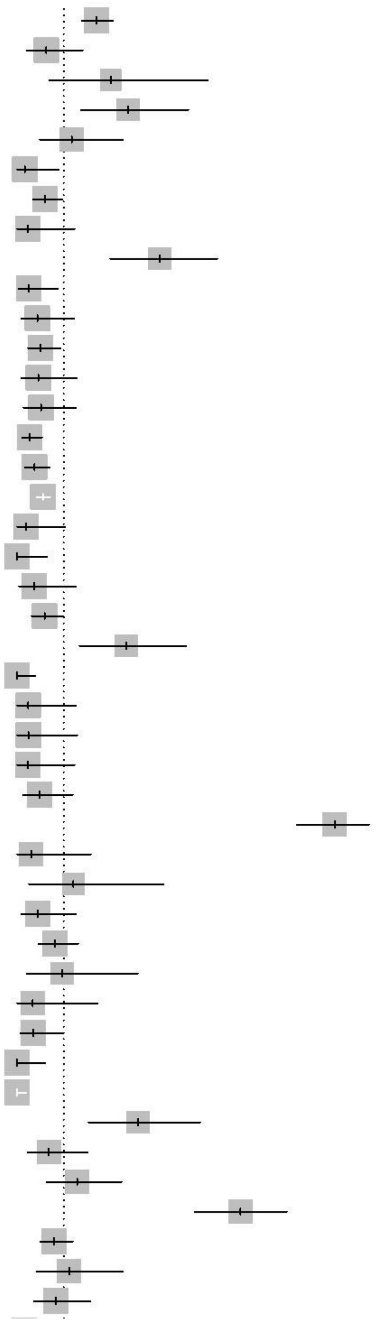

Study	Events	Total	Proportion	95%−CI	Weight
Cauthen et al., 1997	87	514	0.17	[0.14; 0.20]	1.0%
Wang et al., 1999	5	80	0.06	[0.02; 0.14]	1.0%
Wirth et al., 2000	5	25	0.20	[0.07; 0.41]	0.7%
Wang et al., 2000	14	59	0.24	[0.14; 0.37]	0.8%
Wang et al., 2000	7	60	0.12	[0.05; 0.23]	0.9%
Bruneau et al., 2001	1	60	0.02	[0.00; 0.09]	1.0%
Kaiser et al., 2002	15	251	0.06	[0.03; 0.10]	1.0%
Tureyen et al., 2003	1	43	0.02	[0.00; 0.12]	1.0%
Nirala et al., 2003	21	69	0.30	[0.20; 0.43]	0.8%
Samartzis et al., 2003	2	80	0.02	[0.00; 0.09]	1.0%
Samartzis et al., 2004	3	69	0.04	[0.01; 0.12]	1.0%
Cho et al., 2004	9	180	0.05	[0.02; 0.09]	1.0%
Samartzis et al., 2005	3	66	0.05	[0.01; 0.13]	1.0%
Hwang et al., 2005	4	78	0.05	[0.01; 0.13]	1.0%
Mummaneni et al., 2007	7	265	0.03	[0.01; 0.05]	1.0%
Mobbs et al., 2007	9	242	0.04	[0.02; 0.07]	1.0%
Fountas et al., 2007	56	1015	0.06	[0.04; 0.07]	1.0%
Aryan et al., 2007	1	52	0.02	[0.00; 0.10]	1.0%
Chang−Jung et al., 2008	0	56	0.00	[0.00; 0.06]	1.0%
Ghahreman et al., 2009	2	55	0.04	[0.00; 0.13]	1.0%
Heller et al., 2009	13	221	0.06	[0.03; 0.10]	1.0%
Kast et al., 2009	14	60	0.23	[0.13; 0.36]	0.8%
Chen et al., 2009	0	92	0.00	[0.00; 0.04]	1.0%
Uribe et al., 2009	1	42	0.02	[0.00; 0.13]	1.0%
Coric et al., 2010	1	41	0.02	[0.00; 0.13]	1.0%
Guo et al., 2010	1	43	0.02	[0.00; 0.12]	1.0%
Song et al., 2010	4	83	0.05	[0.01; 0.12]	1.0%
Sheperd et al., 2012	103	152	0.68	[0.60; 0.75]	0.9%
Coric et al., 2012	1	33	0.03	[0.00; 0.16]	0.9%
Song et al., 2012	3	25	0.12	[0.03; 0.31]	0.8%
Wu et al., 2012	3	67	0.04	[0.01; 0.13]	1.0%
Phillips et al., 2013	15	185	0.08	[0.05; 0.13]	1.0%
Park et al., 2013	3	31	0.10	[0.02; 0.26]	0.8%
Landriel et al., 2013	1	30	0.03	[0.00; 0.17]	0.9%
Tracey et al., 2014	3	88	0.03	[0.01; 0.10]	1.0%
Kasliwal et al., 2014	0	59	0.00	[0.00; 0.06]	1.0%
Kao et al., 2014	0	182	0.00	[0.00; 0.02]	1.0%
Yoo et al., 2014	15	58	0.26	[0.15; 0.39]	0.8%
Omidi−Kashani et al., 2014	5	74	0.07	[0.02; 0.15]	0.9%
Lee et al., 2014	10	78	0.13	[0.06; 0.22]	0.9%
Klingler et al., 2014	51	107	0.48	[0.38; 0.58]	0.9%
Shiban et al., 2015	21	265	0.08	[0.05; 0.12]	1.0%
Chen et al., 2016	6	54	0.11	[0.04; 0.23]	0.9%
De La Garza−Ramos et al., 2016	8	97	0.08	[0.04; 0.16]	1.0%

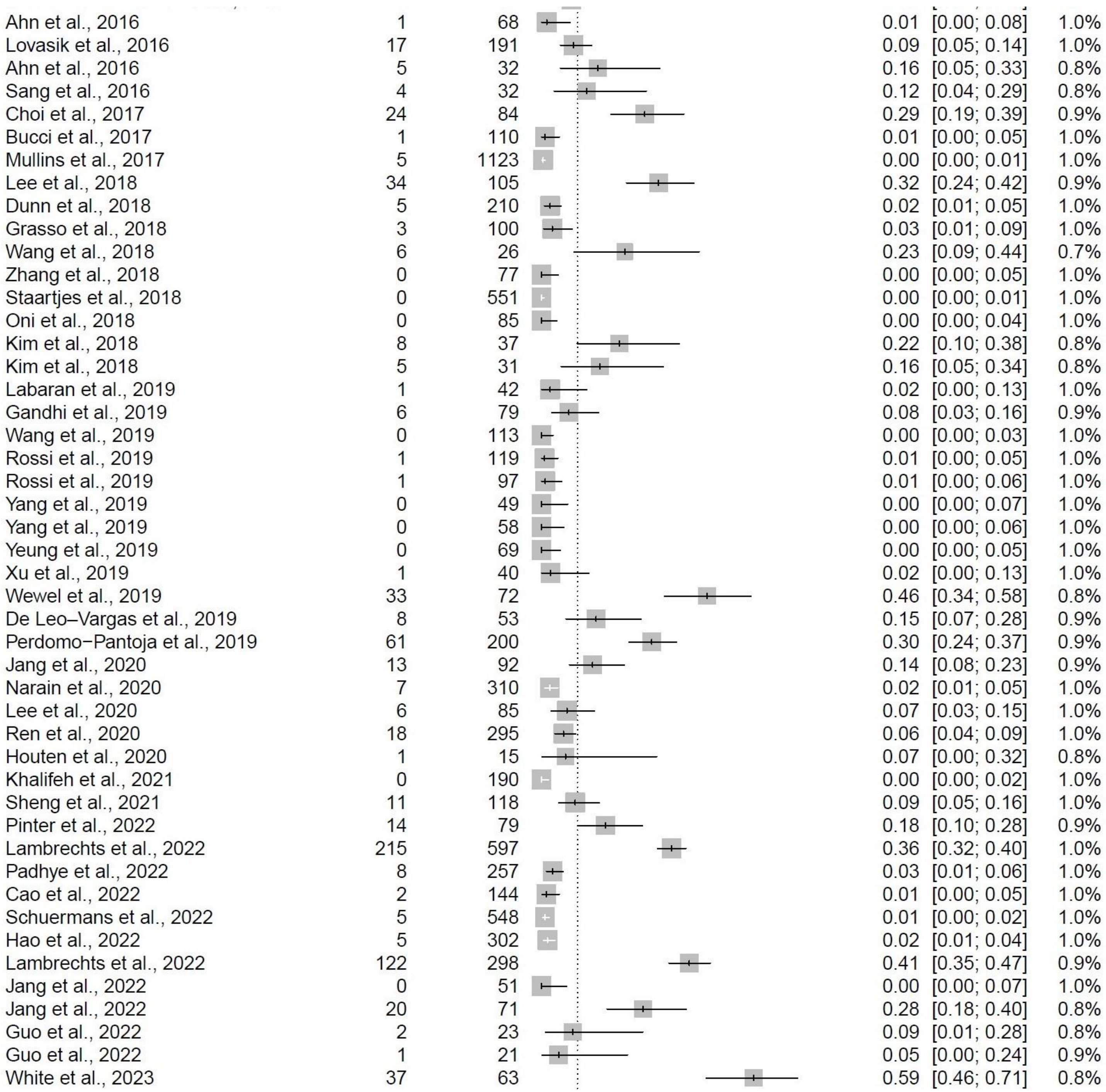
Ahn et al., 2016 1 68 0.01 [0.00; 0.08] 1.0%
Lovasik et al., 2016 17 191 0.09 [0.05; 0.14] 1.0%
Ahn et al., 2016 5 32 0.16 [0.05; 0.33] 0.8%
Sang et al., 2016 4 32 0.12 [0.04; 0.29] 0.8%
Choi et al., 2017 24 84 0.29 [0.19; 0.39] 0.9%
Bucci et al., 2017 1 110 0.01 [0.00; 0.05] 1.0%
Mullins et al., 2017 5 1123 0.00 [0.00; 0.01] 1.0%
Lee et al., 2018 34 105 0.32 [0.24; 0.42] 0.9%
Dunn et al., 2018 5 210 0.02 [0.01; 0.05] 1.0%
Grasso et al., 2018 3 100 0.03 [0.01; 0.09] 1.0%
Wang et al., 2018 6 26 0.23 [0.09; 0.44] 0.7%
Zhang et al., 2018 0 77 0.00 [0.00; 0.05] 1.0%
Staartjes et al., 2018 0 551 0.00 [0.00; 0.01] 1.0%
Oni et al., 2018 0 85 0.00 [0.00; 0.04] 1.0%
Kim et al., 2018 8 37 0.22 [0.10; 0.38] 0.8%
Kim et al., 2018 5 31 0.16 [0.05; 0.34] 0.8%
Labaran et al., 2019 1 42 0.02 [0.00; 0.13] 1.0%
Gandhi et al., 2019 6 79 0.08 [0.03; 0.16] 0.9%
Wang et al., 2019 0 113 0.00 [0.00; 0.03] 1.0%
Rossi et al., 2019 1 119 0.01 [0.00; 0.05] 1.0%
Rossi et al., 2019 1 97 0.01 [0.00; 0.06] 1.0%
Yang et al., 2019 0 49 0.00 [0.00; 0.07] 1.0%
Yang et al., 2019 0 58 0.00 [0.00; 0.06] 1.0%
Yeung et al., 2019 0 69 0.00 [0.00; 0.05] 1.0%
Xu et al., 2019 1 40 0.02 [0.00; 0.13] 1.0%
Wewel et al., 2019 33 72 0.46 [0.34; 0.58] 0.8%
De Leo−Vargas et al., 2019 8 53 0.15 [0.07; 0.28] 0.9%
Perdomo−Pantoja et al., 2019 61 200 0.30 [0.24; 0.37] 0.9%
Jang et al., 2020 13 92 0.14 [0.08; 0.23] 0.9%
Narain et al., 2020 7 310 0.02 [0.01; 0.05] 1.0%
Lee et al., 2020 6 85 0.07 [0.03; 0.15] 1.0%
Ren et al., 2020 18 295 0.06 [0.04; 0.09] 1.0%
Houten et al., 2020 1 15 0.07 [0.00; 0.32] 0.8%
Khalifeh et al., 2021 0 190 0.00 [0.00; 0.02] 1.0%
Sheng et al., 2021 11 118 0.09 [0.05; 0.16] 1.0%
Pinter et al., 2022 14 79 0.18 [0.10; 0.28] 0.9%
Lambrechts et al., 2022 215 597 0.36 [0.32; 0.40] 1.0%
Padhye et al., 2022 8 257 0.03 [0.01; 0.06] 1.0%
Cao et al., 2022 2 144 0.01 [0.00; 0.05] 1.0%
Schuermans et al., 2022 5 548 0.01 [0.00; 0.02] 1.0%
Hao et al., 2022 5 302 0.02 [0.01; 0.04] 1.0%
Lambrechts et al., 2022 122 298 0.41 [0.35; 0.47] 0.9%
Jang et al., 2022 0 51 0.00 [0.00; 0.07] 1.0%
Jang et al., 2022 20 71 0.28 [0.18; 0.40] 0.8%
Guo et al., 2022 2 23 0.09 [0.01; 0.28] 0.8%
Guo et al., 2022 1 21 0.05 [0.00; 0.24] 0.9%
White et al., 2023 37 63 0.59 [0.46; 0.71] 0.8%

Wang et al., 2023	6	56	0.11	[0.04; 0.22]	0.9%
Wang et al., 2023	6	50	0.12	[0.05; 0.24]	0.9%
Wang et al., 2023	5	44	0.11	[0.04; 0.25]	0.9%
Tang et al., 2023	2	31	0.06	[0.01; 0.21]	0.9%
Tang et al., 2023	1	36	0.03	[0.00; 0.15]	1.0%
Chanbour et al., 2023	141	406	0.35	[0.30; 0.40]	1.0%
Zhuo et al., 2023	44	164	0.27	[0.20; 0.34]	0.9%
Baig et al., 2023	10	65	0.15	[0.08; 0.26]	0.9%
Baig et al., 2023	7	65	0.11	[0.04; 0.21]	0.9%
Aguirre et al., 2023	1	55	0.02	[0.00; 0.10]	1.0%
Aguirre et al., 2023	8	69	0.12	[0.05; 0.22]	0.9%
Xiong et al., 2023	2	31	0.06	[0.01; 0.21]	0.9%
Xiong et al., 2023	2	21	0.10	[0.01; 0.30]	0.8%
Xiong et al., 2023	2	39	0.05	[0.01; 0.17]	0.9%
Tang et al., 2024	0	33	0.00	[0.00; 0.11]	1.0%
Zaho et al., 2024	1	13	0.08	[0.00; 0.36]	0.7%
Zaho et al., 2024	3	30	0.10	[0.02; 0.27]	0.8%
**Random effects model**		**14106**	**0.10**	**[0.08; 0.12]**	**100.0%**

Heterogeneity: I^2 = 99%

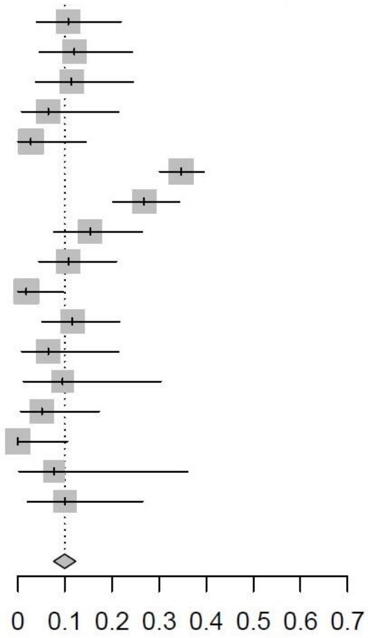

Supplementary Figure 4. Forest plot for wound dehiscence.

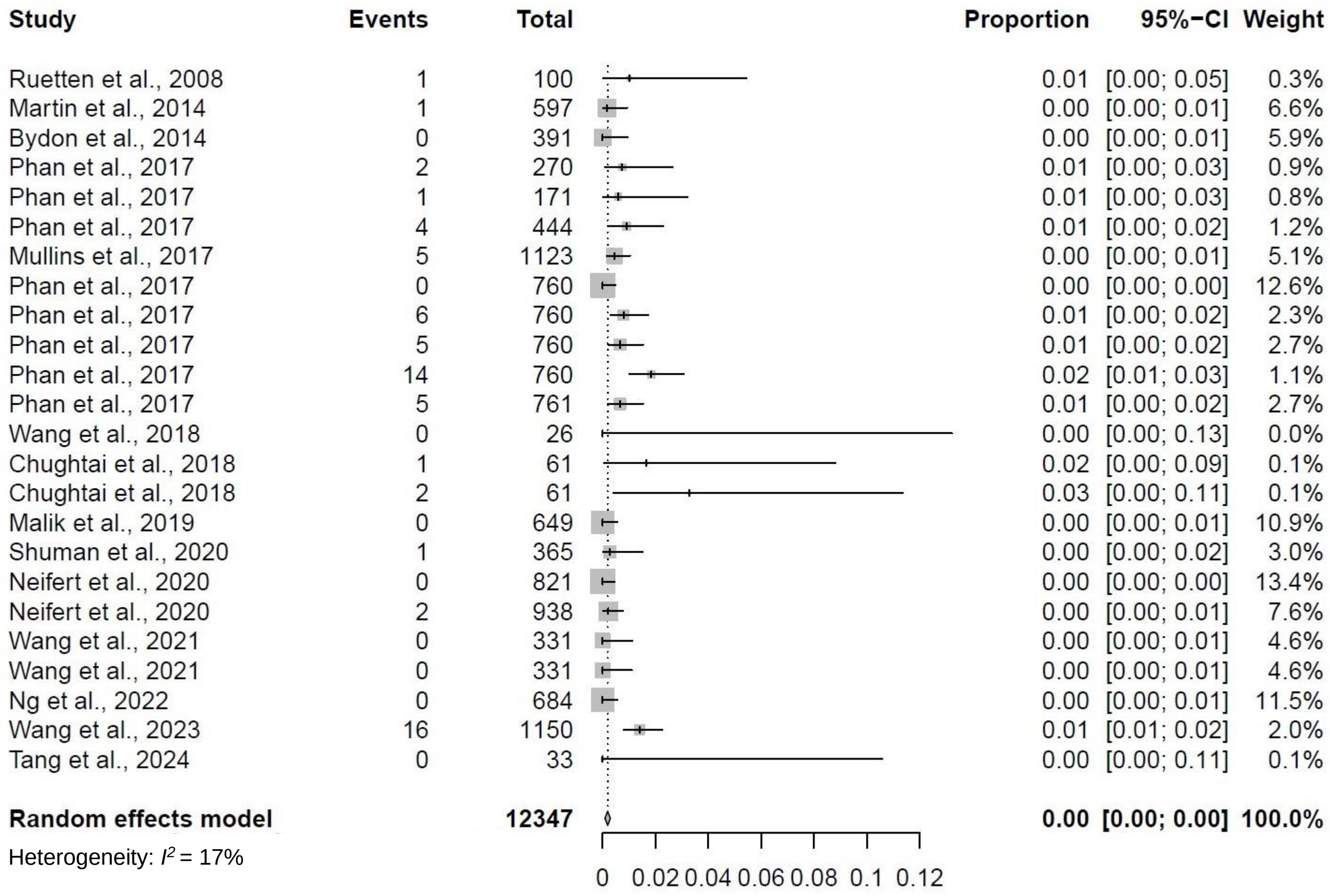

Supplementary Figure 5. Forest plot for general surgical site infection.

Study	Events	Total	Proportion	95%−CI	Weight
Nirala et al., 2003	2	69	0.03	[0.00; 0.10]	0.2%
Samartzis et al., 2004	1	69	0.01	[0.00; 0.08]	0.3%
Hwang et al., 2005	0	78	0.00	[0.00; 0.05]	0.7%
Korinth et al., 2006	0	124	0.00	[0.00; 0.03]	1.4%
Lied et al., 2006	4	390	0.01	[0.00; 0.03]	1.6%
Mobbs et al., 2007	1	242	0.00	[0.00; 0.02]	2.0%
Ruetten et al., 2008	0	100	0.00	[0.00; 0.04]	1.0%
Kast et al., 2009	0	60	0.00	[0.00; 0.06]	0.5%
Chen et al., 2009	0	92	0.00	[0.00; 0.04]	0.9%
Uribe et al., 2009	0	42	0.00	[0.00; 0.08]	0.2%
Coric et al., 2010	0	41	0.00	[0.00; 0.09]	0.2%
Joseffer et al., 2010	8	390	0.02	[0.01; 0.04]	1.0%
Coric et al., 2011	6	133	0.05	[0.02; 0.10]	0.2%
Iampreechakul et al., 2011	2	67	0.03	[0.00; 0.10]	0.2%
Coric et al., 2012	0	33	0.00	[0.00; 0.11]	0.2%
Liu et al., 2012	0	69	0.00	[0.00; 0.05]	0.6%
Lied et al., 2012	0	96	0.00	[0.00; 0.04]	1.0%
Wang et al., 2014	0	63	0.00	[0.00; 0.06]	0.5%
Martin et al., 2014	3	597	0.01	[0.00; 0.01]	2.6%
Tracey et al., 2014	0	88	0.00	[0.00; 0.04]	0.9%
Bydon et al., 2014	2	391	0.01	[0.00; 0.02]	2.2%
Omidi−Kashani et al., 2014	5	74	0.07	[0.02; 0.15]	0.1%
Nanda et al., 2014	3	1576	0.00	[0.00; 0.01]	3.6%
Elsamadicy et al., 2015	0	28	0.00	[0.00; 0.12]	0.1%
Elsamadicy et al., 2015	1	32	0.03	[0.00; 0.16]	0.1%
Liu et al., 2015	2	22	0.09	[0.01; 0.29]	0.0%
Chen et al., 2016	0	54	0.00	[0.00; 0.07]	0.4%
De La Garza−Ramos et al., 2016	2	97	0.02	[0.00; 0.07]	0.3%
Wang et al., 2016	0	52	0.00	[0.00; 0.07]	0.4%
Shi et al., 2016	0	65	0.00	[0.00; 0.06]	0.5%
Adamson et al., 2016	2	1478	0.00	[0.00; 0.00]	3.6%
Ahn et al., 2016	0	68	0.00	[0.00; 0.05]	0.6%
Radcliff et al., 2016	0	25	0.00	[0.00; 0.14]	0.1%
Adogwa et al., 2016	2	59	0.03	[0.00; 0.12]	0.1%
Adogwa et al., 2016	1	81	0.01	[0.00; 0.07]	0.4%
ElAbed et al., 2016	1	90	0.01	[0.00; 0.06]	0.5%
Li et al., 2017	0	31	0.00	[0.00; 0.11]	0.1%
Phan et al., 2017	2	444	0.00	[0.00; 0.02]	2.5%
Mullins et al., 2017	11	1123	0.01	[0.00; 0.02]	2.6%
Phan et al., 2017	1	760	0.00	[0.00; 0.01]	3.5%
Phan et al., 2017	0	760	0.00	[0.00; 0.00]	3.6%
Phan et al., 2017	0	760	0.00	[0.00; 0.00]	3.6%
Phan et al., 2017	4	760	0.01	[0.00; 0.01]	2.8%
Phan et al., 2017	0	761	0.00	[0.00; 0.00]	3.6%

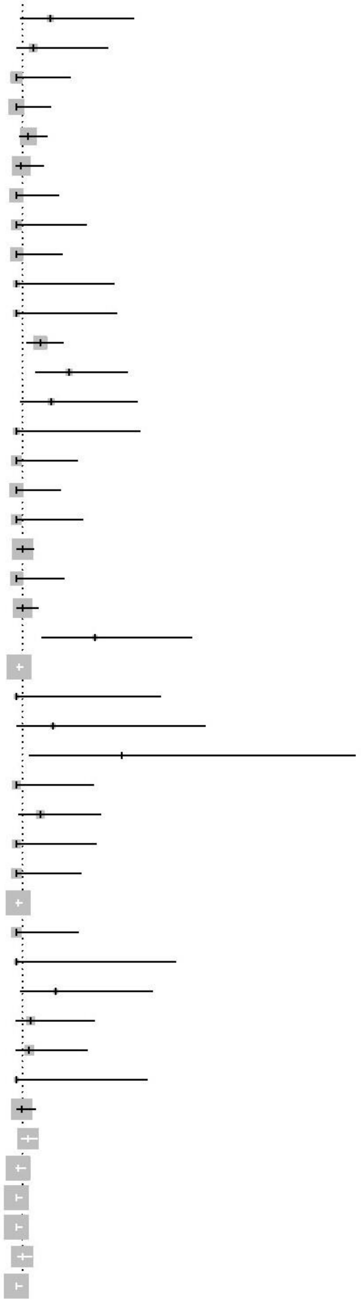

Yagi et al., 2017	2	307		0.01	[0.00; 0.02]	1.8%
Dunn et al., 2018	1	210		0.00	[0.00; 0.03]	1.7%
Wang et al., 2018	0	26		0.00	[0.00; 0.13]	0.1%
Staartjes et al., 2018	11	551		0.02	[0.01; 0.04]	1.3%
Chughtai et al., 2018	4	61		0.07	[0.02; 0.16]	0.1%
Chughtai et al., 2018	11	61		0.18	[0.09; 0.30]	0.0%
Bivo et al., 2019	1	96		0.01	[0.00; 0.06]	0.6%
Lin et al., 2019	1	55		0.02	[0.00; 0.10]	0.2%
Rossi et al., 2019	1	119		0.01	[0.00; 0.05]	0.8%
Rossi et al., 2019	1	97		0.01	[0.00; 0.06]	0.6%
Helseth et al., 2019	1	1300		0.00	[0.00; 0.00]	3.7%
Malik et al., 2019	5	649		0.01	[0.00; 0.02]	2.3%
Zaki et al., 2019	8	389		0.02	[0.01; 0.04]	1.0%
Jang et al., 2020	0	92		0.00	[0.00; 0.04]	0.9%
Shuman et al., 2020	1	365		0.00	[0.00; 0.02]	2.7%
Ahn et al., 2020	0	64		0.00	[0.00; 0.06]	0.5%
Narain et al., 2020	0	310		0.00	[0.00; 0.01]	3.0%
Labaran et al., 2020	32	992		0.03	[0.02; 0.05]	1.4%
Neifert et al., 2020	5	821		0.01	[0.00; 0.01]	2.7%
Neifert et al., 2020	4	938		0.00	[0.00; 0.01]	3.0%
Houten et al., 2020	0	15		0.00	[0.00; 0.22]	0.0%
Yang et al., 2021	1	32		0.03	[0.00; 0.16]	0.1%
Yang et al., 2021	0	56		0.00	[0.00; 0.06]	0.4%
Fayed et al., 2021	1	321		0.00	[0.00; 0.02]	2.5%
Khalifeh et al., 2021	0	190		0.00	[0.00; 0.02]	2.2%
Wang et al., 2021	7	331		0.02	[0.01; 0.04]	0.9%
Wang et al., 2021	3	331		0.01	[0.00; 0.03]	1.5%
Ma et al., 2022	4	68		0.06	[0.02; 0.14]	0.1%
Ng et al., 2022	1	684		0.00	[0.00; 0.01]	3.4%
Alsoof et al., 2022	13	792		0.02	[0.01; 0.03]	1.8%
Nayak et al., 2022	7	507		0.01	[0.01; 0.03]	1.5%
Kitamura et al., 2022	4	90		0.04	[0.01; 0.11]	0.1%
Wang et al., 2023	1	1150		0.00	[0.00; 0.00]	3.6%
Wang et al., 2023	0	56		0.00	[0.00; 0.06]	0.4%
Wang et al., 2023	0	50		0.00	[0.00; 0.07]	0.3%
Wang et al., 2023	0	44		0.00	[0.00; 0.08]	0.3%
Tang et al., 2023	0	31		0.00	[0.00; 0.11]	0.1%
Tang et al., 2023	0	36		0.00	[0.00; 0.10]	0.2%
Aguirre et al., 2023	0	55		0.00	[0.00; 0.06]	0.4%
Aguirre et al., 2023	2	69		0.03	[0.00; 0.10]	0.2%
Tang et al., 2024	0	33		0.00	[0.00; 0.11]	0.2%
**Random effects model**		**24878**		**0.01**	**[0.00; 0.01]**	**100.0%**

Heterogeneity: I^2 = 67%

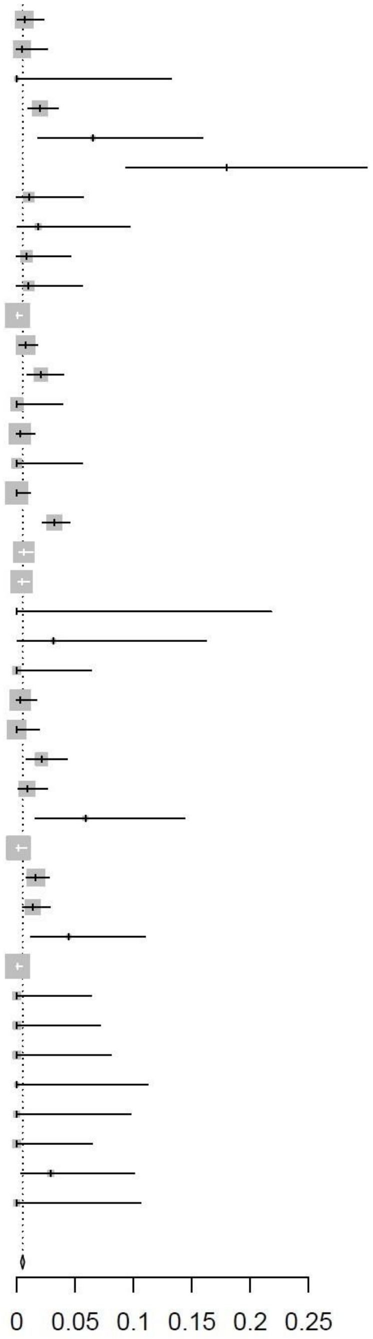

Supplementary Figure 6. Forest plot for superficial surgical site infection.

Study	Events	Total	Proportion	95%−CI	Weight
Nirala et al., 2003	2	69	0.03	[0.00; 0.10]	0.3%
Samartzis et al., 2004	1	69	0.01	[0.00; 0.08]	0.6%
Hwang et al., 2005	0	78	0.00	[0.00; 0.05]	1.3%
Korinth et al., 2006	0	124	0.00	[0.00; 0.03]	2.6%
Mobbs et al., 2007	1	242	0.00	[0.00; 0.02]	3.6%
Kast et al., 2009	0	60	0.00	[0.00; 0.06]	0.9%
Uribe et al., 2009	0	42	0.00	[0.00; 0.08]	0.5%
Coric et al., 2010	0	41	0.00	[0.00; 0.09]	0.4%
Joseffer et al., 2010	8	390	0.02	[0.01; 0.04]	1.8%
Coric et al., 2011	6	133	0.05	[0.02; 0.10]	0.4%
Iampreechakul et al., 2011	2	67	0.03	[0.00; 0.10]	0.3%
Coric et al., 2012	0	33	0.00	[0.00; 0.11]	0.3%
Liu et al., 2012	0	69	0.00	[0.00; 0.05]	1.1%
Lied et al., 2012	0	96	0.00	[0.00; 0.04]	1.8%
Wang et al., 2014	0	63	0.00	[0.00; 0.06]	0.9%
Martin et al., 2014	1	597	0.00	[0.00; 0.01]	6.0%
Tracey et al., 2014	0	88	0.00	[0.00; 0.04]	1.6%
Omidi−Kashani et al., 2014	5	74	0.07	[0.02; 0.15]	0.2%
Elsamadicy et al., 2015	0	28	0.00	[0.00; 0.12]	0.2%
Elsamadicy et al., 2015	1	32	0.03	[0.00; 0.16]	0.1%
Liu et al., 2015	1	22	0.05	[0.00; 0.23]	0.1%
Chen et al., 2016	0	54	0.00	[0.00; 0.07]	0.7%
Wang et al., 2016	0	52	0.00	[0.00; 0.07]	0.7%
Shi et al., 2016	0	65	0.00	[0.00; 0.06]	1.0%
Ahn et al., 2016	0	68	0.00	[0.00; 0.05]	1.1%
Radcliff et al., 2016	0	25	0.00	[0.00; 0.14]	0.2%
Adogwa et al., 2016	2	59	0.03	[0.00; 0.12]	0.2%
Adogwa et al., 2016	1	81	0.01	[0.00; 0.07]	0.8%
Dunn et al., 2018	1	210	0.00	[0.00; 0.03]	3.1%
Wang et al., 2018	0	26	0.00	[0.00; 0.13]	0.2%
Chughtai et al., 2018	4	61	0.07	[0.02; 0.16]	0.1%
Chughtai et al., 2018	7	61	0.11	[0.05; 0.22]	0.1%
Lin et al., 2019	1	55	0.02	[0.00; 0.10]	0.4%
Rossi et al., 2019	1	119	0.01	[0.00; 0.05]	1.5%
Rossi et al., 2019	1	97	0.01	[0.00; 0.06]	1.1%
Malik et al., 2019	5	649	0.01	[0.00; 0.02]	4.2%
Jang et al., 2020	0	92	0.00	[0.00; 0.04]	1.7%
Shuman et al., 2020	1	365	0.00	[0.00; 0.02]	4.9%
Ahn et al., 2020	0	64	0.00	[0.00; 0.06]	1.0%
Narain et al., 2020	0	310	0.00	[0.00; 0.01]	5.4%
Labaran et al., 2020	32	992	0.03	[0.02; 0.05]	2.6%
Neifert et al., 2020	2	821	0.00	[0.00; 0.01]	5.9%
Neifert et al., 2020	1	938	0.00	[0.00; 0.01]	6.4%

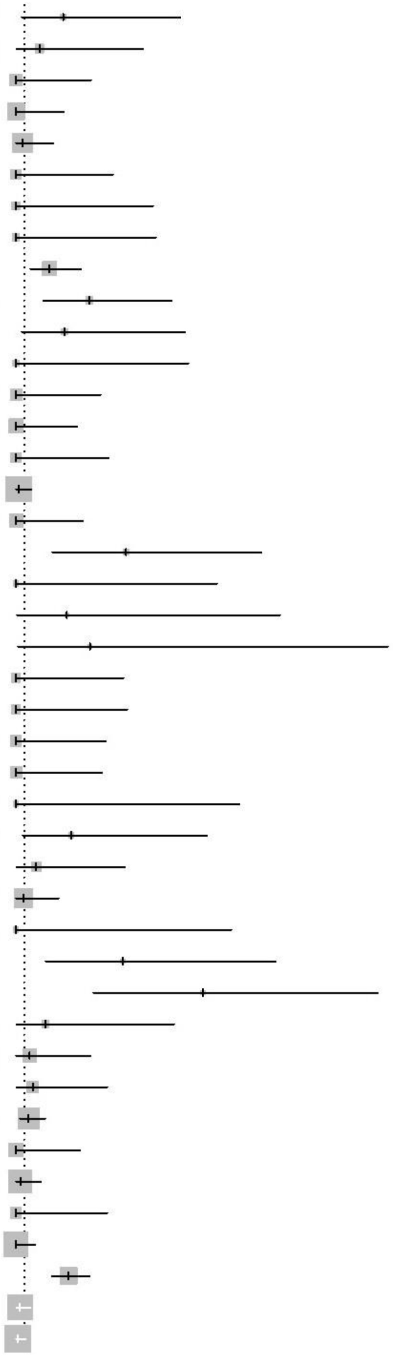

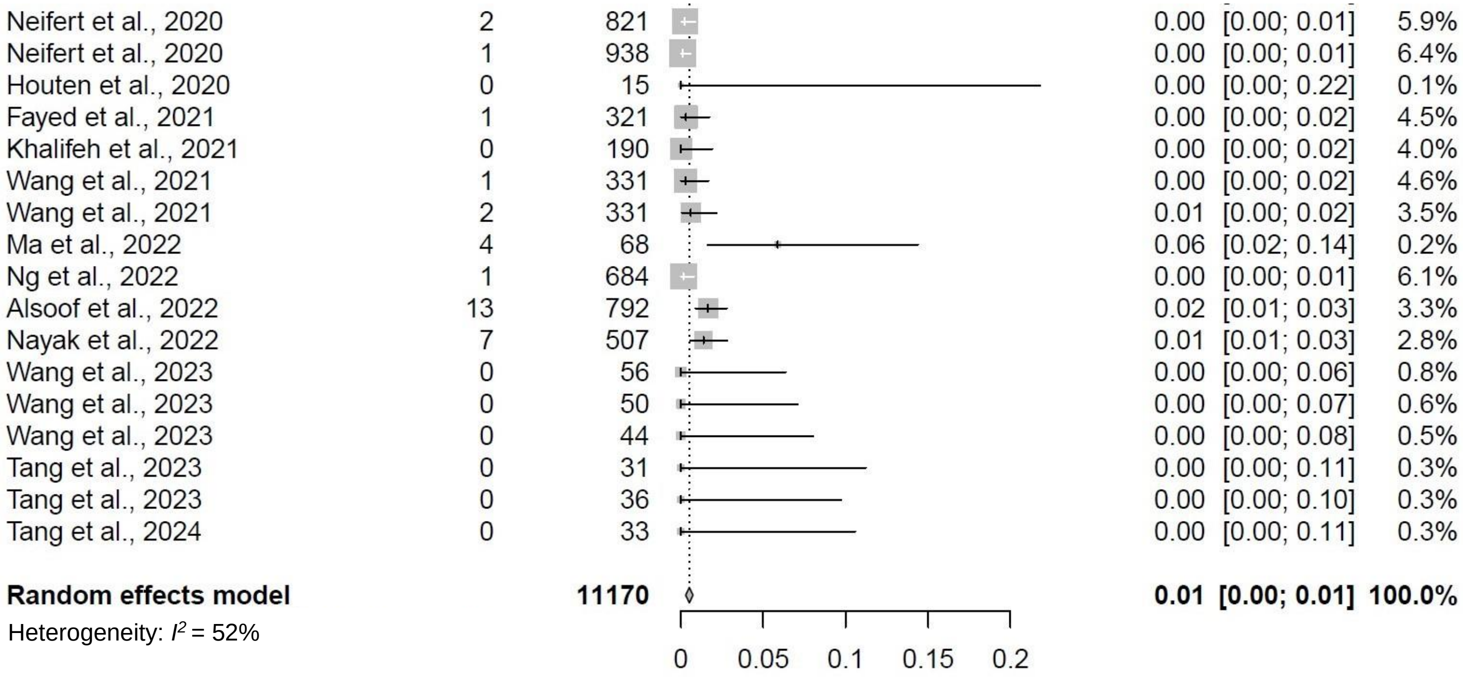
Neifert et al., 2020 2 821 0.00 [0.00; 0.01] 5.9%
Neifert et al., 2020 1 938 0.00 [0.00; 0.01] 6.4%
Houten et al., 2020 0 15 0.00 [0.00; 0.22] 0.1%
Fayed et al., 2021 1 321 0.00 [0.00; 0.02] 4.5%
Khalifeh et al., 2021 0 190 0.00 [0.00; 0.02] 4.0%
Wang et al., 2021 1 331 0.00 [0.00; 0.02] 4.6%
Wang et al., 2021 2 331 0.01 [0.00; 0.02] 3.5%
Ma et al., 2022 4 68 0.06 [0.02; 0.14] 0.2%
Ng et al., 2022 1 684 0.00 [0.00; 0.01] 6.1%
Alsoof et al., 2022 13 792 0.02 [0.01; 0.03] 3.3%
Nayak et al., 2022 7 507 0.01 [0.01; 0.03] 2.8%
Wang et al., 2023 0 56 0.00 [0.00; 0.06] 0.8%
Wang et al., 2023 0 50 0.00 [0.00; 0.07] 0.6%
Wang et al., 2023 0 44 0.00 [0.00; 0.08] 0.5%
Tang et al., 2023 0 31 0.00 [0.00; 0.11] 0.3%
Tang et al., 2023 0 36 0.00 [0.00; 0.10] 0.3%
Tang et al., 2024 0 33 0.00 [0.00; 0.11] 0.3%
Random effects model 11170 0.01 [0.00; 0.01] 100.0%
Heterogeneity: I2 = 52%
0 0.05 0.1 0.15 0.2

Supplementary Figure 7. Forest plot for deep surgical site infection/abscess.

Study	Events	Total	Proportion	95%−CI	Weight
Kast et al., 2009	0	60	0.00	[0.00; 0.06]	0.1%
Chen et al., 2009	0	92	0.00	[0.00; 0.04]	0.3%
Coric et al., 2010	0	41	0.00	[0.00; 0.09]	0.1%
Coric et al., 2012	0	33	0.00	[0.00; 0.11]	0.0%
Liu et al., 2012	0	69	0.00	[0.00; 0.05]	0.2%
Wang et al., 2014	0	63	0.00	[0.00; 0.06]	0.2%
Martin et al., 2014	2	597	0.00	[0.00; 0.01]	3.5%
Liu et al., 2015	1	22	0.05	[0.00; 0.23]	0.0%
Chen et al., 2016	0	54	0.00	[0.00; 0.07]	0.1%
De La Garza−Ramos et al., 2016	2	97	0.02	[0.00; 0.07]	0.1%
Wang et al., 2016	0	52	0.00	[0.00; 0.07]	0.1%
Shi et al., 2016	0	65	0.00	[0.00; 0.06]	0.2%
Ahn et al., 2016	0	68	0.00	[0.00; 0.05]	0.2%
Radcliff et al., 2016	0	25	0.00	[0.00; 0.14]	0.0%
ElAbed et al., 2016	1	90	0.01	[0.00; 0.06]	0.2%
Yagi et al., 2017	0	307	0.00	[0.00; 0.01]	3.7%
Wang et al., 2018	0	26	0.00	[0.00; 0.13]	0.0%
Helseth et al., 2019	1	1300	0.00	[0.00; 0.00]	32.9%
Malik et al., 2019	0	649	0.00	[0.00; 0.01]	16.4%
Jang et al., 2020	0	92	0.00	[0.00; 0.04]	0.3%
Ahn et al., 2020	0	64	0.00	[0.00; 0.06]	0.2%
Narain et al., 2020	0	310	0.00	[0.00; 0.01]	3.8%
Neifert et al., 2020	3	821	0.00	[0.00; 0.01]	4.4%
Neifert et al., 2020	3	938	0.00	[0.00; 0.01]	5.7%
Wang et al., 2021	0	331	0.00	[0.00; 0.01]	4.3%
Wang et al., 2021	0	331	0.00	[0.00; 0.01]	4.3%
Ng et al., 2022	0	684	0.00	[0.00; 0.01]	18.3%
Wang et al., 2023	0	56	0.00	[0.00; 0.06]	0.1%
Wang et al., 2023	0	50	0.00	[0.00; 0.07]	0.1%
Wang et al., 2023	0	44	0.00	[0.00; 0.08]	0.1%
Tang et al., 2023	0	31	0.00	[0.00; 0.11]	0.0%
Tang et al., 2023	0	36	0.00	[0.00; 0.10]	0.1%
Tang et al., 2024	0	33	0.00	[0.00; 0.11]	0.0%
**Random effects model**		**7531**	**0.00**	**[0.00; 0.00]**	**100.0%**

Heterogeneity: $I^2 = 0\%$

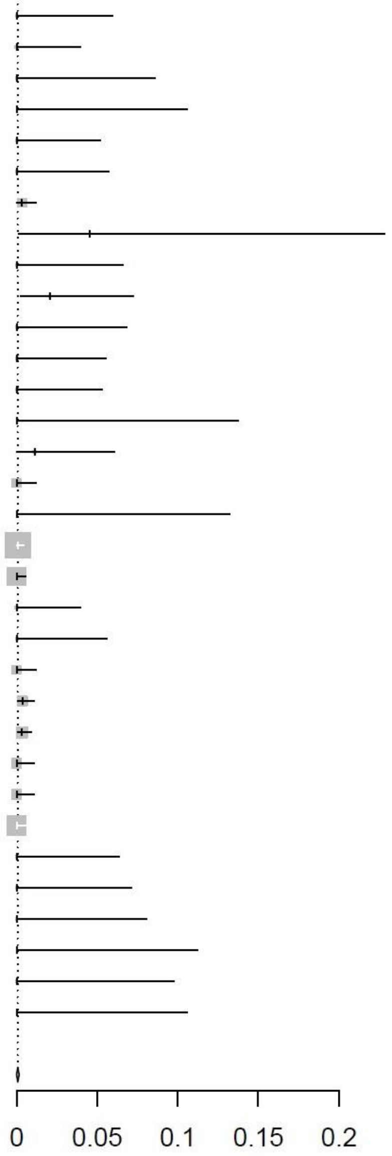

Supplementary Figure 8. Forest plot for wound hematoma/seroma.

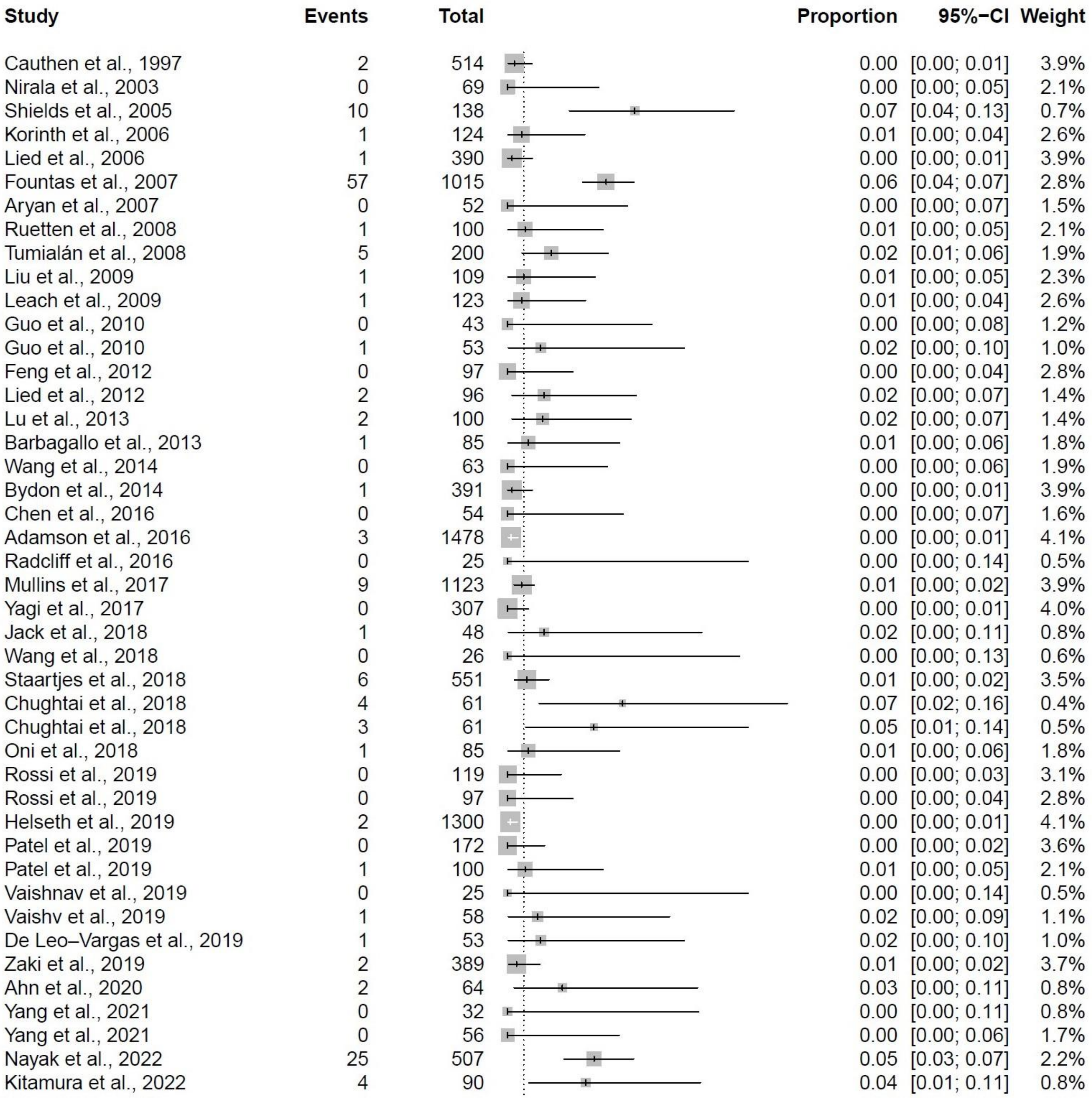

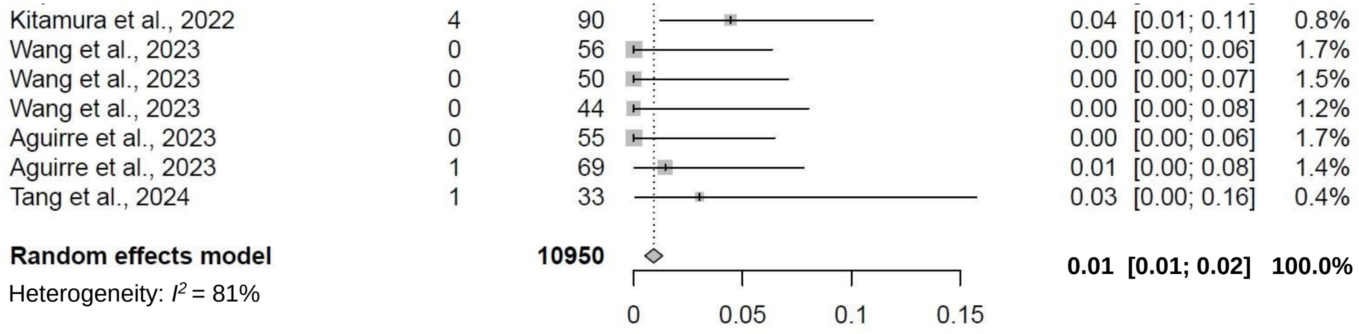
Kitamura et al., 2022 4 90 0.04 [0.01; 0.11] 0.8%
Wang et al., 2023 0 56 0.00 [0.00; 0.06] 1.7%
Wang et al., 2023 0 50 0.00 [0.00; 0.07] 1.5%
Wang et al., 2023 0 44 0.00 [0.00; 0.08] 1.2%
Aguirre et al., 2023 0 55 0.00 [0.00; 0.06] 1.7%
Aguirre et al., 2023 1 69 0.01 [0.00; 0.08] 1.4%
Tang et al., 2024 1 33 0.03 [0.00; 0.16] 0.4%
Random effects model 10950 0.01 [0.01; 0.02] 100.0%
Heterogeneity: I2 = 81%
0
0.05
0.1
0.15

Supplementary Figure 9. Forest plot for epidural hematoma.

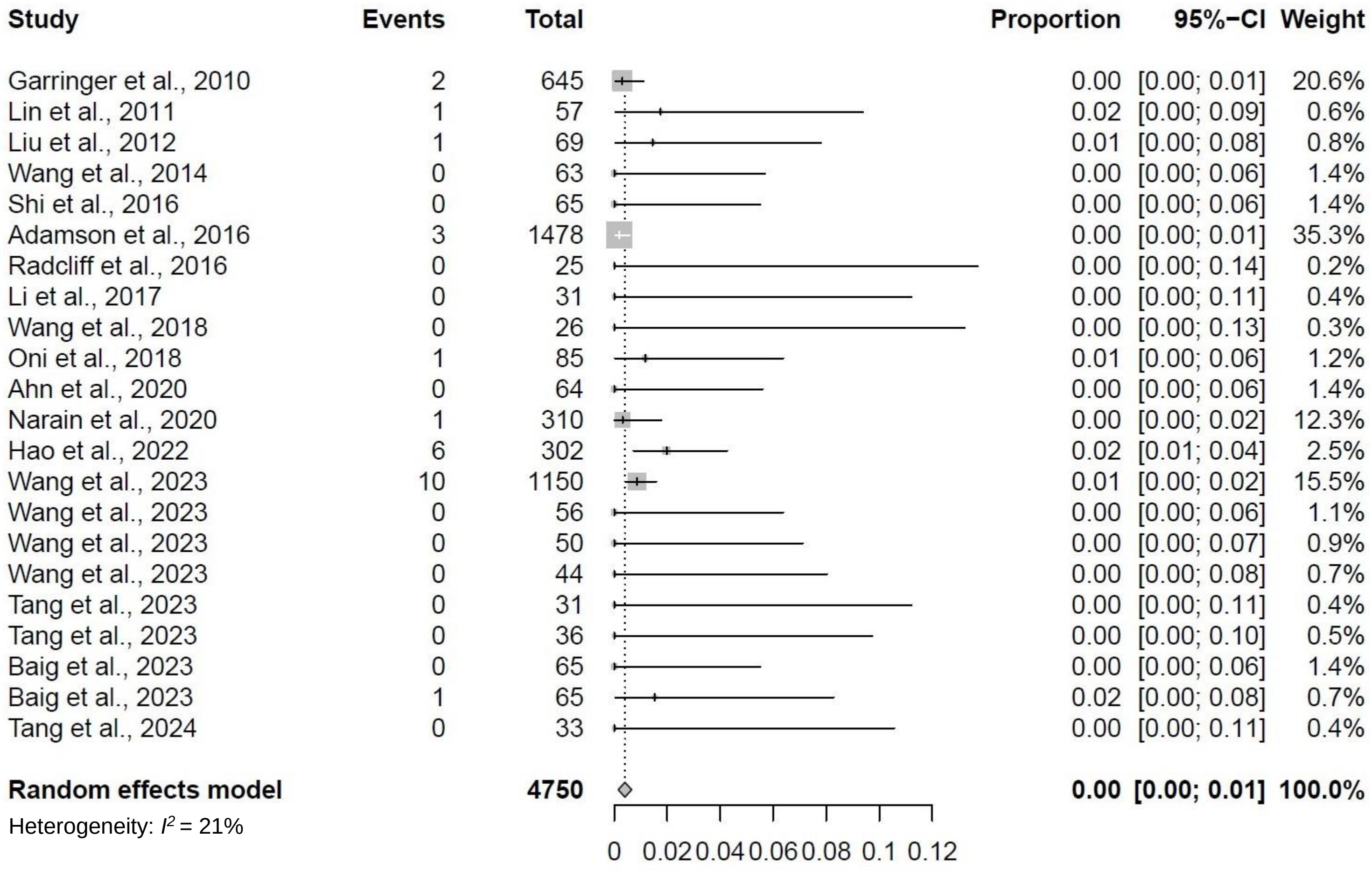

Supplementary Figure 10. Forest plot for Horner's syndrome.

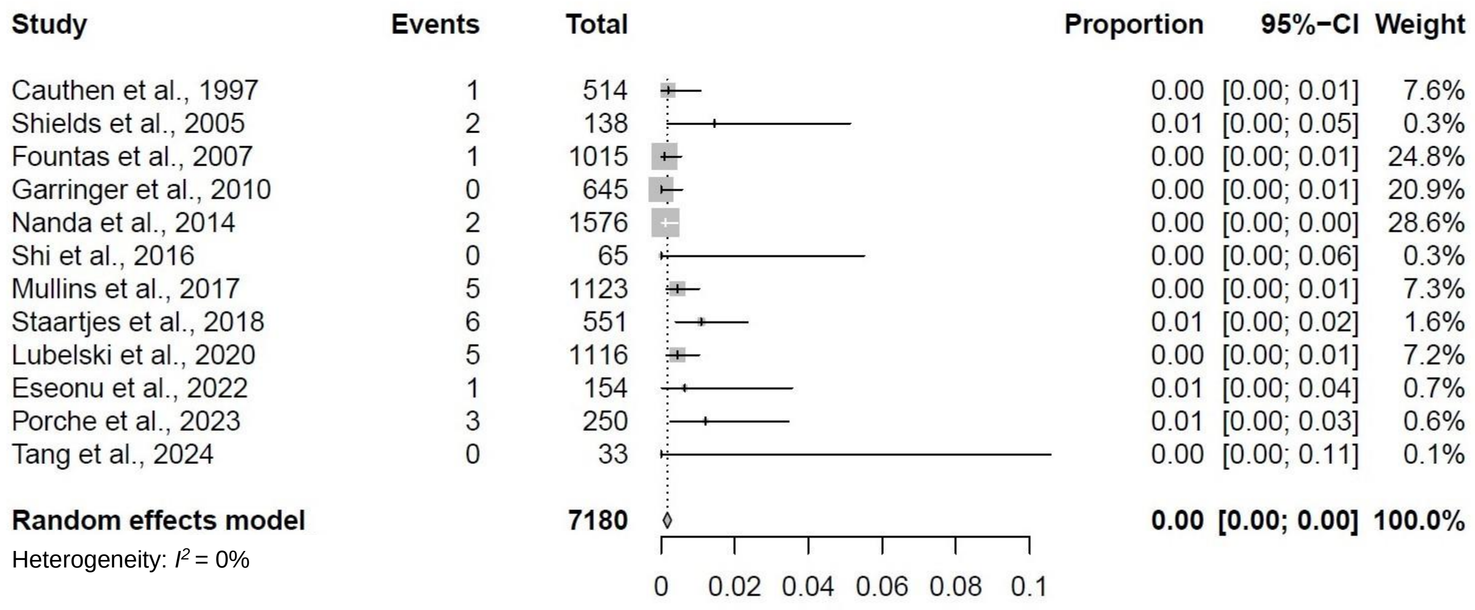

Supplementary Figure 11. Forest plot for recurrent laryngeal nerve palsy.

Study	Events	Total	Proportion	95%−CI	Weight
Bose et al., 2001	5	180	0.03	[0.01; 0.06]	4.3%
Shields et al., 2005	2	138	0.01	[0.00; 0.05]	4.6%
Hwang et al., 2005	0	78	0.00	[0.00; 0.05]	4.8%
Lied et al., 2006	1	390	0.00	[0.00; 0.01]	5.3%
Mobbs et al., 2007	2	242	0.01	[0.00; 0.03]	5.1%
Fountas et al., 2007	32	1015	0.03	[0.02; 0.04]	5.1%
Tumialán et al., 2008	2	200	0.01	[0.00; 0.04]	5.0%
Ghahreman et al., 2009	1	55	0.02	[0.00; 0.10]	3.6%
Kast et al., 2009	1	60	0.02	[0.00; 0.09]	3.8%
Uribe et al., 2009	0	42	0.00	[0.00; 0.08]	3.8%
Tumialán et al., 2010	2	19	0.11	[0.01; 0.33]	0.6%
Garringer et al., 2010	0	645	0.00	[0.00; 0.01]	5.3%
Joseffer et al., 2010	4	390	0.01	[0.00; 0.03]	5.1%
Lin et al., 2011	2	57	0.04	[0.00; 0.12]	2.8%
Lied et al., 2012	0	96	0.00	[0.00; 0.04]	4.9%
Nanda et al., 2014	2	1576	0.00	[0.00; 0.00]	5.3%
Shi et al., 2016	0	65	0.00	[0.00; 0.06]	4.5%
ElAbed et al., 2016	0	90	0.00	[0.00; 0.04]	4.9%
Scholz et al., 2018	1	40	0.02	[0.00; 0.13]	2.8%
Staartjes et al., 2018	66	551	0.12	[0.09; 0.15]	4.1%
Huschbeck et al., 2019	19	211	0.09	[0.06; 0.14]	3.4%
Gowd et al., 2021	0	108	0.00	[0.00; 0.03]	5.0%
Niljianskul et al., 2023	5	85	0.06	[0.02; 0.13]	2.7%
Tang et al., 2024	0	33	0.00	[0.00; 0.11]	3.2%
**Random effects model**		**6366**	**0.02**	**[0.01; 0.03]**	**100.0%**

Heterogeneity: $I^2 = 98\%$

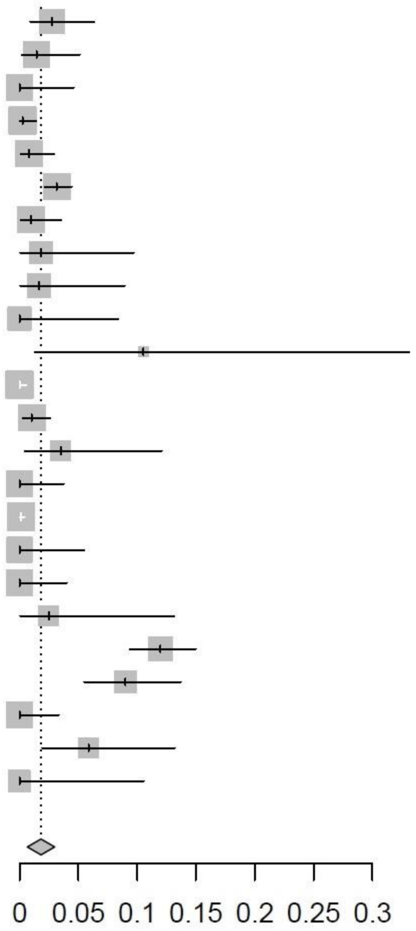

Supplementary Figure 12. Forest plot for hoarseness.

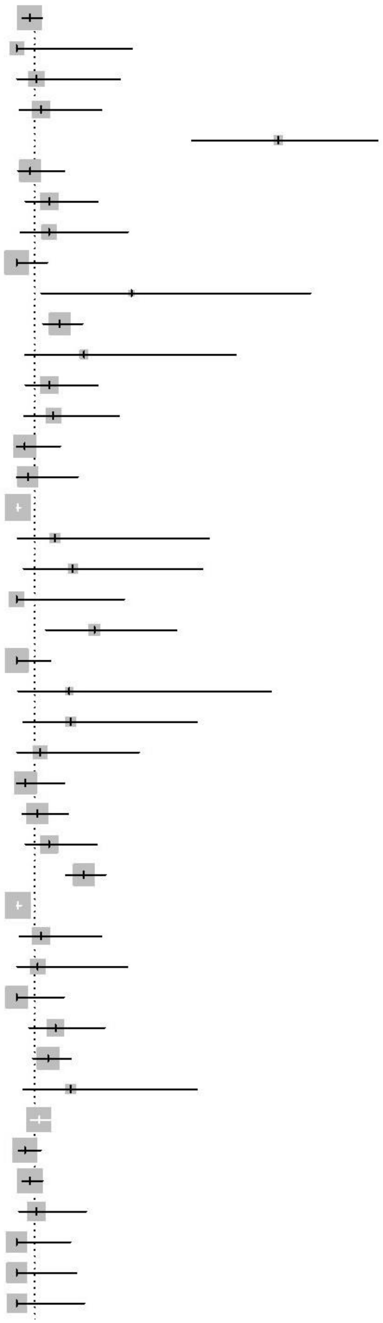

Study	Events	Total	Proportion	95%−CI	Weight
Cauthen et al., 1997	8	514	0.02	[0.01; 0.03]	3.9%
Wirth et al., 2000	0	25	0.00	[0.00; 0.14]	1.2%
Tureyen et al., 2003	1	43	0.02	[0.00; 0.12]	1.5%
Nirala et al., 2003	2	69	0.03	[0.00; 0.10]	1.8%
Yue et al., 2004	23	74	0.31	[0.21; 0.43]	0.4%
Korinth et al., 2006	2	124	0.02	[0.00; 0.06]	3.0%
Villavicencio et al., 2007	4	103	0.04	[0.01; 0.10]	1.9%
Aryan et al., 2007	2	52	0.04	[0.00; 0.13]	1.3%
Ruetten et al., 2008	0	100	0.00	[0.00; 0.04]	3.7%
Dogan et al., 2008	3	22	0.14	[0.03; 0.35]	0.2%
Joseffer et al., 2010	20	390	0.05	[0.03; 0.08]	3.0%
Song et al., 2012	2	25	0.08	[0.01; 0.26]	0.4%
Liu et al., 2012	4	103	0.04	[0.01; 0.10]	1.9%
Liu et al., 2012	3	69	0.04	[0.01; 0.12]	1.4%
Davis et al., 2013	1	105	0.01	[0.00; 0.05]	3.3%
Omidi−Kashani et al., 2014	1	74	0.01	[0.00; 0.07]	2.7%
Nanda et al., 2014	2	1576	0.00	[0.00; 0.00]	4.3%
Liu et al., 2015	1	22	0.05	[0.00; 0.23]	0.6%
Wang et al., 2015	2	30	0.07	[0.01; 0.22]	0.5%
Wang et al., 2015	0	27	0.00	[0.00; 0.13]	1.4%
Shi et al., 2016	6	65	0.09	[0.03; 0.19]	0.8%
ElAbed et al., 2016	0	90	0.00	[0.00; 0.04]	3.6%
Qizhi et al., 2016	1	16	0.06	[0.00; 0.30]	0.3%
Li et al., 2017	2	31	0.06	[0.01; 0.21]	0.6%
Riederman et al., 2017	1	36	0.03	[0.00; 0.15]	1.2%
Riederman et al., 2017	1	96	0.01	[0.00; 0.06]	3.2%
Riederman et al., 2017	4	164	0.02	[0.01; 0.06]	2.9%
Riederman et al., 2017	4	104	0.04	[0.01; 0.10]	2.0%
Staartjes et al., 2018	44	551	0.08	[0.06; 0.11]	3.0%
Helseth et al., 2019	2	1300	0.00	[0.00; 0.01]	4.3%
Yeung et al., 2019	2	69	0.03	[0.00; 0.10]	1.8%
Xu et al., 2019	1	40	0.02	[0.00; 0.13]	1.4%
Ahn et al., 2020	0	64	0.00	[0.00; 0.06]	3.1%
Gowd et al., 2021	5	108	0.05	[0.02; 0.10]	1.8%
Fayed et al., 2021	12	321	0.04	[0.02; 0.06]	3.1%
Niljianskul et al., 2021	2	31	0.06	[0.01; 0.21]	0.6%
Alsoof et al., 2022	21	792	0.03	[0.02; 0.04]	3.9%
Hao et al., 2022	3	302	0.01	[0.00; 0.03]	3.9%
Nayak et al., 2022	8	507	0.02	[0.01; 0.03]	3.9%
Niljianskul et al., 2023	2	85	0.02	[0.00; 0.08]	2.2%
Wang et al., 2023	0	56	0.00	[0.00; 0.06]	2.8%
Wang et al., 2023	0	50	0.00	[0.00; 0.07]	2.6%
Wang et al., 2023	0	44	0.00	[0.00; 0.08]	2.4%

Tang et al., 2023	0	31	0.00	[0.00; 0.11]	1.6%
Tang et al., 2023	0	36	0.00	[0.00; 0.10]	1.9%
Tang et al., 2024	0	33	0.00	[0.00; 0.11]	1.8%
Zaho et al., 2024	1	13	0.08	[0.00; 0.36]	0.2%
Zaho et al., 2024	2	30	0.07	[0.01; 0.22]	0.5%
**Random effects model**		**8612**	**0.02**	**[0.01; 0.03]**	**100.0%**

Heterogeneity: $I^2 = 88\%$

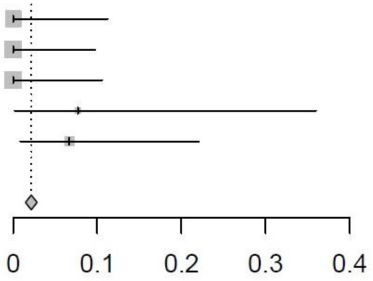

Supplementary Figure 13. Forest plot for cage/graft subsidence.

Study	Events	Total	Proportion	95%−CI	Weight
Swank et al., 1996	29	64	0.45	[0.33; 0.58]	1.4%
Kaiser et al., 2002	0	251	0.00	[0.00; 0.01]	1.6%
Nirala et al., 2003	1	69	0.01	[0.00; 0.08]	1.6%
Shields et al., 2005	1	138	0.01	[0.00; 0.04]	1.6%
Mobbs et al., 2007	1	242	0.00	[0.00; 0.02]	1.6%
Fountas et al., 2007	1	1015	0.00	[0.00; 0.01]	1.6%
Chang−Jung et al., 2008	8	56	0.14	[0.06; 0.26]	1.5%
Dogan et al., 2008	10	22	0.45	[0.24; 0.68]	1.1%
Chou et al., 2008	7	55	0.13	[0.05; 0.24]	1.5%
Kast et al., 2009	17	60	0.28	[0.17; 0.41]	1.4%
Uribe et al., 2009	0	42	0.00	[0.00; 0.08]	1.6%
Garringer et al., 2010	0	645	0.00	[0.00; 0.01]	1.6%
Guo et al., 2010	5	53	0.09	[0.03; 0.21]	1.5%
Liu et al., 2012	0	103	0.00	[0.00; 0.04]	1.6%
Wu et al., 2012	13	67	0.19	[0.11; 0.31]	1.5%
Lied et al., 2012	0	96	0.00	[0.00; 0.04]	1.6%
Yamagata et al., 2012	11	47	0.23	[0.12; 0.38]	1.4%
Park et al., 2013	7	31	0.23	[0.10; 0.41]	1.3%
Kao et al., 2014	69	182	0.38	[0.31; 0.45]	1.5%
Yoo et al., 2014	18	58	0.31	[0.20; 0.45]	1.4%
Omidi−Kashani et al., 2014	8	74	0.11	[0.05; 0.20]	1.5%
Lee et al., 2014	26	78	0.33	[0.23; 0.45]	1.5%
Shiban et al., 2015	66	265	0.25	[0.20; 0.31]	1.6%
Shi et al., 2016	1	68	0.01	[0.00; 0.08]	1.6%
ElAbed et al., 2016	7	90	0.08	[0.03; 0.15]	1.6%
Yamagata et al., 2016	8	45	0.18	[0.08; 0.32]	1.4%
Yamagata et al., 2016	11	55	0.20	[0.10; 0.33]	1.5%
Phan et al., 2017	1	270	0.00	[0.00; 0.02]	1.6%
Phan et al., 2017	0	171	0.00	[0.00; 0.02]	1.6%
Li et al., 2017	0	31	0.00	[0.00; 0.11]	1.6%
Phan et al., 2017	0	444	0.00	[0.00; 0.01]	1.6%
Mullins et al., 2017	3	1123	0.00	[0.00; 0.01]	1.6%
Phan et al., 2017	0	760	0.00	[0.00; 0.00]	1.6%
Phan et al., 2017	1	760	0.00	[0.00; 0.01]	1.6%
Phan et al., 2017	0	760	0.00	[0.00; 0.00]	1.6%
Phan et al., 2017	0	760	0.00	[0.00; 0.00]	1.6%
Phan et al., 2017	0	761	0.00	[0.00; 0.00]	1.6%
Grasso et al., 2018	0	100	0.00	[0.00; 0.04]	1.6%
Wang et al., 2018	0	26	0.00	[0.00; 0.13]	1.6%
Zhang et al., 2018	2	77	0.03	[0.00; 0.09]	1.6%
Oni et al., 2018	2	85	0.02	[0.00; 0.08]	1.6%
Kim et al., 2018	37	37	1.00	[0.91; 1.00]	1.6%
Kim et al., 2018	0	31	0.00	[0.00; 0.11]	1.6%
Bivo et al., 2019	9	96	0.09	[0.04; 0.17]	1.6%

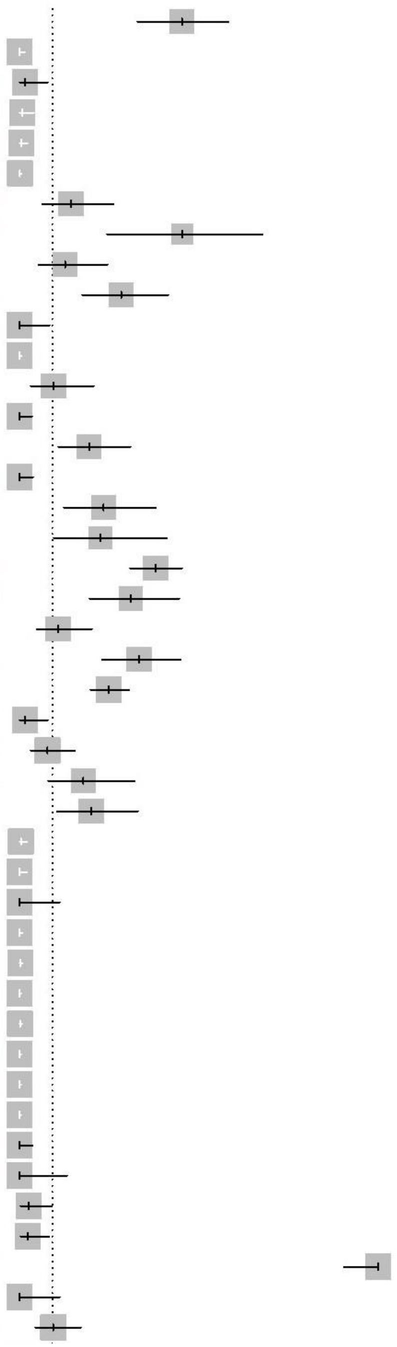

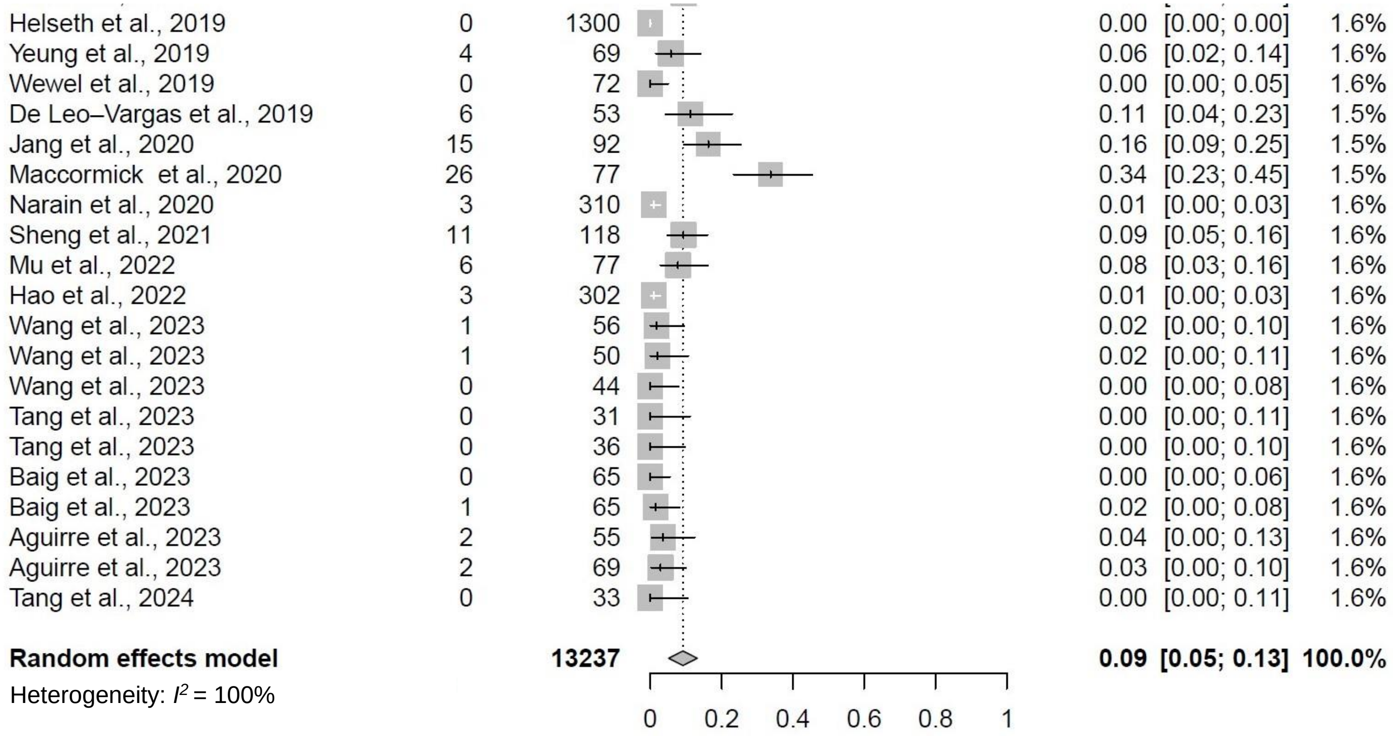
Helseth et al., 2019 0 1300 0.00 [0.00; 0.00] 1.6%
Yeung et al., 2019 4 69 0.06 [0.02; 0.14] 1.6%
Wewel et al., 2019 0 72 0.00 [0.00; 0.05] 1.6%
De Leo–Vargas et al., 2019 6 53 0.11 [0.04; 0.23] 1.5%
Jang et al., 2020 15 92 0.16 [0.09; 0.25] 1.5%
Maccormick et al., 2020 26 77 0.34 [0.23; 0.45] 1.5%
Narain et al., 2020 3 310 0.01 [0.00; 0.03] 1.6%
Sheng et al., 2021 11 118 0.09 [0.05; 0.16] 1.6%
Mu et al., 2022 6 77 0.08 [0.03; 0.16] 1.6%
Hao et al., 2022 3 302 0.01 [0.00; 0.03] 1.6%
Wang et al., 2023 1 56 0.02 [0.00; 0.10] 1.6%
Wang et al., 2023 1 50 0.02 [0.00; 0.11] 1.6%
Wang et al., 2023 0 44 0.00 [0.00; 0.08] 1.6%
Tang et al., 2023 0 31 0.00 [0.00; 0.11] 1.6%
Tang et al., 2023 0 36 0.00 [0.00; 0.10] 1.6%
Baig et al., 2023 0 65 0.00 [0.00; 0.06] 1.6%
Baig et al., 2023 1 65 0.02 [0.00; 0.08] 1.6%
Aguirre et al., 2023 2 55 0.04 [0.00; 0.13] 1.6%
Aguirre et al., 2023 2 69 0.03 [0.00; 0.10] 1.6%
Tang et al., 2024 0 33 0.00 [0.00; 0.11] 1.6%
Random effects model 13237 0.09 [0.05; 0.13] 100.0%
Heterogeneity: I2 = 100%
0 0.2 0.4 0.6 0.8 1

Supplementary Figure 14. Forest plot for C5 nerve root palsy.

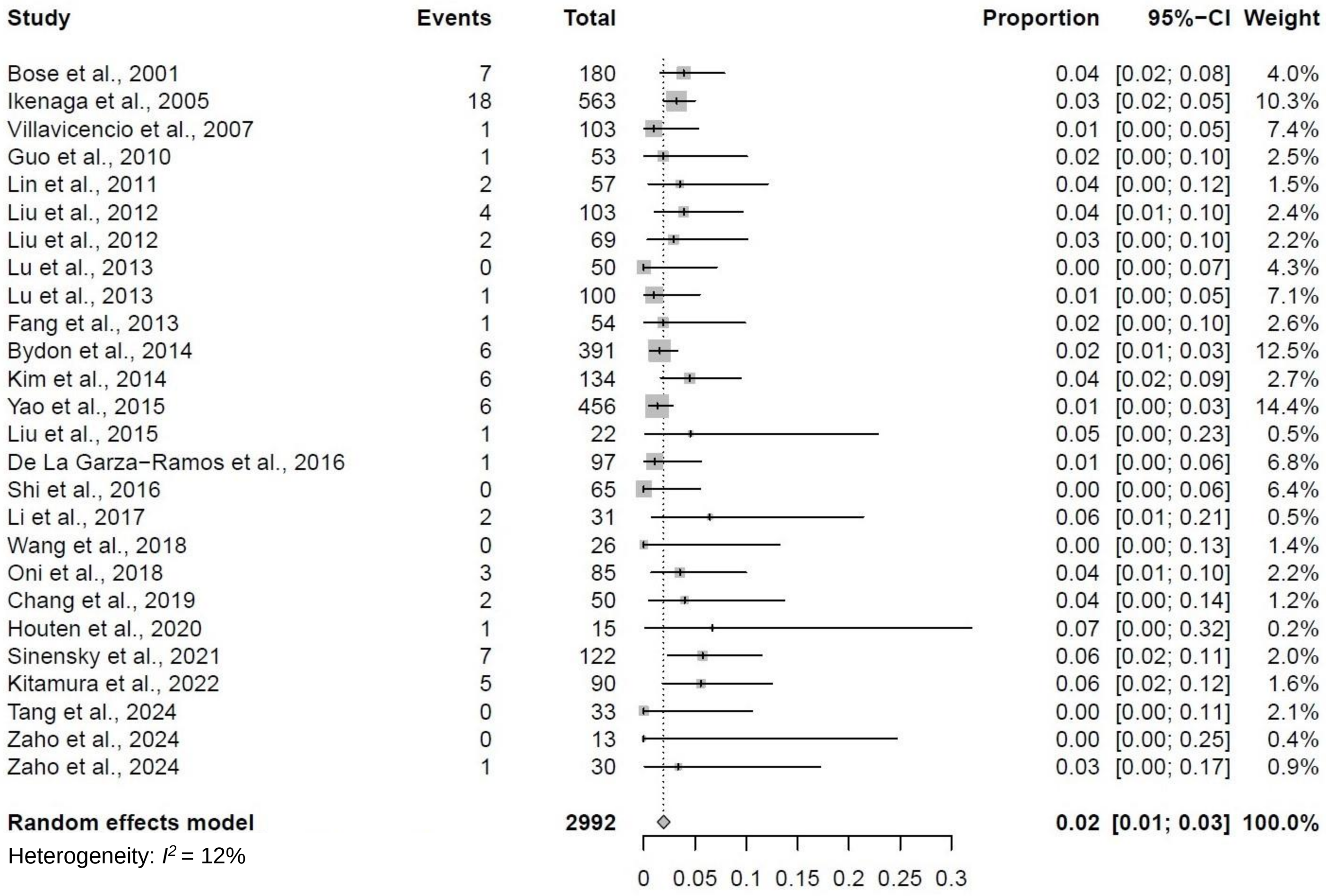

Supplementary Figure 15. Forest plot for cerebrospinal fluid leak.

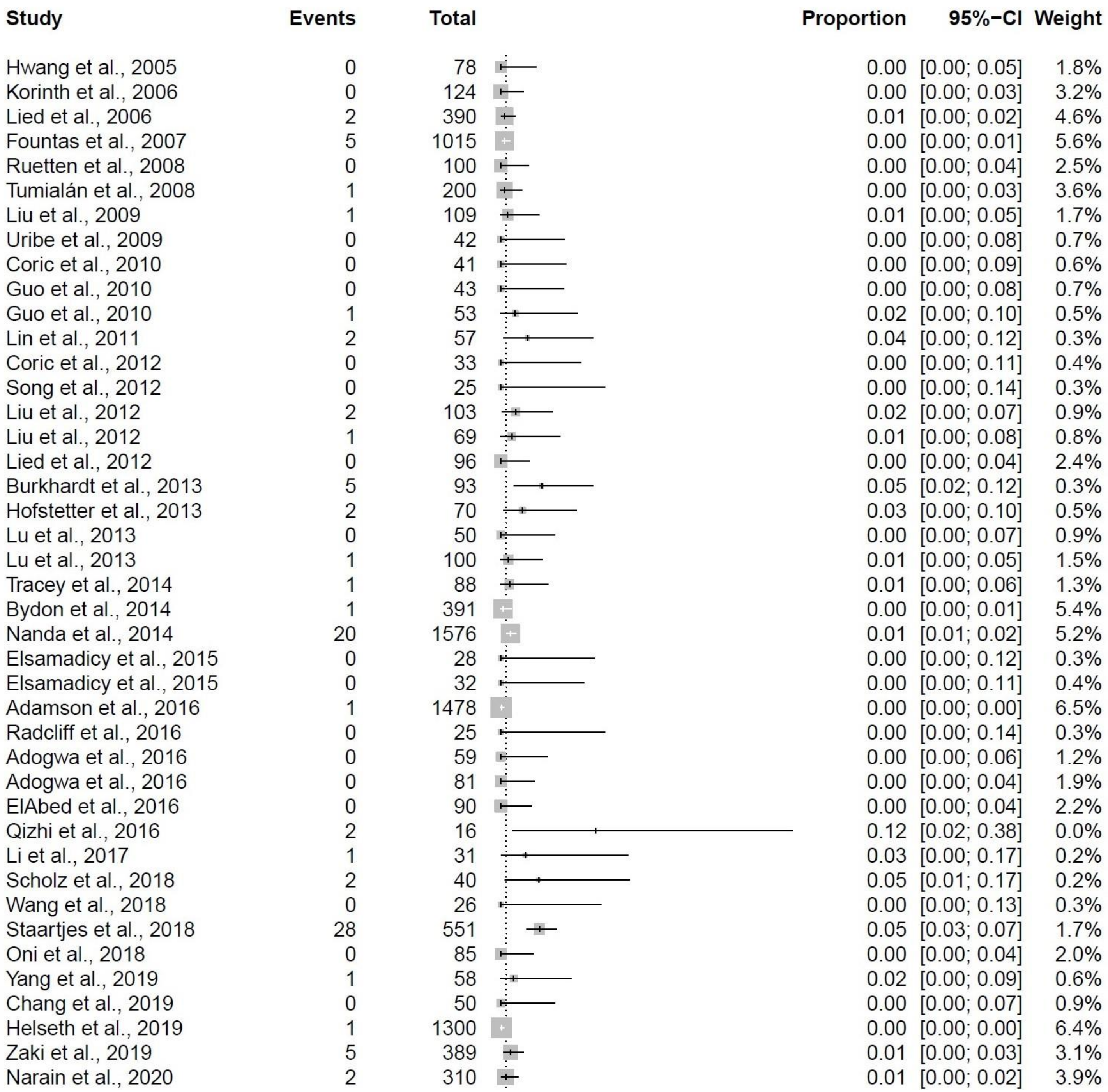

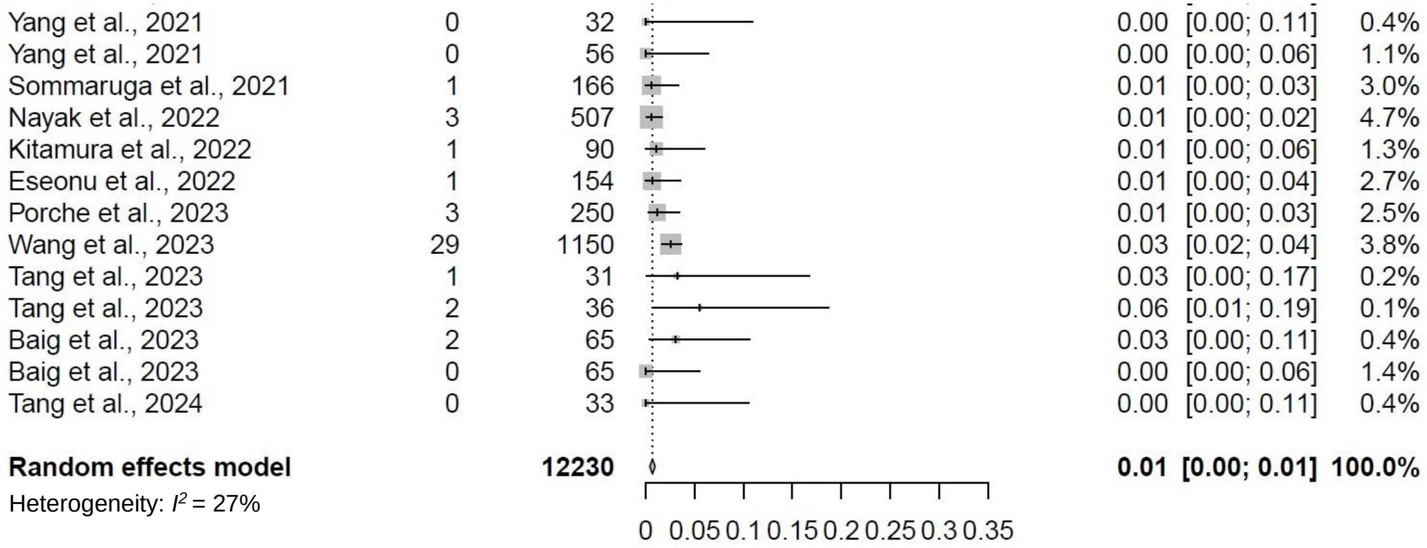
Yang et al., 2021 0 32 0.00 [0.00; 0.11] 0.4%
Yang et al., 2021 0 56 0.00 [0.00; 0.06] 1.1%
Sommaruga et al., 2021 1 166 0.01 [0.00; 0.03] 3.0%
Nayak et al., 2022 3 507 0.01 [0.00; 0.02] 4.7%
Kitamura et al., 2022 1 90 0.01 [0.00; 0.06] 1.3%
Eseonu et al., 2022 1 154 0.01 [0.00; 0.04] 2.7%
Porche et al., 2023 3 250 0.01 [0.00; 0.03] 2.5%
Wang et al., 2023 29 1150 0.03 [0.02; 0.04] 3.8%
Tang et al., 2023 1 31 0.03 [0.00; 0.17] 0.2%
Tang et al., 2023 2 36 0.06 [0.01; 0.19] 0.1%
Baig et al., 2023 2 65 0.03 [0.00; 0.11] 0.4%
Baig et al., 2023 0 65 0.00 [0.00; 0.06] 1.4%
Tang et al., 2024 0 33 0.00 [0.00; 0.11] 0.4%
Random effects model 12230 0.01 [0.00; 0.01] 100.0%
Heterogeneity: I2 = 27%
0 0.05 0.1 0.15 0.2 0.25 0.3 0.35

Supplementary Figure 16. Forest plot for pneumonia.

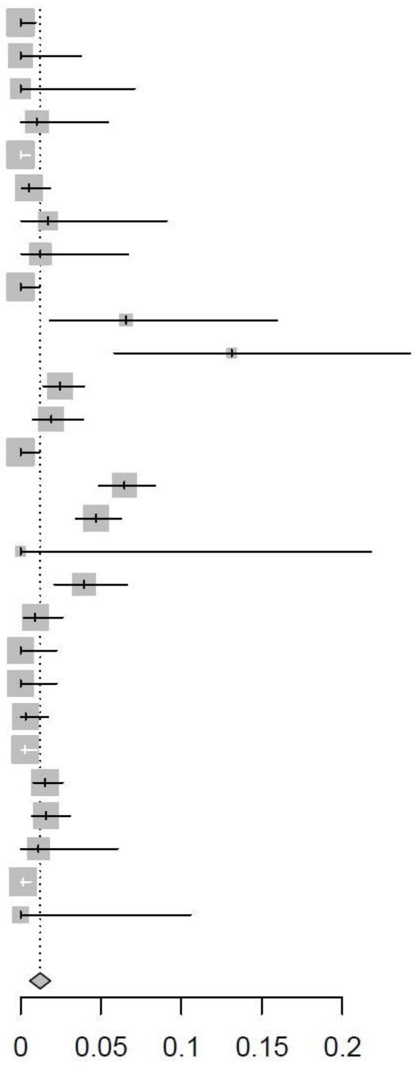

Study	Events	Total	Proportion	95%−CI	Weight
Lied et al., 2006	0	390	0.00	[0.00; 0.01]	4.8%
Lied et al., 2012	0	96	0.00	[0.00; 0.04]	3.9%
Lu et al., 2013	0	50	0.00	[0.00; 0.07]	2.6%
Lu et al., 2013	1	100	0.01	[0.00; 0.05]	3.3%
Martin et al., 2014	0	597	0.00	[0.00; 0.01]	4.8%
Minhas et al., 2014	2	387	0.01	[0.00; 0.02]	4.6%
Adogwa et al., 2016	1	59	0.02	[0.00; 0.09]	2.1%
Adogwa et al., 2016	1	81	0.01	[0.00; 0.07]	2.9%
Yagi et al., 2017	0	307	0.00	[0.00; 0.01]	4.7%
Chughtai et al., 2018	4	61	0.07	[0.02; 0.16]	0.9%
Chughtai et al., 2018	8	61	0.13	[0.06; 0.24]	0.5%
Malik et al., 2019	16	649	0.02	[0.01; 0.04]	4.1%
Shuman et al., 2020	7	365	0.02	[0.01; 0.04]	3.9%
Narain et al., 2020	0	310	0.00	[0.00; 0.01]	4.7%
Neifert et al., 2020	53	821	0.06	[0.05; 0.08]	3.6%
Neifert et al., 2020	44	938	0.05	[0.03; 0.06]	4.0%
Houten et al., 2020	0	15	0.00	[0.00; 0.22]	0.5%
Wang et al., 2021	13	331	0.04	[0.02; 0.07]	3.2%
Wang et al., 2021	3	331	0.01	[0.00; 0.03]	4.3%
Patel et al., 2022	0	161	0.00	[0.00; 0.02]	4.4%
Patel et al., 2022	0	161	0.00	[0.00; 0.02]	4.4%
Patel et al., 2022	1	320	0.00	[0.00; 0.02]	4.6%
Ng et al., 2022	2	684	0.00	[0.00; 0.01]	4.7%
Alsoof et al., 2022	12	792	0.02	[0.01; 0.03]	4.5%
Nayak et al., 2022	8	507	0.02	[0.01; 0.03]	4.2%
Kitamura et al., 2022	1	90	0.01	[0.00; 0.06]	3.1%
Wang et al., 2023	2	1150	0.00	[0.00; 0.01]	4.8%
Tang et al., 2024	0	33	0.00	[0.00; 0.11]	1.7%
**Random effects model**		**9847**	**0.01**	**[0.01; 0.02]**	**100.0%**

Heterogeneity: I^2 = 95%

Supplementary Figure 17. Forest plot for pulmonary complications.

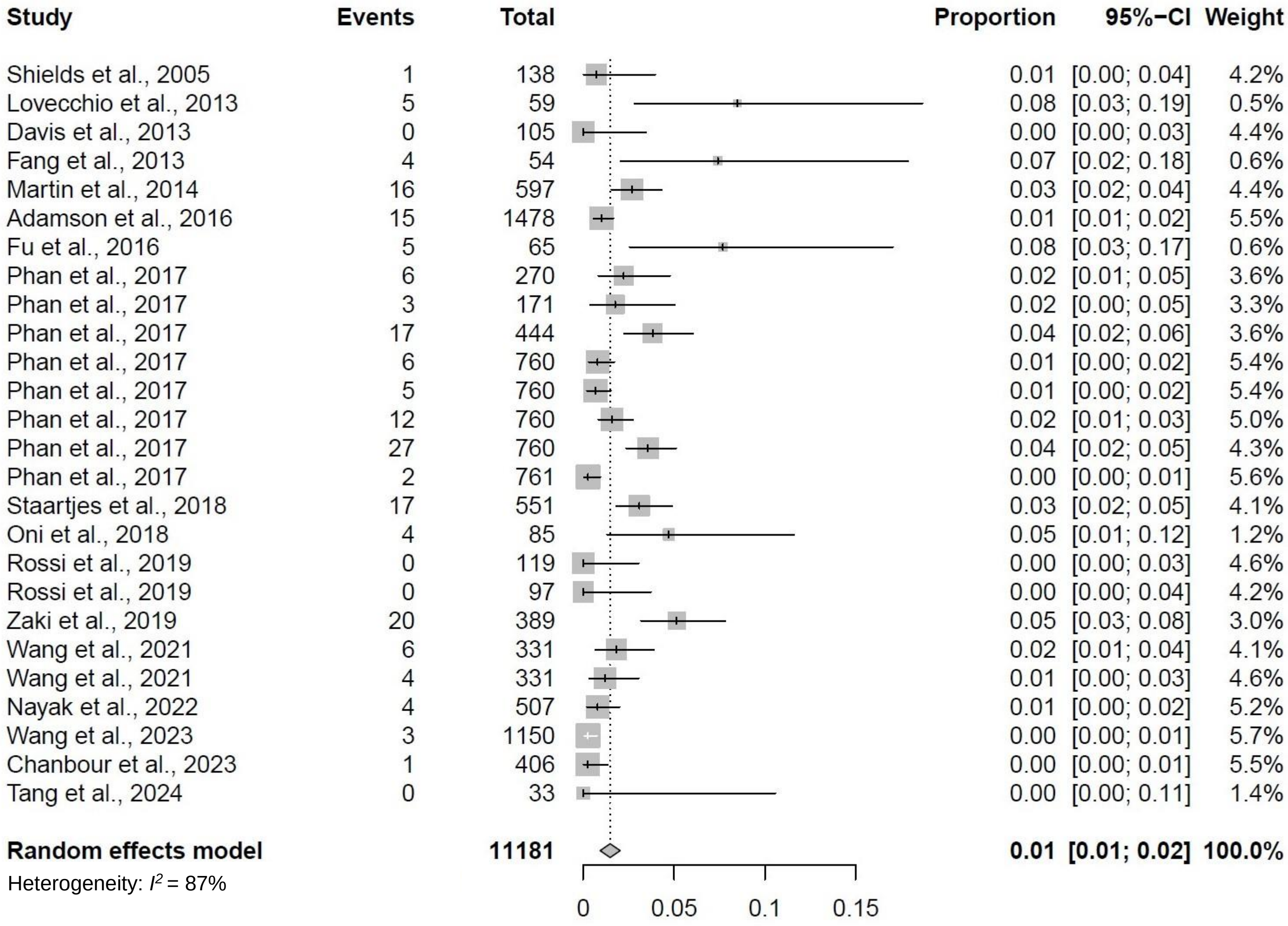

Supplementary Figure 18. Forest plot for pharyngeal/esophageal perforation.

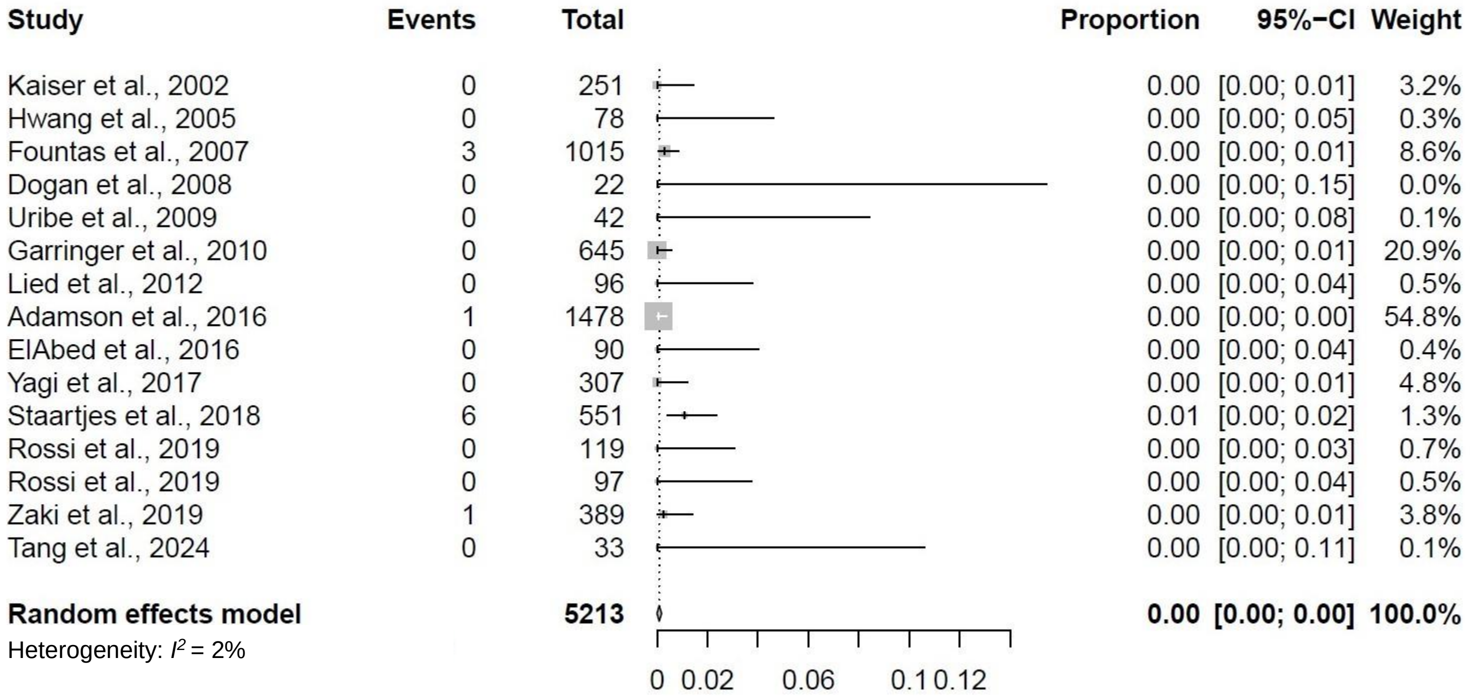

Supplementary Figure 19. Forest plot for feeding tube placement requirement.

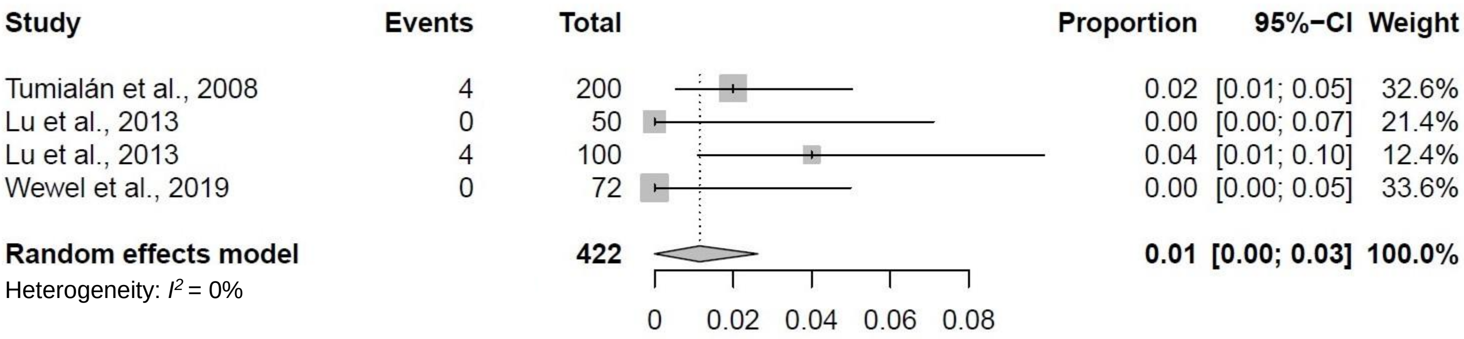

Supplementary Figure 20. Forest plot for deep vein thrombosis.

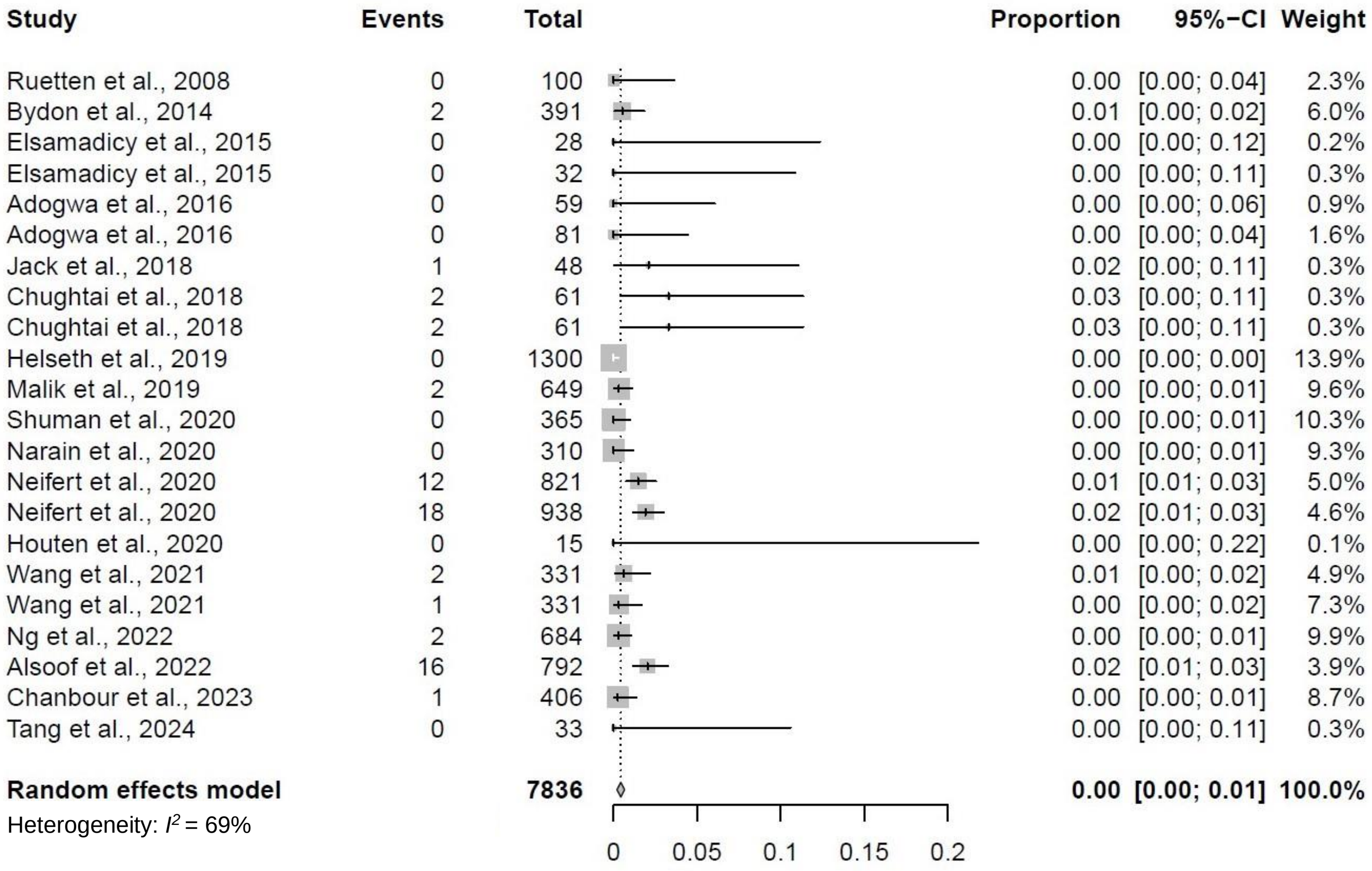

Supplementary Figure 21. Forest plot for pulmonary embolism.

Study	Events	Total	Proportion	95%−CI	Weight
Lied et al., 2006	1	390	0.00	[0.00; 0.01]	5.4%
Ruetten et al., 2008	0	100	0.00	[0.00; 0.04]	0.9%
Bydon et al., 2014	1	391	0.00	[0.00; 0.01]	5.4%
Elsamadicy et al., 2015	0	28	0.00	[0.00; 0.12]	0.1%
Elsamadicy et al., 2015	0	32	0.00	[0.00; 0.11]	0.1%
Fu et al., 2016	1	65	0.02	[0.00; 0.08]	0.2%
Adogwa et al., 2016	0	59	0.00	[0.00; 0.06]	0.3%
Adogwa et al., 2016	0	81	0.00	[0.00; 0.04]	0.6%
Chughtai et al., 2018	2	61	0.03	[0.00; 0.11]	0.1%
Chughtai et al., 2018	1	61	0.02	[0.00; 0.09]	0.2%
Helseth et al., 2019	0	1300	0.00	[0.00; 0.00]	21.7%
Malik et al., 2019	0	649	0.00	[0.00; 0.01]	15.2%
Shuman et al., 2020	0	365	0.00	[0.00; 0.01]	8.2%
Narain et al., 2020	0	310	0.00	[0.00; 0.01]	6.5%
Neifert et al., 2020	6	821	0.01	[0.00; 0.02]	4.2%
Neifert et al., 2020	5	938	0.01	[0.00; 0.01]	6.1%
Wang et al., 2021	1	331	0.00	[0.00; 0.02]	4.1%
Wang et al., 2021	2	331	0.01	[0.00; 0.02]	2.3%
Patel et al., 2022	0	161	0.00	[0.00; 0.02]	2.2%
Patel et al., 2022	0	161	0.00	[0.00; 0.02]	2.2%
Patel et al., 2022	1	320	0.00	[0.00; 0.02]	3.9%
Ng et al., 2022	2	684	0.00	[0.00; 0.01]	7.4%
Nayak et al., 2022	5	507	0.01	[0.00; 0.02]	2.1%
Aguirre et al., 2023	1	55	0.02	[0.00; 0.10]	0.1%
Aguirre et al., 2023	0	69	0.00	[0.00; 0.05]	0.4%
Tang et al., 2024	0	33	0.00	[0.00; 0.11]	0.1%
**Random effects model**		**8303**	**0.00**	**[0.00; 0.00]**	**100.0%**

Heterogeneity: $I^2 = 29\%$

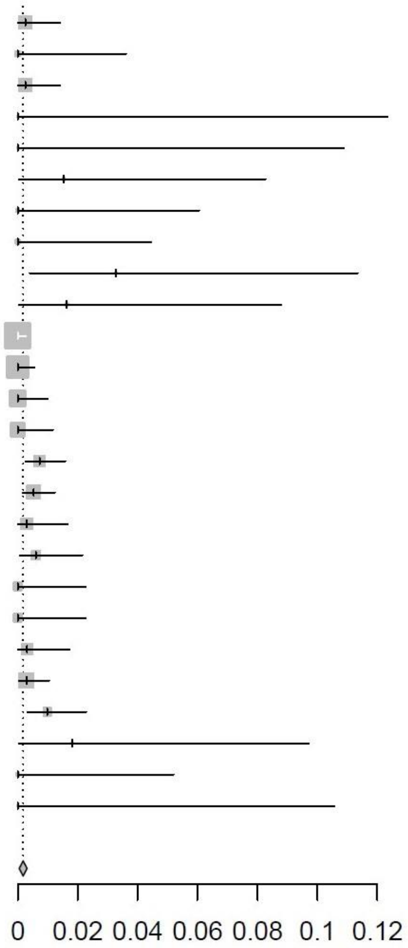

Supplementary Figure 22. Forest plot for venous thromboembolism.

Study	Events	Total	Proportion	95%−CI	Weight
Ruetten et al., 2008	0	100	0.00	[0.00; 0.04]	1.0%
Garringer et al., 2010	0	645	0.00	[0.00; 0.01]	17.5%
Phan et al., 2017	2	270	0.01	[0.00; 0.03]	1.8%
Phan et al., 2017	0	171	0.00	[0.00; 0.02]	2.8%
Phan et al., 2017	1	444	0.00	[0.00; 0.01]	7.6%
Phan et al., 2017	1	760	0.00	[0.00; 0.01]	14.8%
Phan et al., 2017	2	760	0.00	[0.00; 0.01]	9.9%
Phan et al., 2017	4	760	0.01	[0.00; 0.01]	6.0%
Phan et al., 2017	8	760	0.01	[0.00; 0.02]	3.3%
Phan et al., 2017	3	761	0.00	[0.00; 0.01]	7.5%
Helseth et al., 2019	0	1300	0.00	[0.00; 0.00]	25.2%
Zaki et al., 2019	3	389	0.01	[0.00; 0.02]	2.4%
Tang et al., 2024	0	33	0.00	[0.00; 0.11]	0.1%
**Random effects model**		**7153**	**0.00**	**[0.00; 0.00]**	**100.0%**

Heterogeneity: $I^2 = 32\%$

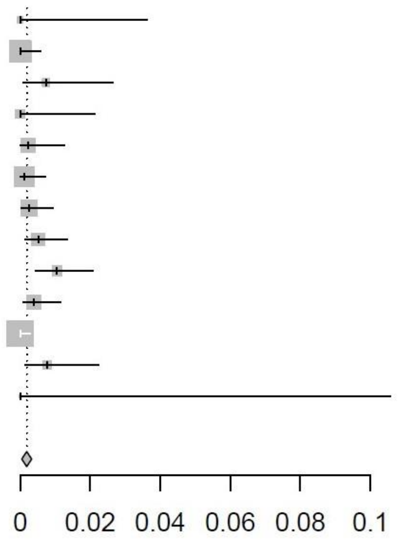

Supplementary Figure 23. Forest plot for worsening myelopathy.

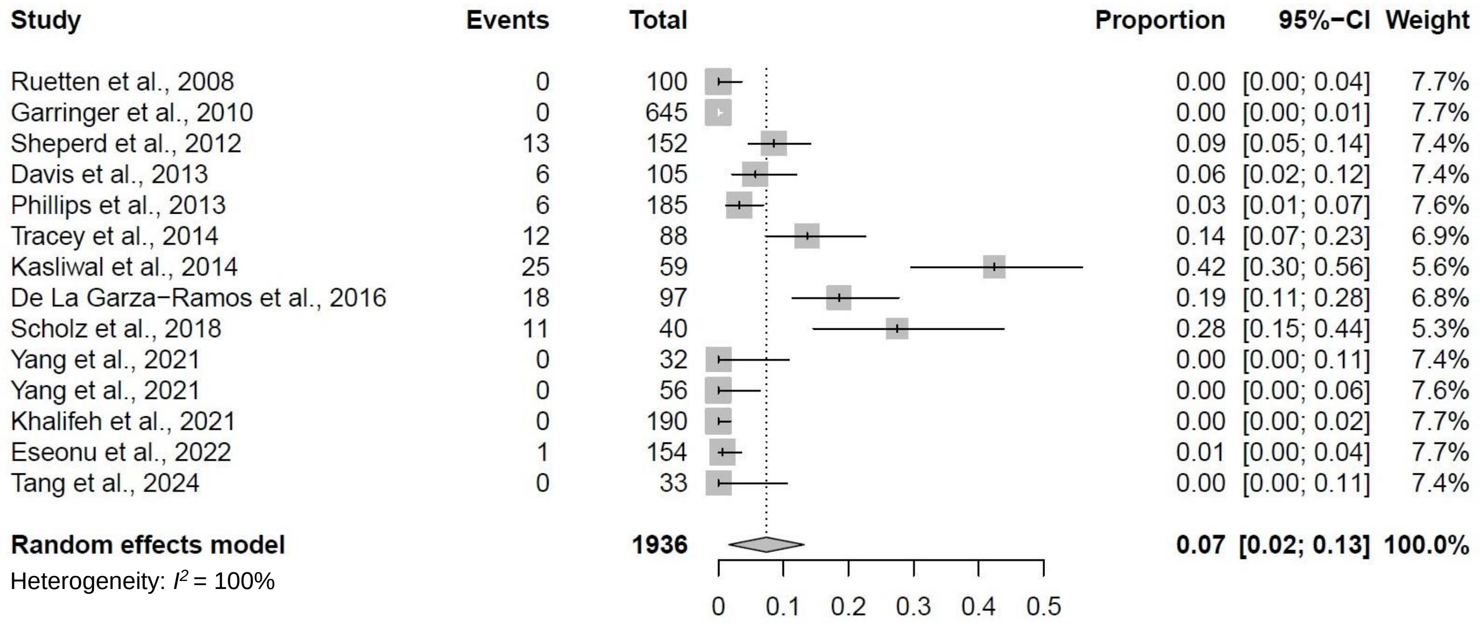

Supplementary Figure 24. Forest plot for worsening radiculopathy.

Study	Events	Total	Proportion	95%−CI	Weight
Korinth et al., 2006	1	124	0.01	[0.00; 0.04]	1.0%
Mobbs et al., 2007	1	242	0.00	[0.00; 0.02]	3.3%
Ruetten et al., 2008	0	100	0.00	[0.00; 0.04]	1.3%
Chou et al., 2008	2	55	0.04	[0.00; 0.13]	0.1%
Rohe et al., 2009	1	54	0.02	[0.00; 0.10]	0.2%
Garringer et al., 2010	0	645	0.00	[0.00; 0.01]	17.6%
Sheperd et al., 2012	0	152	0.00	[0.00; 0.02]	2.7%
Coric et al., 2012	0	33	0.00	[0.00; 0.11]	0.2%
Lied et al., 2012	1	96	0.01	[0.00; 0.06]	0.6%
Davis et al., 2013	4	105	0.04	[0.01; 0.09]	0.2%
Tracey et al., 2014	12	88	0.14	[0.07; 0.23]	0.0%
Elsamadicy et al., 2015	0	28	0.00	[0.00; 0.12]	0.1%
Elsamadicy et al., 2015	0	32	0.00	[0.00; 0.11]	0.1%
De La Garza−Ramos et al., 2016	5	97	0.05	[0.02; 0.12]	0.1%
Shi et al., 2016	10	65	0.15	[0.08; 0.26]	0.0%
Adamson et al., 2016	4	1478	0.00	[0.00; 0.01]	15.1%
Adogwa et al., 2016	0	59	0.00	[0.00; 0.06]	0.5%
Adogwa et al., 2016	0	81	0.00	[0.00; 0.04]	0.8%
Phan et al., 2017	0	270	0.00	[0.00; 0.01]	7.0%
Phan et al., 2017	0	171	0.00	[0.00; 0.02]	3.4%
Lee et al., 2018	2	105	0.02	[0.00; 0.07]	0.4%
Scholz et al., 2018	13	40	0.32	[0.19; 0.49]	0.0%
Rossi et al., 2019	0	119	0.00	[0.00; 0.03]	1.7%
Rossi et al., 2019	0	97	0.00	[0.00; 0.04]	1.2%
Ahn et al., 2020	0	64	0.00	[0.00; 0.06]	0.5%
Yang et al., 2021	0	32	0.00	[0.00; 0.11]	0.1%
Yang et al., 2021	0	56	0.00	[0.00; 0.06]	0.4%
Khalifeh et al., 2021	0	190	0.00	[0.00; 0.02]	4.0%
Nayak et al., 2022	0	507	0.00	[0.00; 0.01]	14.7%
Wang et al., 2023	3	1150	0.00	[0.00; 0.01]	13.7%
Chanbour et al., 2023	1	406	0.00	[0.00; 0.01]	7.6%
Aguirre et al., 2023	0	55	0.00	[0.00; 0.06]	0.4%
Aguirre et al., 2023	0	69	0.00	[0.00; 0.05]	0.6%
Tang et al., 2024	0	33	0.00	[0.00; 0.11]	0.2%
Zaho et al., 2024	0	13	0.00	[0.00; 0.25]	0.0%
Zaho et al., 2024	0	30	0.00	[0.00; 0.12]	0.1%
**Random effects model**		**6941**	**0.00**	**[0.00; 0.00]**	**100.0%**

Heterogeneity: $I^2 = 35\%$

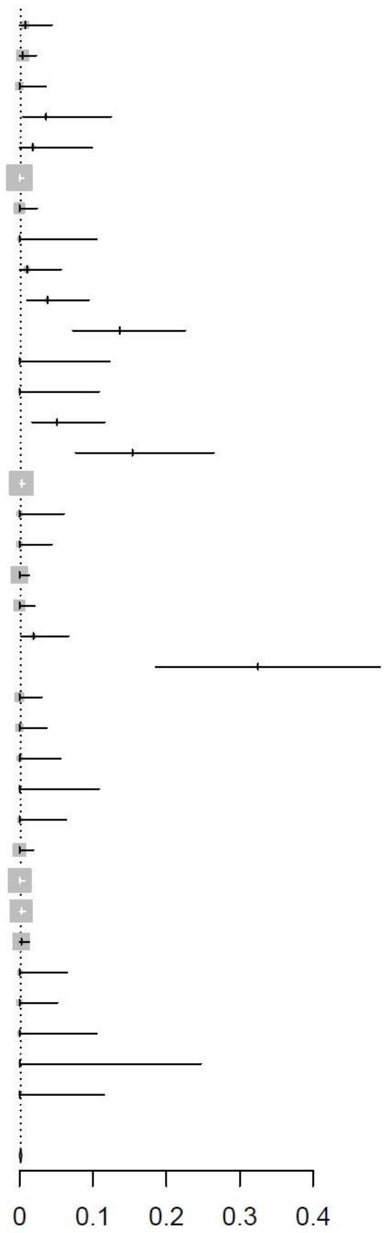

Supplementary Figure 25. Forest plot for excessive neck swelling.

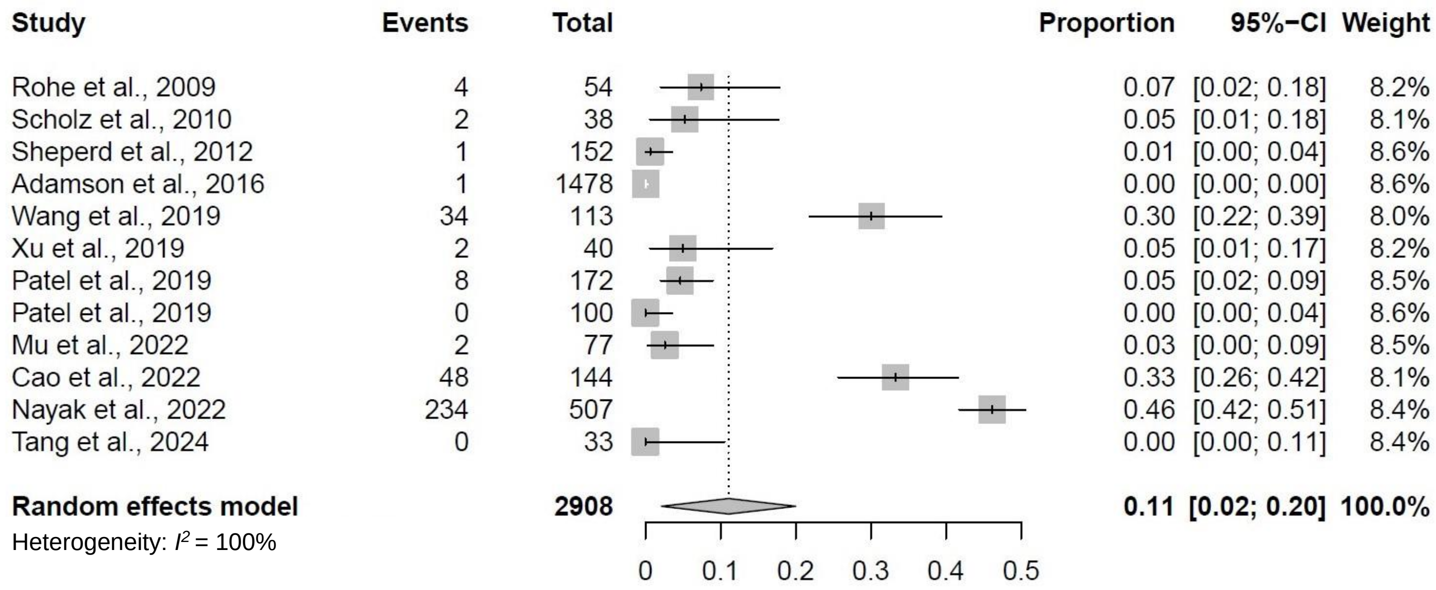

Supplementary Figure 26. Forest plot for urinary tract infection.

Study	Events	Total	Proportion	95%−CI	Weight
Cauthen et al., 1997	5	514	0.01	[0.00; 0.02]	3.8%
Lied et al., 2006	7	390	0.02	[0.01; 0.04]	2.5%
Lied et al., 2012	0	96	0.00	[0.00; 0.04]	2.3%
Lovecchio et al., 2013	5	59	0.08	[0.03; 0.19]	0.2%
Burkhardt et al., 2013	1	93	0.01	[0.00; 0.06]	1.4%
Nanda et al., 2014	2	1576	0.00	[0.00; 0.00]	5.6%
Elsamadicy et al., 2015	1	28	0.04	[0.00; 0.18]	0.2%
Elsamadicy et al., 2015	1	32	0.03	[0.00; 0.16]	0.2%
Adogwa et al., 2016	0	59	0.00	[0.00; 0.06]	1.2%
Adogwa et al., 2016	3	81	0.04	[0.01; 0.10]	0.4%
Phan et al., 2017	5	270	0.02	[0.01; 0.04]	2.0%
Phan et al., 2017	0	171	0.00	[0.00; 0.02]	3.9%
Phan et al., 2017	5	444	0.01	[0.00; 0.03]	3.4%
Phan et al., 2017	7	760	0.01	[0.00; 0.02]	4.3%
Phan et al., 2017	4	760	0.01	[0.00; 0.01]	4.8%
Phan et al., 2017	2	760	0.00	[0.00; 0.01]	5.2%
Phan et al., 2017	7	760	0.01	[0.00; 0.02]	4.3%
Phan et al., 2017	2	761	0.00	[0.00; 0.01]	5.2%
Yagi et al., 2017	0	307	0.00	[0.00; 0.01]	5.0%
Malik et al., 2019	5	649	0.01	[0.00; 0.02]	4.3%
Shuman et al., 2020	1	365	0.00	[0.00; 0.02]	4.7%
Narain et al., 2020	0	310	0.00	[0.00; 0.01]	5.0%
Neifert et al., 2020	11	821	0.01	[0.01; 0.02]	4.0%
Neifert et al., 2020	25	938	0.03	[0.02; 0.04]	3.2%
Patel et al., 2022	0	161	0.00	[0.00; 0.02]	3.8%
Patel et al., 2022	0	161	0.00	[0.00; 0.02]	3.8%
Patel et al., 2022	3	320	0.01	[0.00; 0.03]	3.2%
Ng et al., 2022	2	684	0.00	[0.00; 0.01]	5.1%
Nayak et al., 2022	22	507	0.04	[0.03; 0.06]	1.8%
Chanbour et al., 2023	1	406	0.00	[0.00; 0.01]	4.9%
Tang et al., 2024	0	33	0.00	[0.00; 0.11]	0.4%
**Random effects model**		**13276**	**0.01**	**[0.00; 0.01]**	**100.0%**

Heterogeneity: I^2 = 75%

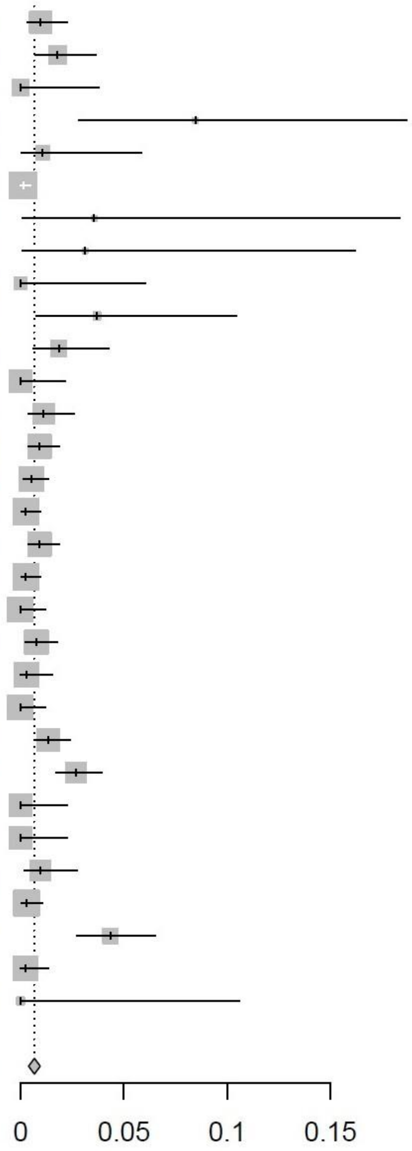

Supplementary Figure 27. Forest plot for cardiac complications.

Study	Events	Total	Proportion	95%−CI	Weight
Garringer et al., 2010	0	645	0.00	[0.00; 0.01]	6.1%
Lovecchio et al., 2013	0	59	0.00	[0.00; 0.06]	0.6%
Burkhardt et al., 2013	1	93	0.01	[0.00; 0.06]	0.8%
Fu et al., 2016	1	65	0.02	[0.00; 0.08]	0.4%
Phan et al., 2017	1	270	0.00	[0.00; 0.02]	3.4%
Phan et al., 2017	0	171	0.00	[0.00; 0.02]	3.1%
Phan et al., 2017	2	444	0.00	[0.00; 0.02]	3.9%
Phan et al., 2017	3	760	0.00	[0.00; 0.01]	4.9%
Phan et al., 2017	1	760	0.00	[0.00; 0.01]	5.9%
Phan et al., 2017	0	760	0.00	[0.00; 0.00]	6.2%
Phan et al., 2017	4	760	0.01	[0.00; 0.01]	4.5%
Phan et al., 2017	0	761	0.00	[0.00; 0.00]	6.2%
Chughtai et al., 2018	1	61	0.02	[0.00; 0.09]	0.4%
Chughtai et al., 2018	2	61	0.03	[0.00; 0.11]	0.2%
Malik et al., 2019	3	649	0.00	[0.00; 0.01]	4.5%
Patel et al., 2019	1	172	0.01	[0.00; 0.03]	2.0%
Patel et al., 2019	0	100	0.00	[0.00; 0.04]	1.5%
Zaki et al., 2019	14	389	0.04	[0.02; 0.06]	0.9%
Shuman et al., 2020	5	365	0.01	[0.00; 0.03]	1.9%
Narain et al., 2020	0	310	0.00	[0.00; 0.01]	4.9%
Neifert et al., 2020	4	821	0.00	[0.00; 0.01]	4.7%
Neifert et al., 2020	7	938	0.01	[0.00; 0.02]	4.3%
Houten et al., 2020	0	15	0.00	[0.00; 0.22]	0.1%
Wang et al., 2021	3	331	0.01	[0.00; 0.03]	2.3%
Wang et al., 2021	2	331	0.01	[0.00; 0.02]	3.0%
Patel et al., 2022	0	161	0.00	[0.00; 0.02]	2.9%
Patel et al., 2022	0	161	0.00	[0.00; 0.02]	2.9%
Patel et al., 2022	0	320	0.00	[0.00; 0.01]	5.0%
Ng et al., 2022	1	684	0.00	[0.00; 0.01]	5.8%
Nayak et al., 2022	44	507	0.09	[0.06; 0.11]	0.6%
Wang et al., 2023	2	1150	0.00	[0.00; 0.01]	6.0%
Tang et al., 2024	0	33	0.00	[0.00; 0.11]	0.2%
**Random effects model**		**13107**	**0.00**	**[0.00; 0.00]**	**100.0%**

Heterogeneity: $I^2 = 0\%$

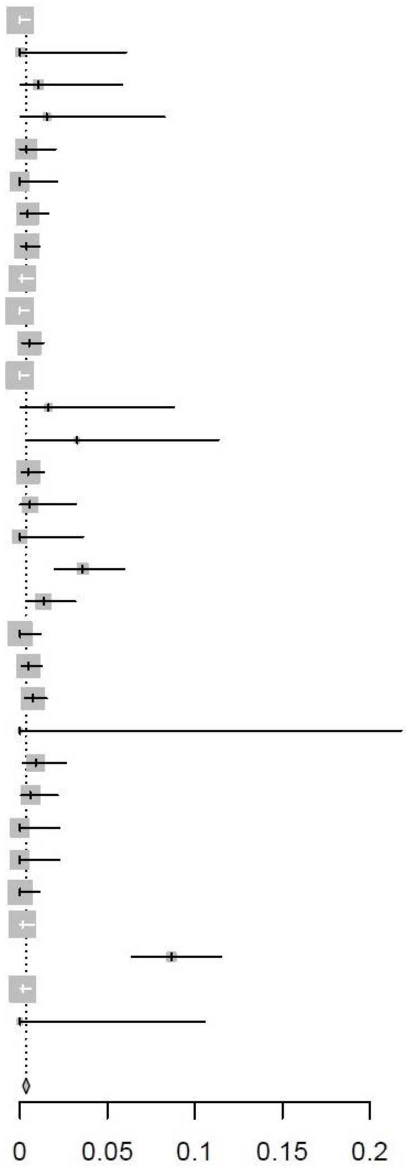

Supplementary Figure 28. Forest plot for non-home discharge.

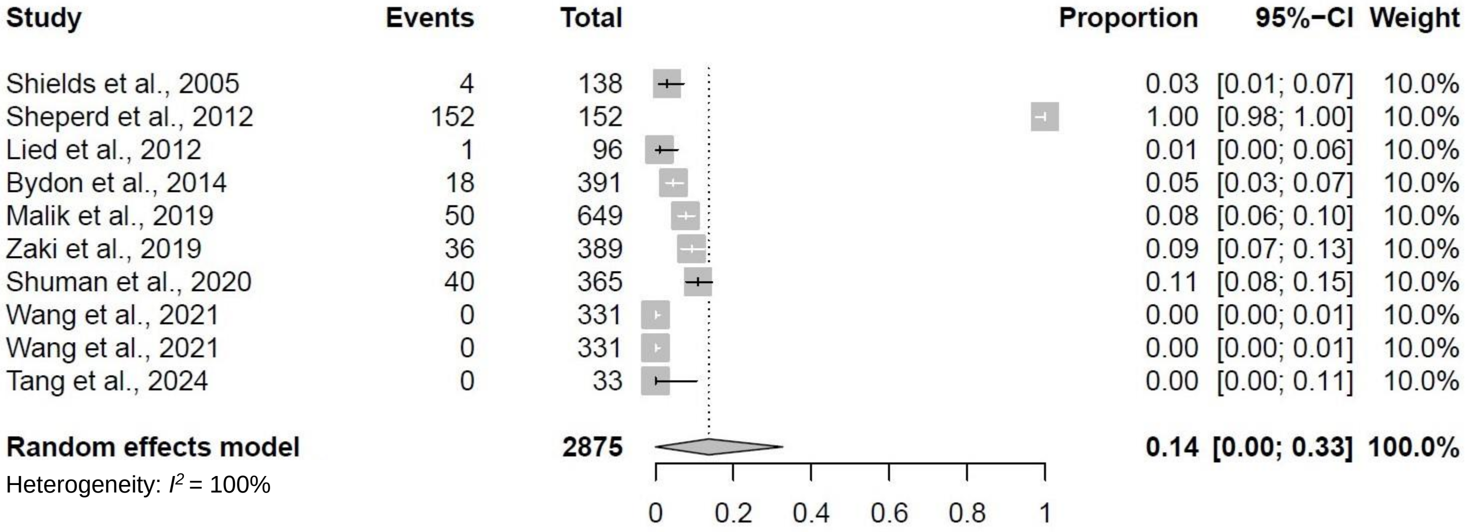

Supplementary Figure 29. Forest plot for readmission.

Study	Events	Total	Proportion	95%−CI	Weight
Shields et al., 2005	7	138	0.05	[0.02; 0.10]	2.2%
Tumialán et al., 2008	3	200	0.01	[0.00; 0.04]	2.5%
Trahan et al., 2011	1	59	0.02	[0.00; 0.09]	2.2%
Sheperd et al., 2012	1	152	0.01	[0.00; 0.04]	2.6%
Lu et al., 2013	0	50	0.00	[0.00; 0.07]	2.4%
Lu et al., 2013	23	100	0.23	[0.15; 0.32]	1.2%
Tally et al., 2013	0	119	0.00	[0.00; 0.03]	2.6%
Mansfield et al., 2014	0	76	0.00	[0.00; 0.05]	2.5%
Elsamadicy et al., 2015	5	28	0.18	[0.06; 0.37]	0.6%
Elsamadicy et al., 2015	1	32	0.03	[0.00; 0.16]	1.6%
Adamson et al., 2016	16	1478	0.01	[0.01; 0.02]	2.7%
Adogwa et al., 2016	6	59	0.10	[0.04; 0.21]	1.3%
Adogwa et al., 2016	4	81	0.05	[0.01; 0.12]	1.9%
Lovasik et al., 2016	5	191	0.03	[0.01; 0.06]	2.5%
Phan et al., 2017	7	270	0.03	[0.01; 0.05]	2.5%
Phan et al., 2017	13	171	0.08	[0.04; 0.13]	2.1%
Phan et al., 2017	27	444	0.06	[0.04; 0.09]	2.5%
Phan et al., 2017	11	760	0.01	[0.01; 0.03]	2.6%
Phan et al., 2017	8	760	0.01	[0.00; 0.02]	2.6%
Phan et al., 2017	12	760	0.02	[0.01; 0.03]	2.6%
Phan et al., 2017	14	760	0.02	[0.01; 0.03]	2.6%
Phan et al., 2017	5	761	0.01	[0.00; 0.02]	2.7%
Rossi et al., 2019	2	119	0.02	[0.00; 0.06]	2.4%
Rossi et al., 2019	2	97	0.02	[0.00; 0.07]	2.3%
Zaki et al., 2019	30	389	0.08	[0.05; 0.11]	2.4%
Shuman et al., 2020	31	365	0.08	[0.06; 0.12]	2.3%
Labaran et al., 2020	193	992	0.19	[0.17; 0.22]	2.4%
Yang et al., 2021	0	32	0.00	[0.00; 0.11]	2.0%
Yang et al., 2021	1	56	0.02	[0.00; 0.10]	2.2%
Fayed et al., 2021	12	321	0.04	[0.02; 0.06]	2.5%
Khalifeh et al., 2021	0	190	0.00	[0.00; 0.02]	2.6%
Wang et al., 2021	19	331	0.06	[0.03; 0.09]	2.4%
Wang et al., 2021	8	331	0.02	[0.01; 0.05]	2.5%
Kamalapathy et al., 2021	49	509	0.10	[0.07; 0.13]	2.4%
Leng et al., 2022	0	73	0.00	[0.00; 0.05]	2.5%
Ng et al., 2022	20	684	0.03	[0.02; 0.04]	2.6%
Alsoof et al., 2022	27	792	0.03	[0.02; 0.05]	2.6%
White et al., 2023	5	63	0.08	[0.03; 0.18]	1.5%
Porche et al., 2023	0	250	0.00	[0.00; 0.01]	2.7%
D'Antonio et al., 2023	1	96	0.01	[0.00; 0.06]	2.5%
D'Antonio et al., 2023	1	32	0.03	[0.00; 0.16]	1.6%
D'Antonio et al., 2023	1	54	0.02	[0.00; 0.10]	2.2%
Chanbour et al., 2023	22	406	0.05	[0.03; 0.08]	2.5%
Tang et al., 2024	1	33	0.03	[0.00; 0.16]	1.7%
**Random effects model**		**13664**	**0.04**	**[0.02; 0.05]**	**100.0%**

Heterogeneity: $I^2 = 97\%$

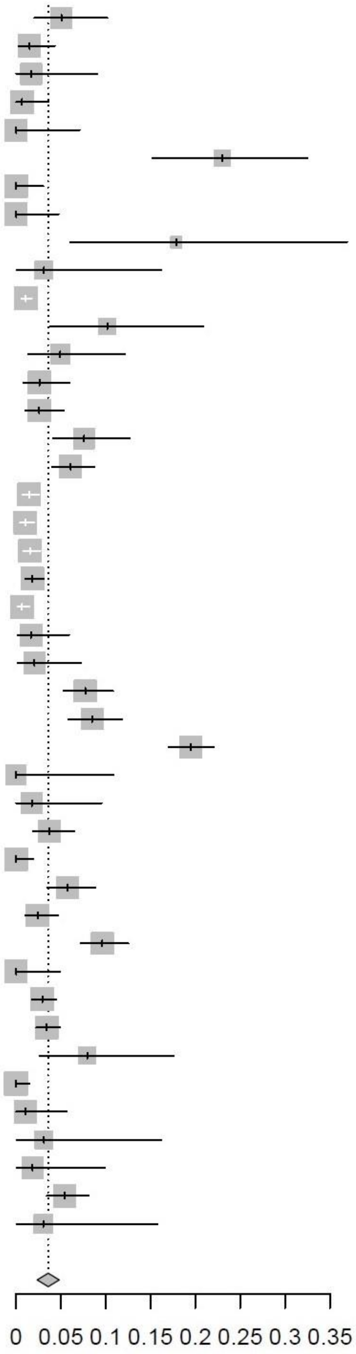

Supplementary Figure 30. Forest plot for mortality.

Study	Events	Total	Proportion	95%−CI	Weight
Nirala et al., 2003	1	69	0.01	[0.00; 0.08]	0.0%
Lied et al., 2006	0	390	0.00	[0.00; 0.01]	2.0%
Garringer et al., 2010	0	645	0.00	[0.00; 0.01]	5.6%
Zigler et al., 2012	3	106	0.03	[0.01; 0.08]	0.0%
Lied et al., 2012	0	96	0.00	[0.00; 0.04]	0.1%
Lovecchio et al., 2013	0	59	0.00	[0.00; 0.06]	0.0%
Tally et al., 2013	0	119	0.00	[0.00; 0.03]	0.2%
Martin et al., 2014	1	597	0.00	[0.00; 0.01]	2.4%
Bydon et al., 2014	0	391	0.00	[0.00; 0.01]	2.1%
Nanda et al., 2014	2	1576	0.00	[0.00; 0.00]	8.3%
Adamson et al., 2016	1	1478	0.00	[0.00; 0.00]	14.6%
Phan et al., 2017	0	270	0.00	[0.00; 0.01]	1.0%
Phan et al., 2017	1	171	0.01	[0.00; 0.03]	0.2%
Phan et al., 2017	4	444	0.01	[0.00; 0.02]	0.3%
Riederman et al., 2017	0	36	0.00	[0.00; 0.10]	0.0%
Riederman et al., 2017	0	96	0.00	[0.00; 0.04]	0.1%
Riederman et al., 2017	0	164	0.00	[0.00; 0.02]	0.4%
Riederman et al., 2017	0	104	0.00	[0.00; 0.03]	0.1%
Phan et al., 2017	2	760	0.00	[0.00; 0.01]	1.9%
Phan et al., 2017	3	760	0.00	[0.00; 0.01]	1.3%
Phan et al., 2017	1	760	0.00	[0.00; 0.01]	3.9%
Phan et al., 2017	4	760	0.01	[0.00; 0.01]	1.0%
Phan et al., 2017	0	761	0.00	[0.00; 0.00]	7.8%
Oni et al., 2018	0	85	0.00	[0.00; 0.04]	0.1%
Wang et al., 2019	0	113	0.00	[0.00; 0.03]	0.2%
Rossi et al., 2019	0	119	0.00	[0.00; 0.03]	0.2%
Rossi et al., 2019	0	97	0.00	[0.00; 0.04]	0.1%
Yang et al., 2019	0	49	0.00	[0.00; 0.07]	0.0%
Yang et al., 2019	0	58	0.00	[0.00; 0.06]	0.0%
Helseth et al., 2019	0	1300	0.00	[0.00; 0.00]	22.6%
Zaki et al., 2019	2	389	0.01	[0.00; 0.02]	0.5%
Shuman et al., 2020	2	365	0.01	[0.00; 0.02]	0.4%
Labaran et al., 2020	58	992	0.06	[0.04; 0.07]	0.1%
Neifert et al., 2020	0	821	0.00	[0.00; 0.00]	9.0%
Neifert et al., 2020	0	938	0.00	[0.00; 0.00]	11.8%
Wang et al., 2021	5	331	0.02	[0.00; 0.03]	0.1%
Wang et al., 2021	1	331	0.00	[0.00; 0.02]	0.7%
Nayak et al., 2022	3	507	0.01	[0.00; 0.02]	0.6%
Kitamura et al., 2022	1	90	0.01	[0.00; 0.06]	0.1%
Tang et al., 2024	0	33	0.00	[0.00; 0.11]	0.0%
**Random effects model**		**17230**	**0.00**	**[0.00; 0.00]**	**100.0%**

Heterogeneity: $I^2 = 0\%$

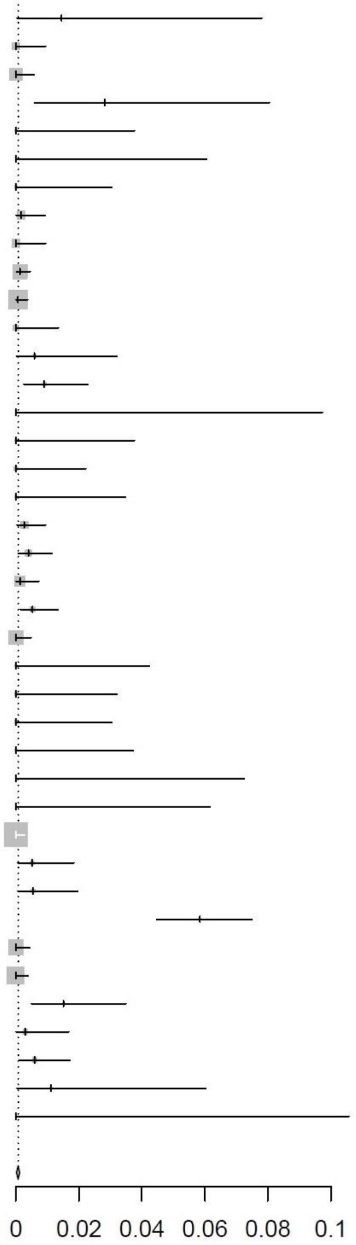

Supplementary Figure 31. Forest plot for overall complication rate.

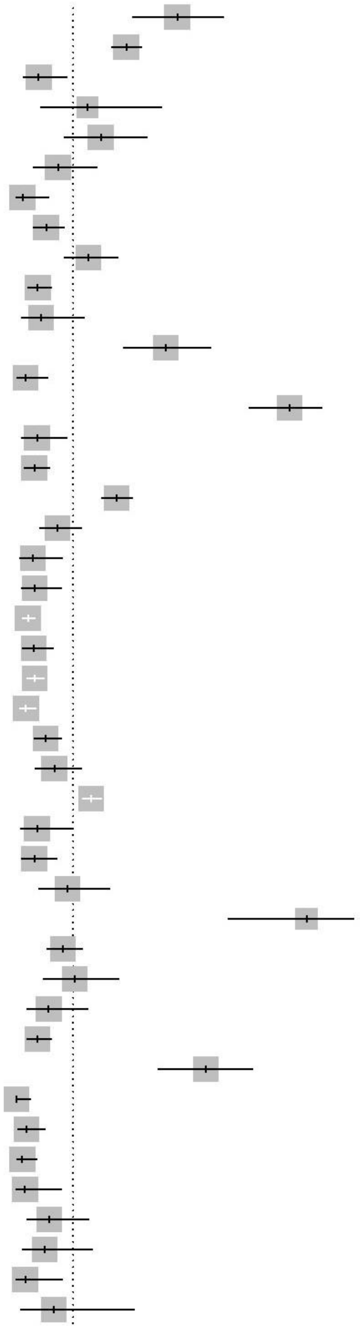

Study	Events	Total	Proportion	95%−CI	Weight
Swank et al., 1996	29	64	0.45	[0.33; 0.58]	0.4%
Cauthen et al., 1997	159	514	0.31	[0.27; 0.35]	0.4%
Wang et al., 1999	5	80	0.06	[0.02; 0.14]	0.4%
Wirth et al., 2000	5	25	0.20	[0.07; 0.41]	0.3%
Wang et al., 2000	14	59	0.24	[0.14; 0.37]	0.4%
Wang et al., 2000	7	60	0.12	[0.05; 0.23]	0.4%
Bruneau et al., 2001	1	60	0.02	[0.00; 0.09]	0.4%
Bose et al., 2001	15	180	0.08	[0.05; 0.13]	0.4%
Bazaz et al., 2002	25	123	0.20	[0.14; 0.29]	0.4%
Kaiser et al., 2002	15	251	0.06	[0.03; 0.10]	0.4%
Tureyen et al., 2003	3	43	0.07	[0.01; 0.19]	0.4%
Nirala et al., 2003	29	69	0.42	[0.30; 0.55]	0.4%
Samartzis et al., 2003	2	80	0.02	[0.00; 0.09]	0.4%
Yue et al., 2004	57	74	0.77	[0.66; 0.86]	0.4%
Samartzis et al., 2004	4	69	0.06	[0.02; 0.14]	0.4%
Cho et al., 2004	9	180	0.05	[0.02; 0.09]	0.4%
Bolesta et al., 2005	128	454	0.28	[0.24; 0.33]	0.4%
Shields et al., 2005	16	138	0.12	[0.07; 0.18]	0.4%
Samartzis et al., 2005	3	66	0.05	[0.01; 0.13]	0.4%
Hwang et al., 2005	4	78	0.05	[0.01; 0.13]	0.4%
Ikenaga et al., 2005	18	563	0.03	[0.02; 0.05]	0.4%
Korinth et al., 2006	6	124	0.05	[0.02; 0.10]	0.4%
Lied et al., 2006	20	390	0.05	[0.03; 0.08]	0.4%
Mummaneni et al., 2007	7	265	0.03	[0.01; 0.05]	0.4%
Mobbs et al., 2007	20	242	0.08	[0.05; 0.12]	0.4%
Villavicencio et al., 2007	11	103	0.11	[0.05; 0.18]	0.4%
Fountas et al., 2007	214	1015	0.21	[0.19; 0.24]	0.4%
Aryan et al., 2007	3	52	0.06	[0.01; 0.16]	0.4%
Ruetten et al., 2008	5	100	0.05	[0.02; 0.11]	0.4%
Chang−Jung et al., 2008	8	56	0.14	[0.06; 0.26]	0.4%
Dogan et al., 2008	18	22	0.82	[0.60; 0.95]	0.3%
Tumialán et al., 2008	26	200	0.13	[0.09; 0.18]	0.4%
Chou et al., 2008	9	55	0.16	[0.08; 0.29]	0.4%
Ghahreman et al., 2009	5	55	0.09	[0.03; 0.20]	0.4%
Heller et al., 2009	13	221	0.06	[0.03; 0.10]	0.4%
Kast et al., 2009	32	60	0.53	[0.40; 0.66]	0.4%
Chen et al., 2009	0	92	0.00	[0.00; 0.04]	0.4%
Liu et al., 2009	3	109	0.03	[0.01; 0.08]	0.4%
Leach et al., 2009	2	123	0.02	[0.00; 0.06]	0.4%
Uribe et al., 2009	1	42	0.02	[0.00; 0.13]	0.4%
Rohe et al., 2009	5	54	0.09	[0.03; 0.20]	0.4%
Scholz et al., 2010	3	38	0.08	[0.02; 0.21]	0.4%
Coric et al., 2010	1	41	0.02	[0.00; 0.13]	0.4%
Tumialán et al., 2010	2	19	0.11	[0.01; 0.33]	0.4%

Tumialán et al., 2010	2	19	0.11	[0.01; 0.33]	0.4%
Guo et al., 2010	1	43	0.02	[0.00; 0.12]	0.4%
Song et al., 2010	8	83	0.10	[0.04; 0.18]	0.4%
Garringer et al., 2010	2	645	0.00	[0.00; 0.01]	0.4%
Joseffer et al., 2010	79	390	0.20	[0.16; 0.25]	0.4%
Guo et al., 2010	8	53	0.15	[0.07; 0.28]	0.4%
Coric et al., 2011	6	133	0.05	[0.02; 0.10]	0.4%
Rihn et al., 2011	3	38	0.08	[0.02; 0.21]	0.4%
Lin et al., 2011	11	57	0.19	[0.10; 0.32]	0.4%
Iampreechakul et al., 2011	5	67	0.07	[0.02; 0.17]	0.4%
Sheperd et al., 2012	119	152	0.78	[0.71; 0.85]	0.4%
Feng et al., 2012	0	97	0.00	[0.00; 0.04]	0.4%
Coric et al., 2012	1	33	0.03	[0.00; 0.16]	0.4%
Song et al., 2012	8	25	0.32	[0.15; 0.54]	0.3%
Liu et al., 2012	16	103	0.16	[0.09; 0.24]	0.4%
Wu et al., 2012	16	67	0.24	[0.14; 0.36]	0.4%
Liu et al., 2012	15	69	0.22	[0.13; 0.33]	0.4%
Lied et al., 2012	5	96	0.05	[0.02; 0.12]	0.4%
Yamagata et al., 2012	11	47	0.23	[0.12; 0.38]	0.4%
Lovecchio et al., 2013	13	59	0.22	[0.12; 0.35]	0.4%
Burkhardt et al., 2013	7	93	0.08	[0.03; 0.15]	0.4%
Hofstetter et al., 2013	17	70	0.24	[0.15; 0.36]	0.4%
Lu et al., 2013	22	50	0.44	[0.30; 0.59]	0.4%
Lu et al., 2013	49	100	0.49	[0.39; 0.59]	0.4%
Davis et al., 2013	19	105	0.18	[0.11; 0.27]	0.4%
Phillips et al., 2013	21	185	0.11	[0.07; 0.17]	0.4%
Park et al., 2013	10	31	0.32	[0.17; 0.51]	0.3%
Landriel et al., 2013	1	30	0.03	[0.00; 0.17]	0.4%
Fang et al., 2013	5	54	0.09	[0.03; 0.20]	0.4%
Barbagallo et al., 2013	14	85	0.16	[0.09; 0.26]	0.4%
Wang et al., 2014	9	63	0.14	[0.07; 0.25]	0.4%
Martin et al., 2014	20	597	0.03	[0.02; 0.05]	0.4%
Tracey et al., 2014	31	88	0.35	[0.25; 0.46]	0.4%
Minhas et al., 2014	14	387	0.04	[0.02; 0.06]	0.4%
Bydon et al., 2014	13	391	0.03	[0.02; 0.06]	0.4%
Kasliwal et al., 2014	25	59	0.42	[0.30; 0.56]	0.4%
Kao et al., 2014	69	182	0.38	[0.31; 0.45]	0.4%
Yoo et al., 2014	33	58	0.57	[0.43; 0.70]	0.4%
Omidi−Kashani et al., 2014	19	74	0.26	[0.16; 0.37]	0.4%
Kim et al., 2014	6	134	0.04	[0.02; 0.09]	0.4%
Nanda et al., 2014	83	1576	0.05	[0.04; 0.06]	0.4%
Lee et al., 2014	36	78	0.46	[0.35; 0.58]	0.4%
Klingler et al., 2014	51	107	0.48	[0.38; 0.58]	0.4%
Yao et al., 2015	6	456	0.01	[0.00; 0.03]	0.4%
Chen et al., 2015	3	69	0.04	[0.01; 0.12]	0.4%
Elsamadicy et al., 2015	1	28	0.04	[0.00; 0.18]	0.4%
Elsamadicy et al., 2015	2	32	0.06	[0.01; 0.21]	0.4%

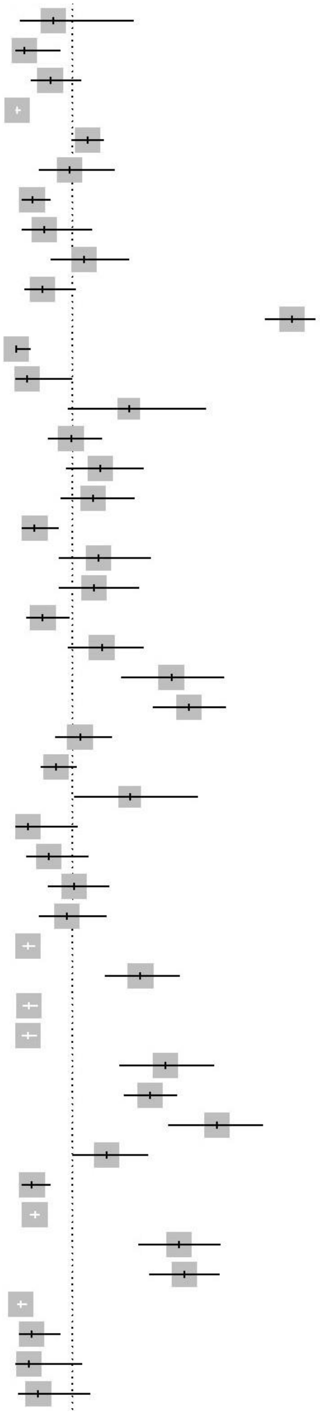

Liu et al., 2015	4	22	0.18	[0.05; 0.40]	0.3%
Wang et al., 2015	6	30	0.20	[0.08; 0.39]	0.3%
Wang et al., 2015	1	27	0.04	[0.00; 0.19]	0.4%
Shiban et al., 2015	87	265	0.33	[0.27; 0.39]	0.4%
Liu et al., 2016	8	60	0.13	[0.06; 0.25]	0.4%
Chen et al., 2016	8	54	0.15	[0.07; 0.27]	0.4%
De La Garza−Ramos et al., 2016	51	97	0.53	[0.42; 0.63]	0.4%
Wang et al., 2016	0	52	0.00	[0.00; 0.07]	0.4%
Shi et al., 2016	27	65	0.42	[0.29; 0.54]	0.4%
Adamson et al., 2016	46	1478	0.03	[0.02; 0.04]	0.4%
Ahn et al., 2016	1	68	0.01	[0.00; 0.08]	0.4%
Fu et al., 2016	7	65	0.11	[0.04; 0.21]	0.4%
Radcliff et al., 2016	3	25	0.12	[0.03; 0.31]	0.4%
Adogwa et al., 2016	3	59	0.05	[0.01; 0.14]	0.4%
Adogwa et al., 2016	5	81	0.06	[0.02; 0.14]	0.4%
Chin et al., 2016	4	55	0.07	[0.02; 0.18]	0.4%
Lovasik et al., 2016	53	191	0.28	[0.22; 0.35]	0.4%
Shi et al., 2016	4	57	0.07	[0.02; 0.17]	0.4%
Shi et al., 2016	8	68	0.12	[0.05; 0.22]	0.4%
Yang et al., 2016	14	139	0.10	[0.06; 0.16]	0.4%
Alimi et al., 2016	8	104	0.08	[0.03; 0.15]	0.4%
ElAbed et al., 2016	8	90	0.09	[0.04; 0.17]	0.4%
Yamagata et al., 2016	8	45	0.18	[0.08; 0.32]	0.4%
Yamagata et al., 2016	11	55	0.20	[0.10; 0.33]	0.4%
Qizhi et al., 2016	4	16	0.25	[0.07; 0.52]	0.3%
Ahn et al., 2016	5	32	0.16	[0.05; 0.33]	0.4%
Sang et al., 2016	4	32	0.12	[0.04; 0.29]	0.4%
Phan et al., 2017	17	270	0.06	[0.04; 0.10]	0.4%
Phan et al., 2017	4	171	0.02	[0.01; 0.06]	0.4%
Li et al., 2017	8	31	0.26	[0.12; 0.45]	0.3%
Fisahn et al., 2017	14	211	0.07	[0.04; 0.11]	0.4%
Fisahn et al., 2017	20	166	0.12	[0.08; 0.18]	0.4%
Phan et al., 2017	31	444	0.07	[0.05; 0.10]	0.4%
Riederman et al., 2017	10	36	0.28	[0.14; 0.45]	0.3%
Riederman et al., 2017	22	96	0.23	[0.15; 0.33]	0.4%
Riederman et al., 2017	46	164	0.28	[0.21; 0.36]	0.4%
Riederman et al., 2017	34	104	0.33	[0.24; 0.43]	0.4%
Choi et al., 2017	24	84	0.29	[0.19; 0.39]	0.4%
Bucci et al., 2017	3	110	0.03	[0.01; 0.08]	0.4%
Mullins et al., 2017	45	1123	0.04	[0.03; 0.05]	0.4%
Phan et al., 2017	18	760	0.02	[0.01; 0.04]	0.4%
Phan et al., 2017	19	760	0.02	[0.02; 0.04]	0.4%
Phan et al., 2017	23	760	0.03	[0.02; 0.05]	0.4%
Phan et al., 2017	64	760	0.08	[0.07; 0.11]	0.4%
Phan et al., 2017	12	761	0.02	[0.01; 0.03]	0.4%

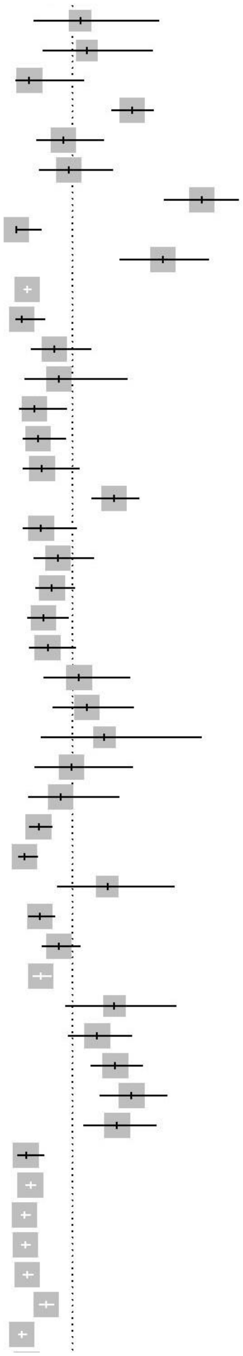

Yagi et al., 2017	9	307	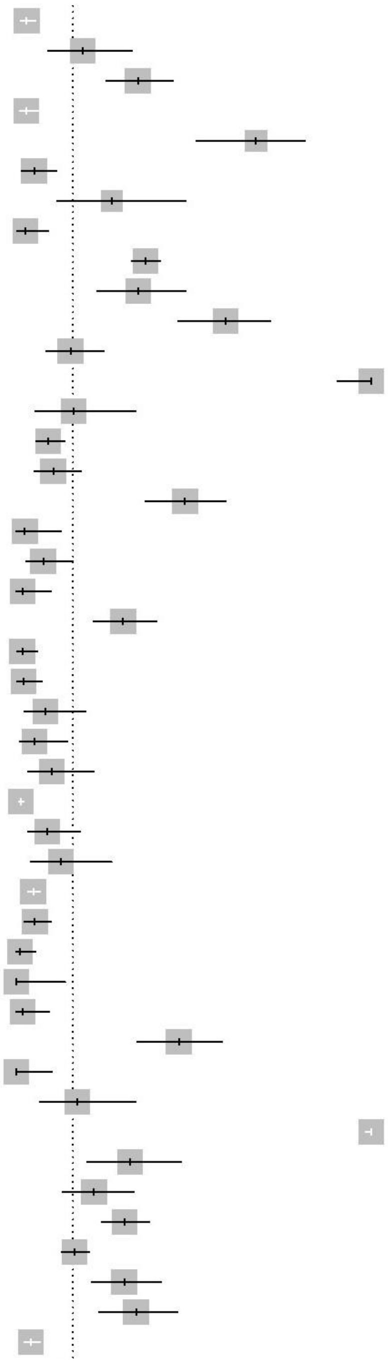	0.03	[0.01; 0.05]	0.4%
Jack et al., 2018	9	48		0.19	[0.09; 0.33]	0.4%
Lee et al., 2018	36	105		0.34	[0.25; 0.44]	0.4%
Dunn et al., 2018	6	210		0.03	[0.01; 0.06]	0.4%
Scholz et al., 2018	27	40		0.68	[0.51; 0.81]	0.3%
Grasso et al., 2018	5	100		0.05	[0.02; 0.11]	0.4%
Wang et al., 2018	7	26		0.27	[0.12; 0.48]	0.3%
Zhang et al., 2018	2	77		0.03	[0.00; 0.09]	0.4%
Staartjes et al., 2018	201	551		0.36	[0.32; 0.41]	0.4%
Chughtai et al., 2018	21	61		0.34	[0.23; 0.48]	0.4%
Chughtai et al., 2018	36	61		0.59	[0.46; 0.71]	0.4%
Oni et al., 2018	13	85		0.15	[0.08; 0.25]	0.4%
Kim et al., 2018	37	37		1.00	[0.91; 1.00]	0.4%
Kim et al., 2018	5	31		0.16	[0.05; 0.34]	0.4%
Huschbeck et al., 2019	19	211		0.09	[0.06; 0.14]	0.4%
Bivo et al., 2019	10	96		0.10	[0.05; 0.18]	0.4%
Yan et al., 2019	39	82		0.48	[0.36; 0.59]	0.4%
Labaran et al., 2019	1	42		0.02	[0.00; 0.13]	0.4%
Gandhi et al., 2019	6	79		0.08	[0.03; 0.16]	0.4%
Lin et al., 2019	1	55		0.02	[0.00; 0.10]	0.4%
Wang et al., 2019	34	113		0.30	[0.22; 0.39]	0.4%
Rossi et al., 2019	2	119		0.02	[0.00; 0.06]	0.4%
Rossi et al., 2019	2	97		0.02	[0.00; 0.07]	0.4%
Yang et al., 2019	4	49		0.08	[0.02; 0.20]	0.4%
Yang et al., 2019	3	58		0.05	[0.01; 0.14]	0.4%
Chang et al., 2019	5	50		0.10	[0.03; 0.22]	0.4%
Helseth et al., 2019	16	1300		0.01	[0.01; 0.02]	0.4%
Yeung et al., 2019	6	69		0.09	[0.03; 0.18]	0.4%
Xu et al., 2019	5	40		0.12	[0.04; 0.27]	0.4%
Malik et al., 2019	31	649		0.05	[0.03; 0.07]	0.4%
Patel et al., 2019	9	172		0.05	[0.02; 0.10]	0.4%
Patel et al., 2019	1	100		0.01	[0.00; 0.05]	0.4%
Vaishnav et al., 2019	0	25		0.00	[0.00; 0.14]	0.4%
Vaishv et al., 2019	1	58		0.02	[0.00; 0.09]	0.4%
Wewel et al., 2019	33	72		0.46	[0.34; 0.58]	0.4%
Grasso et al., 2019	0	35		0.00	[0.00; 0.10]	0.4%
Grasso et al., 2019	6	35		0.17	[0.07; 0.34]	0.4%
Aguilara et al., 2019	239	239		1.00	[0.98; 1.00]	0.4%
De Leo–Vargas et al., 2019	17	53		0.32	[0.20; 0.46]	0.4%
Opsenak et al., 2019	16	73		0.22	[0.13; 0.33]	0.4%
Perdomo−Pantoja et al., 2019	61	200		0.30	[0.24; 0.37]	0.4%
Zaki et al., 2019	64	389		0.16	[0.13; 0.21]	0.4%
Jang et al., 2020	28	92		0.30	[0.21; 0.41]	0.4%
Maccormick et al., 2020	26	77		0.34	[0.23; 0.45]	0.4%
Shuman et al., 2020	15	365		0.04	[0.02; 0.07]	0.4%

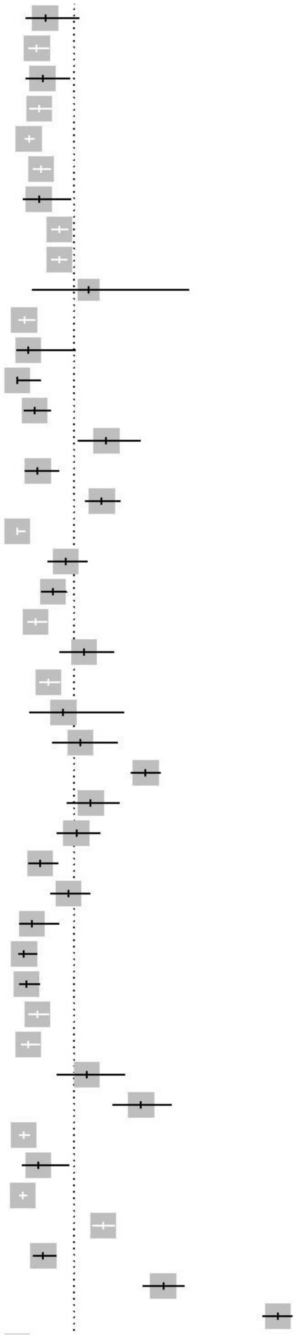

Ahn et al., 2020	5	64	0.08	[0.03; 0.17]	0.4%
Narain et al., 2020	17	310	0.05	[0.03; 0.09]	0.4%
Lee et al., 2020	6	85	0.07	[0.03; 0.15]	0.4%
Ren et al., 2020	18	295	0.06	[0.04; 0.09]	0.4%
Labaran et al., 2020	32	992	0.03	[0.02; 0.05]	0.4%
An et al., 2020	32	484	0.07	[0.05; 0.09]	0.4%
Guo et al., 2020	4	66	0.06	[0.02; 0.15]	0.4%
Neifert et al., 2020	97	821	0.12	[0.10; 0.14]	0.4%
Neifert et al., 2020	110	938	0.12	[0.10; 0.14]	0.4%
Houten et al., 2020	3	15	0.20	[0.04; 0.48]	0.3%
Huang et al., 2021	4	208	0.02	[0.01; 0.05]	0.4%
Yang et al., 2021	1	32	0.03	[0.00; 0.16]	0.4%
Yang et al., 2021	0	56	0.00	[0.00; 0.06]	0.4%
Sommaruga et al., 2021	8	166	0.05	[0.02; 0.09]	0.4%
Gowd et al., 2021	27	108	0.25	[0.17; 0.34]	0.4%
Sinensky et al., 2021	7	122	0.06	[0.02; 0.11]	0.4%
Fayed et al., 2021	76	321	0.24	[0.19; 0.29]	0.4%
Khalifeh et al., 2021	0	190	0.00	[0.00; 0.02]	0.4%
Nguyen et al., 2021	23	170	0.14	[0.09; 0.20]	0.4%
Wang et al., 2021	33	331	0.10	[0.07; 0.14]	0.4%
Wang et al., 2021	17	331	0.05	[0.03; 0.08]	0.4%
Sheng et al., 2021	22	118	0.19	[0.12; 0.27]	0.4%
Wong et al., 2021	41	469	0.09	[0.06; 0.12]	0.4%
Niljianskul et al., 2021	4	31	0.13	[0.04; 0.30]	0.4%
Pinter et al., 2022	14	79	0.18	[0.10; 0.28]	0.4%
Lambrechts et al., 2022	215	597	0.36	[0.32; 0.40]	0.4%
Kaufman et al., 2022	27	131	0.21	[0.14; 0.29]	0.4%
Wei et al., 2022	27	163	0.17	[0.11; 0.23]	0.4%
Wei et al., 2022	11	171	0.06	[0.03; 0.11]	0.4%
Wei et al., 2022	26	182	0.14	[0.10; 0.20]	0.4%
Leng et al., 2022	3	73	0.04	[0.01; 0.12]	0.4%
Patel et al., 2022	3	161	0.02	[0.00; 0.05]	0.4%
Patel et al., 2022	4	161	0.02	[0.01; 0.06]	0.4%
Patel et al., 2022	18	320	0.06	[0.03; 0.09]	0.4%
Padhye et al., 2022	8	257	0.03	[0.01; 0.06]	0.4%
Mu et al., 2022	15	77	0.19	[0.11; 0.30]	0.4%
Cao et al., 2022	50	144	0.35	[0.27; 0.43]	0.4%
Schuermans et al., 2022	10	548	0.02	[0.01; 0.03]	0.4%
Ma et al., 2022	4	68	0.06	[0.02; 0.14]	0.4%
Ng et al., 2022	10	684	0.01	[0.01; 0.03]	0.4%
Alsoof et al., 2022	191	792	0.24	[0.21; 0.27]	0.4%
Hao et al., 2022	22	302	0.07	[0.05; 0.11]	0.4%
Lambrechts et al., 2022	122	298	0.41	[0.35; 0.47]	0.4%
Nayak et al., 2022	371	507	0.73	[0.69; 0.77]	0.4%

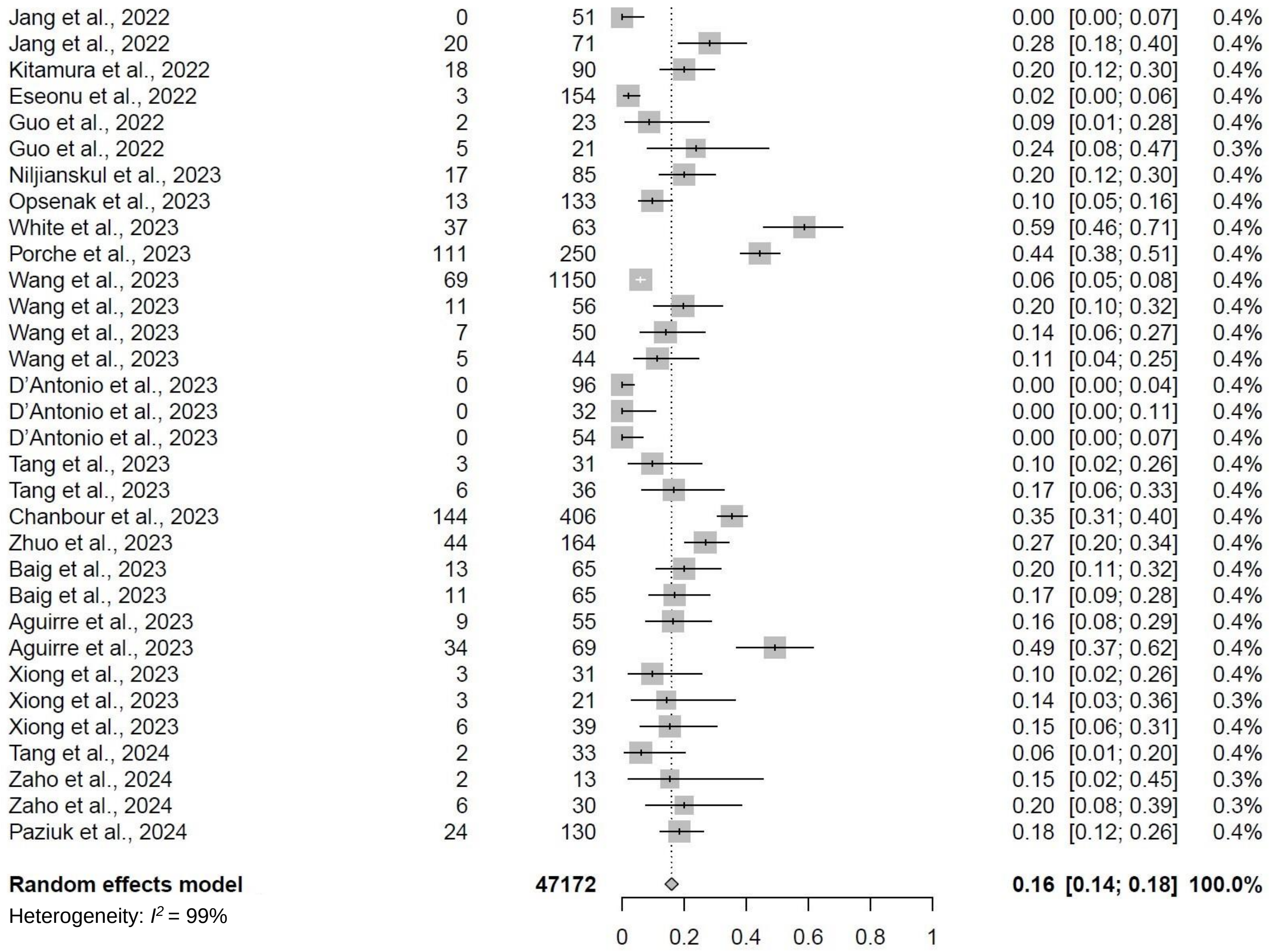
Jang et al., 2022 0 51 0.00 [0.00; 0.07] 0.4%
Jang et al., 2022 20 71 0.28 [0.18; 0.40] 0.4%
Kitamura et al., 2022 18 90 0.20 [0.12; 0.30] 0.4%
Eseonu et al., 2022 3 154 0.02 [0.00; 0.06] 0.4%
Guo et al., 2022 2 23 0.09 [0.01; 0.28] 0.4%
Guo et al., 2022 5 21 0.24 [0.08; 0.47] 0.3%
Niljianskul et al., 2023 17 85 0.20 [0.12; 0.30] 0.4%
Opsenak et al., 2023 13 133 0.10 [0.05; 0.16] 0.4%
White et al., 2023 37 63 0.59 [0.46; 0.71] 0.4%
Porche et al., 2023 111 250 0.44 [0.38; 0.51] 0.4%
Wang et al., 2023 69 1150 0.06 [0.05; 0.08] 0.4%
Wang et al., 2023 11 56 0.20 [0.10; 0.32] 0.4%
Wang et al., 2023 7 50 0.14 [0.06; 0.27] 0.4%
Wang et al., 2023 5 44 0.11 [0.04; 0.25] 0.4%
D’Antonio et al., 2023 0 96 0.00 [0.00; 0.04] 0.4%
D’Antonio et al., 2023 0 32 0.00 [0.00; 0.11] 0.4%
D’Antonio et al., 2023 0 54 0.00 [0.00; 0.07] 0.4%
Tang et al., 2023 3 31 0.10 [0.02; 0.26] 0.4%
Tang et al., 2023 6 36 0.17 [0.06; 0.33] 0.4%
Chanbour et al., 2023 144 406 0.35 [0.31; 0.40] 0.4%
Zhuo et al., 2023 44 164 0.27 [0.20; 0.34] 0.4%
Baig et al., 2023 13 65 0.20 [0.11; 0.32] 0.4%
Baig et al., 2023 11 65 0.17 [0.09; 0.28] 0.4%
Aguirre et al., 2023 9 55 0.16 [0.08; 0.29] 0.4%
Aguirre et al., 2023 34 69 0.49 [0.37; 0.62] 0.4%
Xiong et al., 2023 3 31 0.10 [0.02; 0.26] 0.4%
Xiong et al., 2023 3 21 0.14 [0.03; 0.36] 0.3%
Xiong et al., 2023 6 39 0.15 [0.06; 0.31] 0.4%
Tang et al., 2024 2 33 0.06 [0.01; 0.20] 0.4%
Zaho et al., 2024 2 13 0.15 [0.02; 0.45] 0.3%
Zaho et al., 2024 6 30 0.20 [0.08; 0.39] 0.3%
Paziuk et al., 2024 24 130 0.18 [0.12; 0.26] 0.4%
Random effects model 47172 0.16 [0.14; 0.18] 100.0%
Heterogeneity: I2 = 99%
0 0.2 0.4 0.6 0.8 1

References included in the meta-analysis

1. Adamson T, Godil SS, Mehrlich M, Mendenhall S, Asher AL, McGirt MJ. Anterior cervical discectomy and fusion in the outpatient ambulatory surgery setting compared with the inpatient hospital setting: analysis of 1000 consecutive cases. SPI. 2016;24(6):878-884. doi:10.3171/2015.8.SPINE14284

2. Fountas KN, Kapsalaki EZ, Nikolakakos LG, et al. Anterior Cervical Discectomy and Fusion Associated Complications: Spine. 2007;32(21):2310-2317. doi:10.1097/BRS.0b013e318154c57e

3. Zaki O, Jain N, Yu EM, Khan SN. 30- and 90-Day Unplanned Readmission Rates, Causes, and Risk Factors After Cervical Fusion: A Single-Institution Analysis. Spine. 2019;44(11):762-769. doi:10.1097/BRS.0000000000002937

4. Burkhardt JK, Mannion AF, Marbacher S, et al. A comparative effectiveness study of patient-rated and radiographic outcome after 2 types of decompression with fusion for spondylotic myelopathy: anterior cervical discectomy versus corpectomy. FOC. 2013;35(1):E4. doi:10.3171/2013.3.FOCUS1396

5. Lin Q, Zhou X, Wang X, Cao P, Tsai N, Yuan W. A comparison of anterior cervical discectomy and corpectomy in patients with multilevel cervical spondylotic myelopathy. Eur Spine J. 2012;21(3):474-481. doi:10.1007/s00586-011-1961-9

6. Chen Y, Lü G, Wang B, Li L, Kuang L. A comparison of anterior cervical discectomy and fusion (ACDF) using self-locking stand-alone polyetheretherketone (PEEK) cage with ACDF using cage and plate in the treatment of three-level cervical degenerative spondylopathy: a retrospective study with 2-year follow-up. Eur Spine J. 2016;25(7):2255-2262. doi:10.1007/s00586-016-4391-x

7. Li Z, Huang J, Zhang Z, Li F, Hou T, Hou S. A Comparison of Multilevel Anterior Cervical Discectomy and Corpectomy in Patients With 4-level Cervical Spondylotic Myelopathy: a Minimum 2-year Follow-up Study: Multilevel Anterior Cervical Discectomy. Clinical Spine Surgery: A Spine Publication. 2017;30(5):E540-E546. doi:10.1097/BSD.0000000000000212

8. Qizhi S, Lei S, Peijia L, et al. A Comparison of Zero-Profile Devices and Artificial Cervical Disks in Patients With 2 Noncontiguous Levels of Cervical Spondylosis. Clinical Spine Surgery: A Spine Publication. 2016;29(2):E61-E66. doi:10.1097/BSD.0000000000000096

9. Yao Q, Liang F, Xia Y, Jia C. A meta-analysis comparing total disc arthroplasty with anterior cervical discectomy and fusion for the treatment of cervical degenerative diseases. Arch Orthop Trauma Surg. 2016;136(3):297-304. doi:10.1007/s00402-015-2337-0

10. Yamagata T, Naito K, Arima H, Yoshimura M, Ohata K, Takami T. A minimum 2-year comparative study of autologous cancellous bone grafting versus beta-tricalcium phosphate in anterior cervical discectomy and fusion using a rectangular titanium stand-alone cage. Neurosurg Rev. 2016;39(3):475-482. doi:10.1007/s10143-016-0714-y

11. Scholz M, Schnake KJ, Pingel A, Hoffmann R, Kandziora F. A New Zero-profile Implant for Stand-alone Anterior Cervical Interbody Fusion. Clinical Orthopaedics & Related Research. 2011;469(3):666-673. doi:10.1007/s11999-010-1597-9

12. Phillips FM, Lee JYB, Geisler FH, et al. A Prospective, Randomized, Controlled Clinical Investigation Comparing PCM Cervical Disc Arthroplasty With Anterior Cervical Discectomy and Fusion: 2-Year Results From the US FDA IDE Clinical Trial. Spine. 2013;38(15):E907-E918. doi:10.1097/BRS.0b013e318296232f

13. Perdomo-Pantoja A, Shamoun F, Holmes C, et al. A retrospective cohort analysis of the effects of renin-angiotensin system inhibitors on spinal fusion in ACDF patients. The Spine Journal. 2019;19(8):1354-1361. doi:10.1016/j.spinee.2019.04.017

14. Khalifeh K, Faulkner JE, Hara J, Ozgur B. A Retrospective Evaluation and Review of Outcomes for Single- and Multilevel ACDF With a Zero-Profile Stand-Alone Cage Device With Integrated Instrumentation. Cureus. Published online April 3, 2021. doi:10.7759/cureus.14283

15. Nirala A, Husain M, Vatsal D. A retrospective study of multiple interbody grafting and long segment strut grafting following multilevel anterior cervical decompression. British Journal of Neurosurgery. 2004;18(3):227-232. doi:10.1080/02688690410001732643

16. Shields LBE, Raque GH, Glassman SD, et al. Adverse Effects Associated With High-Dose Recombinant Human Bone Morphogenetic Protein-2 Use in Anterior Cervical Spine Fusion: Spine. 2006;31(5):542-547. doi:10.1097/01.brs.0000201424.27509.72

17. Guo H, Sheng J, Sheng W, Liang W, Wang J, Xun C. An Eight-Year Follow-Up Study on the Treatment of Single-Level Cervical Spondylosis Through Intervertebral Disc Replacement and Anterior Cervical Decompression and Fusion. Orthop Surg. 2020;12(3):717-726. doi:10.1111/os.12634

18. Leng X, Zhang Y, Wang G, et al. An enhanced recovery after surgery pathway: LOS reduction, rapid discharge and minimal complications after anterior cervical spine surgery. BMC Musculoskelet Disord. 2022;23(1):252. doi:10.1186/s12891-022-05185-0

19. Maccormick AP, Sharma H. Analysis of the Variables Affecting the Incidence, Location, and Severity of Cage Subsidence Following Anterior Cervical Discectomy and Fusion Operation. Int J Spine Surg. 2020;14(6):896-900. doi:10.14444/7137

20. Phan K, Kim JS, Kim JH, et al. Anesthesia Duration as an Independent Risk Factor for Early Postoperative Complications in Adults Undergoing Elective ACDF. Global Spine Journal. 2017;7(8):727-734. doi:10.1177/2192568217701105

21. Swank ML, Lowery GL, Bhat AL, McDonough RF. Anterior cervical allograft arthrodesis and instrumentation: Multilevel interbody grafting or strut graft reconstruction. Eur Spine J. 1997;6(2):138-143. doi:10.1007/BF01358747

22. Scholz T, Geiger M, Mainz V, et al. Anterior Cervical Decompression and Fusion or Posterior Foraminotomy for Cervical Radiculopathy: Results of a Single-Center Series. J Neurol Surg A Cent Eur Neurosurg. 2018;79(03):211-217. doi:10.1055/s-0037-1607225

23. Alimi M, Njoku I, Hofstetter CP, et al. Anterior Cervical Discectomy and Fusion (ACDF): Comparison Between Zero Profile Implants and Anterior Cervical Plate and Spacer. Cureus. Published online April 17, 2016. doi:10.7759/cureus.573

24. Yeung KKL, Cheung PWH, Cheung JPY. Anterior cervical discectomy and fusion for cervical myelopathy using stand-alone tricortical iliac crest autograft: Predictive factors for neurological and fusion outcomes. J Orthop Surg (Hong Kong). 2019;27(3):230949901986916. doi:10.1177/2309499019869166

25. Wang M, Chou D, Chang CC, et al. Anterior cervical discectomy and fusion performed using structural allograft or polyetheretherketone: pseudarthrosis and revision surgery rates with minimum 2-year follow-up. Journal of Neurosurgery: Spine. 2020;32(4):562-569. doi:10.3171/2019.9.SPINE19879

26. Park JI, Cho DC, Kim KT, Sung JK. Anterior Cervical Discectomy and Fusion Using a Stand-Alone Polyetheretherketone Cage Packed with Local Autobone : Assessment of Bone Fusion and Subsidence. J Korean Neurosurg Soc. 2013;54(3):189. doi:10.3340/jkns.2013.54.3.189

27. Liu J, Chen X, Liu Z, Long X, Huang S, Shu Y. Anterior cervical discectomy and fusion versus corpectomy and fusion in treating two-level adjacent cervical spondylotic myelopathy: a minimum 5-year follow-up study. Arch Orthop Trauma Surg. 2015;135(2):149-153. doi:10.1007/s00402-014-2123-4

28. Xu Z, Rao H, Zhang L, Li G, Xu Z, Xu W. Anterior Cervical Discectomy and Fusion Versus Hybrid Decompression and Fusion for the Treatment of 3-Level Cervical Spondylotic Myelopathy: A Comparative Analysis of Cervical Sagittal Balance and Outcomes. World Neurosurgery. 2019;132:e752-e758. doi:10.1016/j.wneu.2019.08.022

29. Wang B, Lü G, Kuang L. Anterior cervical discectomy and fusion with stand-alone anchored cages versus posterior laminectomy and fusion for four-level cervical spondylotic myelopathy: a retrospective study with 2-year follow-up. BMC Musculoskelet Disord. 2018;19(1):216. doi:10.1186/s12891-018-2136-1

30. ElAbed K, Shawky A, Barakat M, Ainscow D. Anterior Cervical Discectomy and Fusion with Stand-Alone Trabecular Metal Cages as a Surgical Treatment for Cervical Radiculopathy: Mid-Term Outcomes. Asian Spine J. 2016;10(2):245. doi:10.4184/asj.2016.10.2.245

31. Mu G, Chen H, Fu H, et al. Anterior cervical discectomy and fusion with zero-profile versus stand-alone cages for two-level cervical spondylosis: A retrospective cohort study. Front Surg. 2022;9:1002744. doi:10.3389/fsurg.2022.1002744

32. Mobbs RJ, Rao P, Chandran NK. Anterior cervical discectomy and fusion: analysis of surgical outcome with and without plating. Journal of Clinical Neuroscience. 2007;14(7):639-642. doi:10.1016/j.jocn.2006.04.003

33. Lovasik BP, Holland CM, Howard BM, Baum GR, Rodts GE, Refai D. Anterior Cervical Discectomy and Fusion: Comparison of Fusion, Dysphagia, and Complication Rates Between Recombinant Human Bone Morphogenetic Protein-2 and Beta-Tricalcium Phosphate. World Neurosurgery. 2017;97:674-683.e1. doi:10.1016/j.wneu.2016.10.088

34. Rohe SM, Engelhardt M, Harders A, Schmieder K. Anterior Cervical Discectomy and Titanium Cage Fusion – 7-year Follow-up. Cen Eur Neurosurg. 2009;70(04):180-186. doi:10.1055/s-0029-1220940

35. Chang-Jung C, Yi-Jie K, Yueh-feng C, Rau G, Yang-Hwei T. Anterior Cervical Fusion Using a Polyetheretherketone Cage Containing a Bovine Xenograftp: Three to Five-Year Follow-up. Spine. 2008;33(23):2524-2428. doi:10.1097/BRS.0b013e318185289c

36. Bose B. Anterior Cervical Instrumentation Enhances Fusion Rates in Multilevel Reconstruction in Smokers: Journal of Spinal Disorders. 2001;14(1):3-9. doi:10.1097/00002517-200102000-00002

37. Bruneau M, Nisolle JF, Gilliard C, Gustin T. Anterior cervical interbody fusion with hydroxyapatite graft and plate system. FOC. 2001;10(4):1-6. doi:10.3171/foc.2001.10.4.9

38. Chen Y, Chen H, Cao P, Yuan W. Anterior cervical interbody fusion with the Zero-P spacer: mid-term results of two-level fusion. Eur Spine J. 2015;24(8):1666-1672. doi:10.1007/s00586-015-3919-9

39. Kaiser MG, Haid RW, Subach BR, Barnes B, Rodts GE. Anterior Cervical Plating Enhances Arthrodesis after Discectomy and Fusion with Cortical Allograft. Neurosurgery. 2002;50(2):229-238. doi:10.1097/00006123-200202000-00001

40. Guo Q, Ni B, Zhou F, et al. Anterior hybrid decompression and segmental fixation for adjacent three-level cervical spondylosis. Arch Orthop Trauma Surg. 2011;131(5):631-636. doi:10.1007/s00402-010-1181-5

41. Aryan HE, Lu DC, Acosta FL, Hartl R, McCormick PW, Ames CP. Bioabsorbable Anterior Cervical Plating: Initial Multicenter Clinical and Radiographic Experience. Spine. 2007;32(10):1084-1088. doi:10.1097/01.brs.0000261489.66229.c1

42. Leach J, Bittar RG. BMP-7 (OP-1®) Safety in anterior cervical fusion surgery. Journal of Clinical Neuroscience. 2009;16(11):1417-1420. doi:10.1016/j.jocn.2009.02.012

43. Wu WJ, Jiang LS, Liang Y, Dai LY. Cage subsidence does not, but cervical lordosis improvement does affect the long-term results of anterior cervical fusion with stand-alone cage for degenerative cervical disc disease: a retrospective study. Eur Spine J. 2012;21(7):1374-1382. doi:10.1007/s00586-011-2131-9

44. Wirth FP, Dowd GC, Sanders HF, Wirth C. Cervical discectomy. Surgical Neurology. 2000;53(4):340-348. doi:10.1016/S0090-3019(00)00201-9

45. Davis RJ, Kim KD, Hisey MS, et al. Cervical total disc replacement with the Mobi-C cervical artificial disc compared with anterior discectomy and fusion for treatment of 2-level symptomatic degenerative disc disease: a prospective, randomized, controlled multicenter clinical trial: Clinical article. SPI. 2013;19(5):532-545. doi:10.3171/2013.6.SPINE12527

46. Radcliff KE, Bennett J, Stewart RJ, et al. Change in Angular Alignment Is Associated With Early Dysphagia After Anterior Cervical Discectomy and Fusion. Clinical Spine Surgery: A Spine Publication. 2016;29(6):248-254. doi:10.1097/BSD.0b013e31828b39b4

47. Malik AT, Jain N, Kim J, Khan SN, Yu E. Chronic Obstructive Pulmonary Disease Is an Independent Predictor for 30-Day Complications and Readmissions Following 1- to 2-Level Anterior Cervical Discectomy and Fusion. Global Spine Journal. 2019;9(3):298-302. doi:10.1177/2192568218794170

48. Schuermans VNE, Smeets AYJM, Wijsen NPMH, Curfs I, Boselie TFM, Van Santbrink H. Clinical adjacent segment pathology after anterior cervical discectomy, with and without fusion, for cervical degenerative disc disease: A single center retrospective cohort study with long-term follow-up. Brain and Spine. 2022;2:100869. doi:10.1016/j.bas.2022.100869

49. Kim S, Lee SH, Kim ES, Eoh W. Clinical and Radiographic Analysis of C5 Palsy After Anterior Cervical Decompression and Fusion for Cervical Degenerative Disease. Journal of Spinal Disorders & Techniques. 2014;27(8):436-441. doi:10.1097/BSD.0b013e31826a10b0

50. Mummaneni PV, Burkus JK, Haid RW, Traynelis VC, Zdeblick TA. Clinical and radiographic analysis of cervical disc arthroplasty compared with allograft fusion: a randomized controlled clinical trial. SPI. 2007;6(3):198-209. doi:10.3171/spi.2007.6.3.198

51. Dogan S, Turkkan A, Kocaeli H, Korfali E, Bekar A. Clinical and Radiologic Analysis of 3-level Anterior Cervical Discectomy and Fusion With Interbody Cages Without Plate Fixation: Neurosurgery Quarterly. 2008;18(3):188-194. doi:10.1097/WNQ.0b013e3181820a58

52. Shiban E, Gapon K, Wostrack M, Meyer B, Lehmberg J. Clinical and radiological outcome after anterior cervical discectomy and fusion with stand-alone empty polyetheretherketone (PEEK) cages. Acta Neurochir. 2016;158(2):349-355. doi:10.1007/s00701-015-2630-2

53. Yang S, Yu Y, Liu X, et al. Clinical and radiological results comparison of allograft and polyetheretherketone cage for one to two-level anterior cervical discectomy and fusion: A CONSORT-compliant article. Medicine. 2019;98(45):e17935. doi:10.1097/MD.0000000000017935

54. Kasliwal MK, O'Toole JE. Clinical experience using polyetheretherketone (PEEK) intervertebral structural cage for anterior cervical corpectomy and fusion. Journal of Clinical Neuroscience. 2014;21(2):217-220. doi:10.1016/j.jocn.2013.03.018

55. Sommaruga S, Camara-Quintana J, Patel K, et al. Clinical Outcomes between Stand-Alone Zero-Profile Spacers and Cervical Plate with Cage Fixation for Anterior Cervical Discectomy and Fusion: A Retrospective Analysis of 166 Patients. JCM. 2021;10(14):3076. doi:10.3390/jcm10143076

56. Chin KR, Pencle FJR, Seale JA, Pencle FK. Clinical Outcomes of Outpatient Cervical Total Disc Replacement Compared With Outpatient Anterior Cervical Discectomy and Fusion. Spine. 2017;42(10):E567-E574. doi:10.1097/BRS.0000000000001936

57. Patel DV, Yoo JS, Haws BE, et al. Comparative analysis of anterior cervical discectomy and fusion in the inpatient versus outpatient surgical setting. Journal of Neurosurgery: Spine. 2019;31(2):255-260. doi:10.3171/2019.1.SPINE181311

58. Liu Y, Qi M, Chen H, et al. Comparative analysis of complications of different reconstructive techniques following anterior decompression for multilevel cervical spondylotic myelopathy. Eur Spine J. 2012;21(12):2428-2435. doi:10.1007/s00586-012-2323-y

59. Uribe JS, Sangala JR, Duckworth EAM, Vale FL. Comparison between anterior cervical discectomy fusion and cervical corpectomy fusion using titanium cages for reconstruction: analysis of outcome and long-term follow-up. Eur Spine J. 2009;18(5):654-662. doi:10.1007/s00586-009-0897-9

60. Yoo M, Kim WH, Hyun SJ, Kim KJ, Jahng TA, Kim HJ. Comparison between Two Different Cervical Interbody Fusion Cages in One Level Stand-alone ACDF: Carbon Fiber Composite Frame Cage Versus Polyetheretherketone Cage. Korean J Spine. 2014;11(3):127. doi:10.14245/kjs.2014.11.3.127

61. Shi S, Zheng S, Li XF, Yang LL, Liu ZD, Yuan W. Comparison of 2 Zero-Profile Implants in the Treatment of Single-Level Cervical Spondylotic Myelopathy: A Preliminary Clinical Study of Cervical Disc Arthroplasty versus Fusion. Grasso G, ed. PLoS ONE. 2016;11(7):e0159761. doi:10.1371/journal.pone.0159761

62. Liu Y, Hou Y, Yang L, et al. Comparison of 3 Reconstructive Techniques in the Surgical Management of Multilevel Cervical Spondylotic Myelopathy: Spine. 2012;37(23):E1450-E1458. doi:10.1097/BRS.0b013e31826c72b4

63. Shi S, Zheng S, Li XF, Yang LL, Liu ZD, Yuan W. Comparison of a Stand-Alone Anchored Spacer Versus Plate-Cage Construct in the Treatment of Two Noncontiguous Levels of Cervical Spondylosis: A Preliminary Investigation. World Neurosurgery. 2016;89:285-292. doi:10.1016/j.wneu.2016.02.009

64. Liu Y, Wang H, Li X, et al. Comparison of a zero-profile anchored spacer (ROI-C) and the polyetheretherketone (PEEK) cages with an anterior plate in anterior cervical discectomy and fusion for multilevel cervical spondylotic myelopathy. Eur Spine J. 2016;25(6):1881-1890. doi:10.1007/s00586-016-4500-x

65. Samartzis D, Shen FH, Matthews DK, Yoon ST, Goldberg EJ, An HS. Comparison of allograft to autograft in multilevelanterior cervical discectomy and fusion with rigid plate fixation. The Spine Journal. 2003;3(6):451-459. doi:10.1016/S1529-9430(03)00173-6

66. Heller JG, Sasso RC, Papadopoulos SM, et al. Comparison of BRYAN Cervical Disc Arthroplasty With Anterior Cervical Decompression and Fusion: Clinical and Radiographic Results of a Randomized, Controlled, Clinical Trial. Spine. 2009;34(2):101-107. doi:10.1097/BRS.0b013e31818ee263

67. Fisahn C, Schmidt C, Rustagi T, et al. Comparison of Chronic Dysphagia in Standalone versus Conventional Plate and Cage Fusion. World Neurosurgery. 2018;109:e382-e388. doi:10.1016/j.wneu.2017.09.188

68. Fayed I, Conte AG, Keating G, et al. Comparison of Clinical and Radiographic Outcomes After Standalone Versus Cage and Plate Constructs for Anterior Cervical Discectomy and Fusion. Int J Spine Surg. 2021;15(3):403-412. doi:10.14444/8060

69. Liu JT, Briner RP, Friedman JA. Comparison of inpatient vs. outpatient anterior cervical discectomy and fusion: a retrospective case series. BMC Surg. 2009;9(1):3. doi:10.1186/1471-2482-9-3

70. Huang C, Abudouaini H, Wang B, et al. Comparison of Patient-Reported Postoperative Dysphagia in Patients Undergoing One-Level Versus Two-Level Anterior Cervical Discectomy and Fusion with the Zero-P Implant System. Dysphagia. 2021;36(4):743-753. doi:10.1007/s00455-020-10197-w

71. Ma W, Peng Y, Zhang S, et al. Comparison of Percutaneous Endoscopic Cervical Keyhole Foraminotomy versus Microscopic Anterior Cervical Discectomy and Fusion for Single Level Unilateral Cervical Radiculopathy. IJGM. 2022;Volume 15:6897-6907. doi:10.2147/IJGM.S378837

72. Adogwa O, Elsamadicy A, Reiser E, et al. Comparison of surgical outcomes after anterior cervical discectomy and fusion: does the intra-operative use of a microscope improve surgical outcomes. J Spine Surg. 2016;2(1):25-30. doi:10.21037/jss.2016.01.04

73. Yang Y, Ma L, Liu H, et al. Comparison of the incidence of patient-reported post-operative dysphagia between ACDF with a traditional anterior plate and artificial cervical disc replacement. Clinical Neurology and Neurosurgery. 2016;148:72-78. doi:10.1016/j.clineuro.2016.07.020

74. Gowd AK, Vahidi NA, Magdycz WP, Zollinger PL, Carmouche JJ. Correlation of Voice Hoarseness and Vocal Cord Palsy: A Prospective Assessment of Recurrent Laryngeal Nerve Injury Following Anterior Cervical Discectomy and Fusion. Int J Spine Surg. 2021;15(1):12-17. doi:10.14444/8001

75. Kaufman M, Shearer J, Cabrera CI, et al. Critical analysis of the evaluation of postoperative dysphagia following an anterior cervical discectomy and fusion. American Journal of Otolaryngology. 2022;43(3):103466. doi:10.1016/j.amjoto.2022.103466

76. Türeyen K. Disc height loss after anterior cervical microdiscectomy with titanium intervertebral cage fusion. Acta Neurochirurgica. 2003;145(7):565-570. doi:10.1007/s00701-003-0050-1

77. Jang HJ, Chin DK, Kim KH, Park JY. Does Graft Position Affect Subsidence After Anterior Cervical Discectomy and Fusion? Global Spine Journal. 2022;12(4):668-676. doi:10.1177/2192568220963061

78. Pinter ZW, Monsef JB, Salmons HI, et al. Does Preoperative Bone Mineral Density Impact Fusion Success in Anterior Cervical Spine Surgery? A Prospective Cohort Study. World Neurosurgery. 2022;164:e830-e834. doi:10.1016/j.wneu.2022.05.058

79. Samartzis D, Shen FH, Lyon C, Phillips M, Goldberg EJ, An HS. Does rigid instrumentation increase the fusion rate in one-level anterior cervical discectomy and fusion? The Spine Journal. 2004;4(6):636-643. doi:10.1016/j.spinee.2004.04.010

80. Lee NJ, Vulapalli M, Park P, et al. Does screw length for primary two-level ACDF influence pseudarthrosis risk? The Spine Journal. 2020;20(11):1752-1760. doi:10.1016/j.spinee.2020.07.002

81. Lin GX, Rui G, Sharma S, Kotheeranurak V, Suen TK, Kim JS. Does the Neck Pain, Function, or Range of Motion Differ After Anterior Cervical Fusion, Cervical Disc Replacement, and Posterior Cervical Foraminotomy? World Neurosurgery. 2019;129:e485-e493. doi:10.1016/j.wneu.2019.05.188

82. Ghahreman A, Rao PJV, Ferch RD. Dynamic Plates in Anterior Cervical Fusion Surgery: Graft Settling and Cervical Alignment. Spine. 2009;34(15):1567-1571. doi:10.1097/BRS.0b013e3181a99346

83. Grasso G, Leone L, Torregrossa F. Dysphagia Prevention in Anterior Cervical Discectomy Surgery: Results from a Prospective Clinical Study. World Neurosurgery. 2019;125:e1176-e1182. doi:10.1016/j.wneu.2019.01.273

84. Wang C, Zhang Y, Yuan W. Early Clinical Outcomes and Radiographic Features After Treatment of Cervical Degenerative Disk Disease With the New Zero-Profile Implant: A 1-Year Follow-up Retrospective Study. Clinical Spine Surgery: A Spine Publication. 2016;29(2):E73-E79. doi:10.1097/BSD.0000000000000101

85. Phan K, Wang N, Kim JS, et al. Effect of Preoperative Anemia on the Outcomes of Anterior Cervical Discectomy and Fusion. Global Spine Journal. 2017;7(5):441-447. doi:10.1177/2192568217699404

86. Chou YC, Chen DC, Hsieh WA, et al. Efficacy of anterior cervical fusion: Comparison of titanium cages, polyetheretherketone (PEEK) cages and autogenous bone grafts. Journal of Clinical Neuroscience. 2008;15(11):1240-1245. doi:10.1016/j.jocn.2007.05.016

87. Song KJ, Lee KB, Song JH. Efficacy of multilevel anterior cervical discectomy and fusion versus corpectomy and fusion for multilevel cervical spondylotic myelopathy: a minimum 5-year follow-up study. Eur Spine J. 2012;21(8):1551-1557. doi:10.1007/s00586-012-2296-x

88. Jang HJ, Kim KH, Park JY, Kim KS, Cho YE, Chin DK. Endplate-specific fusion rate 1 year after surgery for two-level anterior cervical discectomy and fusion(ACDF). Acta Neurochir. 2022;164(12):3173-3180. doi:10.1007/s00701-022-05377-6

89. Kitamura K, De Dios E, Bodon G, Barany L, MacDowall A. Evaluating a paradigm shift from anterior decompression and fusion to muscle-preserving selective laminectomy: a single-center study of degenerative cervical myelopathy. Journal of Neurosurgery: Spine. 2022;37(5):740-748. doi:10.3171/2022.4.SPINE211562

90. Mok JK, Sheha ED, Samuel AM, et al. Evaluation of Current Trends in Treatment of Single-level Cervical Radiculopathy. Clinical Spine Surgery: A Spine Publication. 2019;32(5):E241-E245. doi:10.1097/BSD.0000000000000796

91. Aguilar DD, Brara HS, Rahman S, Harris J, Prentice HA, Guppy KH. Exclusion criteria for dysphagia for outpatient single-level anterior cervical discectomy and fusion using inpatient data from a spine registry. Clinical Neurology and Neurosurgery. 2019;180:28-33. doi:10.1016/j.clineuro.2019.03.008

92. Fang Z, Tian R, Sun T wei, Yadav SK, Hu W, Xie S qing. Expansion Open-door Laminoplasty With Foraminotomy Versus Anterior Cervical Discectomy and Fusion for Coexisting Multilevel Cervical Myelopathy and Unilateral Radiculopathy. Clinical Spine Surgery: A Spine Publication. 2016;29(1):E21-E27. doi:10.1097/BSD.0000000000000074

93. Trahan J, Abramova MV, Richter EO, Steck JC. Feasibility of Anterior Cervical Discectomy and Fusion as an Outpatient Procedure. World Neurosurgery. 2011;75(1):145-148. doi:10.1016/j.wneu.2010.09.015

94. Krause KL, Obayashi JT, Bridges KJ, Raslan AM, Than KD. Fivefold higher rate of pseudarthrosis with polyetheretherketone interbody device than with structural allograft used for 1-level anterior cervical discectomy and fusion: Presented at the 2018 AANS/CNS Joint Section on Disorders of the Spine and Peripheral Nerves. Journal of Neurosurgery: Spine. 2019;30(1):46-51. doi:10.3171/2018.7.SPINE18531

95. Jack MM, Lundy P, Reeves AR, Arnold PM. Four-level Anterior Cervical Discectomy and Fusions: Results Following Multilevel Cervical Fusion With a Minimum 1-Year Follow-up. Clinical Spine Surgery: A Spine Publication. 2021;34(4):E243-E247. doi:10.1097/BSD.0000000000001116

96. Ruetten S, Komp M, Merk H, Godolias G. Full-Endoscopic Cervical Posterior Foraminotomy for the Operation of Lateral Disc Herniations Using 5.9-mm Endoscopes: A Prospective, Randomized, Controlled Study. Spine. 2008;33(9):940-948. doi:10.1097/BRS.0b013e31816c8b67

97. Wewel JT, Kasliwal MK, Adogwa O, Deutsch H, O'Toole JE, Traynelis VC. Fusion rate following three- and four-level ACDF using allograft and segmental instrumentation: A radiographic study. Journal of Clinical Neuroscience. 2019;62:142-146. doi:10.1016/j.jocn.2018.11.040

98. Lubelski D, Pennington Z, Sciubba DM, Theodore N, Bydon A. Horner Syndrome After Anterior Cervical Discectomy and Fusion: Case Series and Systematic Review. World Neurosurgery. 2020;133:e68-e75. doi:10.1016/j.wneu.2019.08.101

99. Lied B, Sundseth J, Helseth E. Immediate (0–6 h), early (6–72 h) and late (>72 h) complications after anterior cervical discectomy with fusion for cervical disc degeneration; discharge six hours after operation is feasible. Acta Neurochir (Wien). 2008;150(2):111-118. doi:10.1007/s00701-007-1472-y

100. Omidi-Kashani F, Ghayem Hasankhani E, Ghandehari R. Impact of Age and Duration of Symptoms on Surgical Outcome of Single-Level Microscopic Anterior Cervical Discectomy and Fusion in the Patients with Cervical Spondylotic Radiculopathy. Neuroscience Journal. 2014;2014:1-6. doi:10.1155/2014/808596

101. Phan K, Kim JS, Lee N, Kothari P, Cho SK. Impact of Insulin Dependence on Perioperative Outcomes Following Anterior Cervical Discectomy and Fusion. Spine. 2017;42(7):456-464. doi:10.1097/BRS.0000000000001829

102. Patel MR, Jacob KC, Shah VP, et al. Impact of Surgeon Experience on Outcomes of Anterior Cervical Discectomy and Fusion. J Am Acad Orthop Surg. 2022;30(5):e537-e546. doi:10.5435/JAAOS-D-21-01080

103. Bydon M, Macki M, Kaloostian P, et al. Incidence and Prognostic Factors of C5 Palsy: A Clinical Study of 1001 Cases and Review of the Literature. Neurosurgery. 2014;74(6):595-605. doi:10.1227/NEU.0000000000000322

104. Bazaz R, Lee MJ, Yoo JU. Incidence of Dysphagia After Anterior Cervical Spine Surgery: A Prospective Study. Spine. 2002;27(22):2453-2458. doi:10.1097/00007632-200211150-00007

105. Wang JC, McDonough PW, Kanim LEA, Endow KK, Delamarter RB. Increased Fusion Rates With Cervical Plating for Three-Level Anterior Cervical Discectomy and Fusion: Spine. 2001;26(6):643-646. doi:10.1097/00007632-200103150-00015

106. Wang JC, McDonough PW, Endow KK, Delamarter RB. Increased Fusion Rates With Cervical Plating for Two-Level Anterior Cervical Discectomy and Fusion: Spine. 2000;25(1):41. doi:10.1097/00007632-200001010-00009

107. Sheperd CS, Young WF. Instrumented Outpatient Anterior Cervical Discectomy and Fusion: Is it Safe? International Surgery. 2012;97(1):86-89. doi:10.9738/CC35.1

108. Niljianskul N, Phoominaonin IS, Jaiimsin A. Intraoperative Monitoring of the Recurrent Laryngeal Nerve with Electromyography Endotracheal Tube in Anterior Cervical Discectomy and Fusion. World Neurosurgery: X. 2023;17:100141. doi:10.1016/j.wnsx.2022.100141

109. Samartzis D, Shen FH, Goldberg EJ, An HS. Is Autograft the Gold Standard in Achieving Radiographic Fusion in One-Level Anterior Cervical Discectomy and Fusion With Rigid Anterior Plate Fixation?: Spine. 2005;30(15):1756-1761. doi:10.1097/01.brs.0000172148.86756.ce

110. Kim YS, Park JY, Moon BJ, Kim SD, Lee JK. Is stand alone PEEK cage the gold standard in multilevel anterior cervical discectomy and fusion (ACDF)? Results of a minimum 1-year follow up. Journal of Clinical Neuroscience. 2018;47:341-346. doi:10.1016/j.jocn.2017.10.022

111. Sheng XQ, Meng Y, Liu H, et al. Is the fusion order of the cranial and caudal levels different in two-level anterior cervical discectomy and fusion for cervical spondylopathy? A retrospective study. J Orthop Surg Res. 2021;16(1):500. doi:10.1186/s13018-021-02657-2

112. De Leo–Vargas RA, Muñoz–Romero I, Mondragón–Soto MG, Martínez–Anda JJ. Locking Stand-Alone Cage Constructs for the Treatment of Cervical Spine Degenerative Disease. Asian Spine J. 2019;13(4):630-637. doi:10.31616/asj.2018.0234

113. Grasso G, Landi A. Long-term clinical and radiological outcomes following anterior cervical discectomy and fusion by zero-profile anchored cage. J Craniovert Jun Spine. 2018;9(2):87. doi:10.4103/jcvjs.JCVJS_36_18

114. De La Garza-Ramos R, Xu R, Ramhmdani S, et al. Long-term clinical outcomes following 3- and 4-level anterior cervical discectomy and fusion. SPI. 2016;24(6):885-891. doi:10.3171/2015.10.SPINE15795

115. Houten JK, Weinstein GR, Collins M. Long-term fate of C3-7 arthrodesis: 4-level ACDF versus cervical laminectomy and fusion. J Neurosurg Sci. 2021;65(4). doi:10.23736/S0390-5616.18.04563-0

116. Tumialán LM, Ponton RP, Gluf WM. Management of unilateral cervical radiculopathy in the military: the cost effectiveness of posterior cervical foraminotomy compared with anterior cervical discectomy and fusion. FOC. 2010;28(5):E17. doi:10.3171/2010.1.FOCUS09305

117. Dunn C, Moore J, Sahai N, et al. Minimally invasive posterior cervical foraminotomy with tubes to prevent undesired fusion: a long-term follow-up study. Journal of Neurosurgery: Spine. 2018;29(4):358-364. doi:10.3171/2018.2.SPINE171003

118. Ng MK, Kobryn A, Baidya J, et al. Multi-Level Posterior Cervical Foraminotomy Associated With Increased Post-operative Infection Rates and Overall Re-Operation Relative to Anterior Cervical Discectomy With Fusion or Cervical Disc Arthroplasty. Global Spine Journal. Published online September 2, 2022:219256822211245. doi:10.1177/21925682221124530

119. Lu DC, Tumialán LM, Chou D. Multilevel anterior cervical discectomy and fusion with and without rhBMP-2: a comparison of dysphagia rates and outcomes in 150 patients: Clinical article. SPI. 2013;18(1):43-49. doi:10.3171/2012.10.SPINE10231

120. Neifert SN, Gal JS, Chapman EK, Caridi JM. Nonhome Discharge as an Independent Risk Factor for Adverse Events and Readmission in Patients Undergoing Anterior Cervical Discectomy and Fusion. Clinical Spine Surgery: A Spine Publication. 2020;33(10):E454-E459. doi:10.1097/BSD.0000000000000961

121. Yagi K, Nakagawa H, Okazaki T, et al. Noninfectious prevertebral soft-tissue inflammation and hematoma eliciting swelling after anterior cervical discectomy and fusion. SPI. 2017;26(4):459-465. doi:10.3171/2016.9.SPINE16520

122. Bolesta MJ, Rechtine GR, Chrin AM. One- and two-level anterior cervical discectomy and fusion: the effect of plate fixation. The Spine Journal. 2002;2(3):197-203. doi:10.1016/S1529-9430(02)00186-9

123. Lambrechts MJ, D'Antonio ND, Heard JC, et al. Opioid Use Increases the Rate of Pseudarthrosis and Revision Surgery in Patients Undergoing Anterior Cervical Discectomy and Fusion. Global Spine Journal. Published online August 12, 2022:219256822211191. doi:10.1177/21925682221119132

124. Hwang SL, Hwang YF, Lieu AS, et al. Outcome Analyses of Interbody Titanium Cage Fusion Used in the Anterior Discectomy for Cervical Degenerative Disc Disease. Journal of Spinal Disorders & Techniques. 2005;18(4):326-331. doi:10.1097/01.bsd.0000164198.30725.2d

125. Cauthen JC, Kinard RE, Vogler JB, et al. Outcome Analysis of Noninstrumented Anterior Cervical Discectomy and Interbody Fusion in 348 Patients: Spine. 1998;23(2):188-192. doi:10.1097/00007632-199801150-00008

126. Yamagata T, Takami T, Uda T, et al. Outcomes of contemporary use of rectangular titanium stand-alone cages in anterior cervical discectomy and fusion: Cage subsidence and cervical alignment. Journal of Clinical Neuroscience. 2012;19(12):1673-1678. doi:10.1016/j.jocn.2011.11.043

127. Tracey RW, Kang DG, Cody JP, Wagner SC, Rosner MK, Lehman RA. Outcomes of single-level cervical disc arthroplasty versus anterior cervical discectomy and fusion. Journal of Clinical Neuroscience. 2014;21(11):1905-1908. doi:10.1016/j.jocn.2014.05.007

128. Guo Q, Bi X, Ni B, et al. Outcomes of three anterior decompression and fusion techniques in the treatment of three-level cervical spondylosis. Eur Spine J. 2011;20(9):1539-1544. doi:10.1007/s00586-011-1735-4

129. Lied B, Rønning PA, Halvorsen CM, Ekseth K, Helseth E. Outpatient anterior cervical discectomy and fusion for cervical disk disease: a prospective consecutive series of 96 patients. Acta Neurol Scand. 2013;127(1):31-37. doi:10.1111/j.1600-0404.2012.01674.x

130. Rossi V, Asher A, Peters D, et al. Outpatient anterior cervical discectomy and fusion in the ambulatory surgery center setting: safety assessment for the Medicare population. Journal of Neurosurgery: Spine. 2020;32(3):360-365. doi:10.3171/2019.7.SPINE19480

131. Joseffer SS, Shin P, Wohns RNW. Outpatient Anterior Cervical Discectomy and Fusion: Indications and Clinical Experience in a Consecutive Series of 390 Patients: Neurosurgery Quarterly. 2010;20(2):107-110. doi:10.1097/WNQ.0b013e3181dcb595

132. Klingler JH, Krüger MT, Sircar R, et al. PEEK Cages versus PMMA Spacers in Anterior Cervical Discectomy: Comparison of Fusion, Subsidence, Sagittal Alignment, and Clinical Outcome with a Minimum 1-Year Follow-Up. The Scientific World Journal. 2014;2014:1-11. doi:10.1155/2014/398396

133. Ahn Y, Keum HJ, Shin SH. Percutaneous Endoscopic Cervical Discectomy versus Anterior Cervical Discectomy and Fusion: A Comparative Cohort Study with a Five-Year Follow-Up. JCM. 2020;9(2):371. doi:10.3390/jcm9020371

134. Yue WM, Brodner W, Highland TR. Persistent swallowing and voice problems after anterior cervical discectomy and fusion with allograft and plating: a 5- to 11-year follow-up study. Eur Spine J. 2005;14(7):677-682. doi:10.1007/s00586-004-0849-3

135. Song KJ, Taghavi CE, Hsu MS, Lee KB, Kim GH, Song JH. Plate augmentation in anterior cervical discectomy and fusion with cage for degenerative cervical spinal disorders. Eur Spine J. 2010;19(10):1677-1683. doi:10.1007/s00586-010-1283-3

136. Landriel FA, Hem S, Goldschmidt E, Ajler P, Vecchi E, Carrizo A. Polyetheretherketone Interbody Cages Versus Autogenous Iliac Crest Bone Grafts With Anterior Fixation for Cervical Disc Disease. Journal of Spinal Disorders & Techniques. 2013;26(2):61-67. doi:10.1097/BSD.0b013e3182323274

137. Korinth MC, Krüger A, Oertel MF, Gilsbach JM. Posterior Foraminotomy or Anterior Discectomy With Polymethyl Methacrylate Interbody Stabilization for Cervical Soft Disc Disease: Results in 292 Patients With Monoradiculopathy: Spine. 2006;31(11):1207-1214. doi:10.1097/01.brs.0000217604.02663.59

138. Chughtai M, Sultan AA, Padilla J, et al. Postoperative stroke after anterior cervical discectomy and fusion in patients with carotid artery stenosis: a statewide database analysis. The Spine Journal. 2019;19(4):597-601. doi:10.1016/j.spinee.2018.09.011

139. Nguyen S, Sherrod BA, Paziuk TM, et al. Predictors of Dysphagia After Anterior Cervical Discectomy and Fusion: A Prospective Multicenter Study. Spine. 2022;47(12):859-864. doi:10.1097/BRS.0000000000004279

140. Lovecchio F, Hsu WK, Smith TR, Cybulski G, Kim B, Kim JYS. Predictors of Thirty-Day Readmission After Anterior Cervical Fusion: Spine. 2014;39(2):127-133. doi:10.1097/BRS.0000000000000051

141. Cao Y, Xu C, Sun B, et al. Preoperative Cervical Cobb Angle Is a Risk Factor for Postoperative Axial Neck Pain after Anterior Cervical Discectomy and Fusion with Zero-Profile Interbody. Orthopaedic Surgery. 2022;14(12):3225-3232. doi:10.1111/os.13552

142. Wang KY, Suresh KV, Mo K, Harris AB, Marrache M, Kebaish KM. Preoperative Hyponatremia Is an Independent Risk Factor for Prolonged Hospital Stay After Anterior Cervical Discectomy and Fusion. World Neurosurgery. 2022;161:e18-e24. doi:10.1016/j.wneu.2021.10.125

143. Fu MC, Buerba RA, Grauer JN. Preoperative Nutritional Status as an Adjunct Predictor of Major Postoperative Complications Following Anterior Cervical Discectomy and Fusion. Clinical Spine Surgery: A Spine Publication. 2016;29(4):167-172. doi:10.1097/BSD.0000000000000181

144. Choi SH, Cho JH, Hwang CJ, Lee CS, Gwak HW, Lee DH. Preoperative Radiographic Parameters to Predict a Higher Pseudarthrosis Rate After Anterior Cervical Discectomy and Fusion. Spine. 2017;42(23):1772-1778. doi:10.1097/BRS.0000000000002219

145. Kamalapathy PN, Wang KY, Puvanesarajah V, Raad M, Hassanzadeh H. Presence and Severity of Mental Illness Is Associated With Increased Risk of Postoperative Emergency Visits, Readmission, and Reoperation Following Outpatient ACDF: A National Database Analysis. Global Spine Journal. 2023;13(5):1267-1272. doi:10.1177/21925682211026913

146. Wong M, Williams N, Kacker A. Primary and Revision Anterior Cervical Discectomy and Fusion: A Study of Otolaryngologic Outcomes in a Large Cohort. Spine. 2021;46(24):1677-1682. doi:10.1097/BRS.0000000000004089

147. Zigler JE, Delamarter R, Murrey D, Spivak J, Janssen M. ProDisc-C and Anterior Cervical Discectomy and Fusion as Surgical Treatment for Single-Level Cervical Symptomatic Degenerative Disc Disease: Five-Year Results of a Food and Drug Administration Study. Spine. 2013;38(3):203-209. doi:10.1097/BRS.0b013e318278eb38

148. Coric D, Kim PK, Clemente JD, Boltes MO, Nussbaum M, James S. Prospective randomized study of cervical arthroplasty and anterior cervical discectomy and fusion with long-term follow-up: results in 74 patients from a single site. SPI. 2013;18(1):36-42. doi:10.3171/2012.9.SPINE12555

149. Coric D, Cassis J, Carew JD, Boltes MO. Prospective study of cervical arthroplasty in 98 patients involved in 1 of 3 separate investigational device exemption studies from a single investigational site with a minimum 2-year follow-up. SPI. 2010;13(6):715-721. doi:10.3171/2010.5.SPINE09852

150. Coric D, Nunley PD, Guyer RD, et al. Prospective, randomized, multicenter study of cervical arthroplasty: 269 patients from the Kineflex|C artificial disc investigational device exemption study with a minimum 2-year follow-up: Clinical article. SPI. 2011;15(4):348-358. doi:10.3171/2011.5.SPINE10769

151. Ikenaga M, Shikata J, Tanaka C. Radiculopathy of C-5 after anterior decompression for cervical myelopathy. Journal of Neurosurgery: Spine. 2005;3(3):210-217. doi:10.3171/spi.2005.3.3.0210

152. Sinensky AM, Kaye ID, Li WT, et al. Radiographic Measures of Spinal Alignment Are Not Predictive of the Development of C5 Palsy Following Anterior Cervical Discectomy and Fusion Surgery. Int J Spine Surg. 2021;15(2):213-218. doi:10.14444/8029

153. Ahn SS, So WS, Ku MG, Kim SH, Kim DW, Lee BH. Radiologic Findings and Risk Factors of Adjacent Segment Degeneration after Anterior Cervical Discectomy and Fusion : A Retrospective Matched Cohort Study with 3-Year Follow-Up Using MRI. J Korean Neurosurg Soc. 2016;59(2):129. doi:10.3340/jkns.2016.59.2.129

154. Oni P, Schultheiß R, Scheufler KM, Roberg J, Harati A. Radiological and Clinical Outcome after Multilevel Anterior Cervical Discectomy and/or Corpectomy and Fixation. JCM. 2018;7(12):469. doi:10.3390/jcm7120469

155. Chang HK, Huang WC, Tu TH, et al. Radiological and clinical outcomes of 3-level cervical disc arthroplasty. Journal of Neurosurgery: Spine. 2020;32(2):174-181. doi:10.3171/2019.8.SPINE19545

156. Riederman BD, Butler BA, Lawton CD, Rosenthal BD, Balderama ES, Bernstein AJ. Recombinant human bone morphogenetic protein-2 versus iliac crest bone graft in anterior cervical discectomy and fusion: Dysphagia and dysphonia rates in the early postoperative period with review of the literature. Journal of Clinical Neuroscience. 2017;44:180-183. doi:10.1016/j.jocn.2017.06.034

157. Huschbeck A, Knoop M, Gahleitner A, et al. Recurrent Laryngeal Nerve Palsy after Anterior Cervical Discectomy and Fusion – Prevalence and Risk Factors. J Neurol Surg A Cent Eur Neurosurg. 2020;81(06):508-512. doi:10.1055/s-0040-1710351

158. Staartjes VE, De Wispelaere MP, Schröder ML. Recurrent Laryngeal Nerve Palsy Is More Frequent After Secondary than After Primary Anterior Cervical Discectomy and Fusion: Insights from a Registry of 525 Patients. World Neurosurgery. 2018;116:e1047-e1053. doi:10.1016/j.wneu.2018.05.162

159. Nayak R, Razzouk J, Ramos O, et al. Reoperation and Perioperative Complications After Surgical Treatment of Cervical Radiculopathy: A Comparison Between Three Procedures. Spine. 2023;48(4):261-269. doi:10.1097/BRS.0000000000004506

160. Wei Z, Zhang Y, Yang S, et al. Retrospective Analysis of Sagittal Balance Parameters and Clinical Efficacy After Short-Segment Anterior Cervical Spine Surgery with Different Fusion Devices. IJGM. 2022;Volume 15:3237-3246. doi:10.2147/IJGM.S340877

161. Helseth Ø, Lied B, Heskestad B, Ekseth K, Helseth E. Retrospective single-centre series of 1300 consecutive cases of outpatient cervical spine surgery: complications, hospital readmissions, and reoperations. British Journal of Neurosurgery. 2019;33(6):613-619. doi:10.1080/02688697.2019.1675587

162. Mullins J, Pojskić M, Boop FA, Arnautović KI. Retrospective single-surgeon study of 1123 consecutive cases of anterior cervical discectomy and fusion: a comparison of clinical outcome parameters, complication rates, and costs between outpatient and inpatient surgery groups, with a literature review. Journal of Neurosurgery: Spine. 2018;28(6):630-641. doi:10.3171/2017.10.SPINE17938

163. Wang LF, Dong Z, Miao DC, Shen Y, Wang F. Risk factor analysis of axial symptoms after single-segment anterior cervical discectomy and fusion: A retrospective study of 113 patients. J Int Med Res. 2019;47(12):6100-6108. doi:10.1177/0300060519884828

164. Shi S, Li XF, Zhao QT, Yang LL, Liu ZD, Yuan W. Risk Factors for Dysphagia After Single-Level Anterior Cervical Decompression with Arthroplasty or Fusion: A Prospective Study Comparing 2 Zero-Profile Implants. World Neurosurgery. 2016;95:148-155. doi:10.1016/j.wneu.2016.07.100

165. Narain AS, Hijji FY, Haws BE, et al. Risk Factors for Medical and Surgical Complications after 1–2-Level Anterior Cervical Discectomy and Fusion Procedures. Int J Spine Surg. 2020;14(3):286-293. doi:10.14444/7038

166. Lee YS, Kim YB, Park SW. Risk Factors for Postoperative Subsidence of Single-Level Anterior Cervical Discectomy and Fusion: The Significance of the Preoperative Cervical Alignment. Spine. 2014;39(16):1280-1287. doi:10.1097/BRS.0000000000000400

167. Kao TH, Wu CH, Chou YC, Chen HT, Chen WH, Tsou HK. Risk factors for subsidence in anterior cervical fusion with stand-alone polyetheretherketone (PEEK) cages: a review of 82 cases and 182 levels. Arch Orthop Trauma Surg. 2014;134(10):1343-1351. doi:10.1007/s00402-014-2047-z

168. Ren B, Gao W, An J, Wu M, Shen Y. Risk factors of cage nonunion after anterior cervical discectomy and fusion. Medicine. 2020;99(12):e19550. doi:10.1097/MD.0000000000019550

169. Zhang B, Li S, Miao D, Zhao C, Wang L. Risk Factors of Cage Subsidence in Patients with Ossification of Posterior Longitudinal Ligament (OPLL) After Anterior Cervical Discectomy and Fusion. Med Sci Monit. 2018;24:4753-4759. doi:10.12659/MSM.910964

170. Alsoof D, Perry J, Yang DS, et al. Risk of Dysphagia and Dysphonia in Patients With Prior Thyroidectomy Undergoing Anterior Cervical Discectomy and Fusion. Global Spine Journal. Published online July 14, 2022:219256822211110. doi:10.1177/21925682221111095

171. Tally WC, Tarabadkar S, Kovalenko BV. Safety and feasibility of outpatient ACDF in an ambulatory setting: A retrospective chart review. The International Journal of Spine Surgery. 2013;7(1):e84-e87. doi:10.1016/j.ijsp.2013.06.001

172. Feng YT, Hwang SL, Lin CL, Lee IC, Lee KT. Safety and resource utilization of anterior cervical discectomy and fusion. The Kaohsiung Journal of Medical Sciences. 2012;28(9):495-499. doi:10.1016/j.kjms.2012.04.007

173. Vaishnav A, Hill P, McAnany S, Gang CH, Qureshi S. Safety of 2-level Anterior Cervical Discectomy and Fusion (ACDF) Performed in an Ambulatory Surgery Setting With Same-day Discharge. Clinical Spine Surgery: A Spine Publication. 2019;32(3):E153-E159. doi:10.1097/BSD.0000000000000753

174. Garringer SM, Sasso RC. Safety of Anterior Cervical Discectomy and Fusion Performed as Outpatient Surgery. Journal of Spinal Disorders & Techniques. 2010;23(7):439-443. doi:10.1097/BSD.0b013e3181bd0419

175. Yang JJ, Park S, Kim HJ, Yoon JY. Segmental Height Decrease Adversely Affects Foraminal Height and Cervical Lordosis, But Not Clinical Outcome After Anterior Cervical Discectomy and Fusion Using Allografts. World Neurosurgery. 2021;154:e555-e565. doi:10.1016/j.wneu.2021.07.088

176. Mansfield HE, Canar WJ, Gerard CS, O'Toole JE. Single-level anterior cervical discectomy and fusion versus minimally invasive posterior cervical foraminotomy for patients with cervical radiculopathy: a cost analysis. FOC. 2014;37(5):E9. doi:10.3171/2014.8.FOCUS14373

177. Lambrechts MJ, Issa TZ, Toci GR, et al. Soft Cervical Orthosis Use Does Not Improve Fusion Rates After One-Level and Two-Level Anterior Cervical Discectomy and Fusion. World Neurosurgery. 2022;167:e1461-e1467. doi:10.1016/j.wneu.2022.09.095

178. Labaran LA, Harris AB, Puvanesarajah V, et al. Solid Organ Transplant Is Associated With Increased Morbidity and Mortality in Patients Undergoing One or Two-level Anterior Cervical Decompression and Fusion. Spine. 2020;45(3):158-162. doi:10.1097/BRS.0000000000003230

179. Kast E, Derakhshani S, Bothmann M, Oberle J. Subsidence after anterior cervical inter-body fusion. A randomized prospective clinical trial. Neurosurg Rev. 2009;32(2):207-214. doi:10.1007/s10143-008-0168-y

180. Minhas SV, Chow I, Patel AA, Kim JYS. Surgeon Specialty Differences in Single-Level Anterior Cervical Discectomy and Fusion: Spine. 2014;39(20):1648-1655. doi:10.1097/BRS.0000000000000499

181. Nanda A, Sharma M, Sonig A, Ambekar S, Bollam P. Surgical Complications of Anterior Cervical Diskectomy and Fusion for Cervical Degenerative Disk Disease: A Single Surgeon's Experience of 1576 Patients. World Neurosurgery. 2014;82(6):1380-1387. doi:10.1016/j.wneu.2013.09.022

182. Padhye K, Shultz P, Alcala C, et al. Surgical Treatment of Single Level Cervical Radiculopathy: A Comparison of Anterior Cervical Decompression and Fusion (ACDF) Versus Cervical Disk Arthroplasty (CDA) Versus Posterior Cervical Foraminotomy (PCF). Clinical Spine Surgery: A Spine Publication. 2022;35(4):149-154. doi:10.1097/BSD.0000000000001316

183. Hao Q, Ding Q, Yang H, et al. Symptomatic postoperative spinal epidural hematoma preferentially occurs after anterior cervical discectomy and fusion versus anterior cervical corpectomy and fusion: a retrospective study. Ann Palliat Med. 2022;11(6):2025-2032. doi:10.21037/apm-22-488

184. Wang Z, Jiang W, Li X, et al. The application of zero-profile anchored spacer in anterior cervical discectomy and fusion. Eur Spine J. 2015;24(1):148-154. doi:10.1007/s00586-014-3628-9

185. Elsamadicy A, Adogwa O, Reiser E, Fatemi P, Cheng J, Bagley C. The Effect of Patient Race on Extent of Functional Improvement After Cervical Spine Surgery: SPINE. 2016;41(9):822-826. doi:10.1097/BRS.0000000000001346

186. Ahn SS, Paik HK, Chin DK, Kim SH, Kim DW, Ku MG. The Fate of Adjacent Segments After Anterior Cervical Discectomy and Fusion: The Influence of an Anterior Plate System. World Neurosurgery. 2016;89:42-50. doi:10.1016/j.wneu.2016.01.013

187. Shuman WH, Neifert SN, Gal JS, et al. The Impact of Diabetes on Outcomes and Health Care Costs Following Anterior Cervical Discectomy and Fusion. Global Spine Journal. 2022;12(5):780-786. doi:10.1177/2192568220964053

188. Yan Y, Wu C, Huang C, et al. The Presence of Thyroid Cartilage at the Surgical Level Reduces Early Dysphagia after Single-Level Anterior Cervical Surgery: A Retrospective Study. Journal of Investigative Surgery. 2020;33(4):365-374. doi:10.1080/08941939.2018.1520939

189. Bivona LJ, Camacho JE, Usmani F, et al. The Prevalence of Bacterial Infection in Patients Undergoing Elective ACDF for Degenerative Cervical Spine Conditions: A Prospective Cohort Study With Contaminant Control. Global Spine Journal. 2021;11(1):13-20. doi:10.1177/2192568219888179

190. Bucci M, Oh D, Cowan RS, et al. The ROI-C zero-profile anchored spacer for anterior cervical discectomy and fusion: biomechanical profile and clinical outcomes. MDER. 2017;Volume 10:61-69. doi:10.2147/MDER.S127133

191. Tumialán LM, Pan J, Rodts GE, Mummaneni PV. The safety and efficacy of anterior cervical discectomy and fusion with polyetheretherketone spacer and recombinant human bone morphogenetic protein–2: a review of 200 patients. SPI. 2008;8(6):529-535. doi:10.3171/SPI/2008/8/6/529

192. Villavicencio AT, Pushchak E, Burneikiene S, Thramann JJ. The safety of instrumented outpatient anterior cervical discectomy and fusion. The Spine Journal. 2007;7(2):148-153. doi:10.1016/j.spinee.2006.04.009

193. Martin CT, Pugely AJ, Gao Y, Mendoza-Lattes S. Thirty-Day Morbidity After Single-Level Anterior Cervical Discectomy and Fusion: Identification of Risk Factors and Emphasis on the Safety of Outpatient Procedures. The Journal of Bone and Joint Surgery. 2014;96(15):1288-1294. doi:10.2106/JBJS.M.00767

194. Cho DY, Lee WY, Sheu PC. Treatment of multilevel cervical fusion with cages. Surgical Neurology. 2004;62(5):378-385. doi:10.1016/j.surneu.2004.01.021

195. An SB, Lee JJ, Kim TW, et al. Upper Cervical Surgery, Increased Signal Intensity of the Spinal Cord, and Hypertension as Risk Factors for Dyspnea After Multilevel Anterior Cervical Discectomy and Fusion. Spine. 2020;45(7):E379-E386. doi:10.1097/BRS.0000000000003329

196. Lee DH, Cho JH, Hwang CJ, et al. What Is the Fate of Pseudarthrosis Detected 1 Year After Anterior Cervical Discectomy and Fusion? Spine. 2018;43(1):E23-E28. doi:10.1097/BRS.0000000000002077

197. Rihn JA, Kane J, Albert TJ, Vaccaro AR, Hilibrand AS. What Is the Incidence and Severity of Dysphagia After Anterior Cervical Surgery? Clinical Orthopaedics & Related Research. 2011;469(3):658-665. doi:10.1007/s11999-010-1731-8

198. Lambrechts MJ, D'Antonio ND, Karamian BA, et al. What is the role of dynamic cervical spine radiographs in predicting pseudarthrosis revision following anterior cervical discectomy and fusion? The Spine Journal. 2022;22(10):1610-1621. doi:10.1016/j.spinee.2022.04.020

199. Barbagallo GMV, Romano D, Certo F, Milone P, Albanese V. Zero-P: a new zero-profile cage-plate device for single and multilevel ACDF. A single Institution series with four years maximum follow-up and review of the literature on zero-profile devices. Eur Spine J. 2013;22(S6):868-878. doi:10.1007/s00586-013-3005-0

200. Hofstetter CP, Kesavabhotla K, Boockvar JA. Zero-profile Anchored Spacer Reduces Rate of Dysphagia Compared With ACDF With Anterior Plating. Journal of Spinal Disorders & Techniques. 2015;28(5):E284-E290. doi:10.1097/BSD.0b013e31828873ed

201. Niljianskul N. ZERO-PROFILE DEVICE IMPLANTATION IN ANTERIOR CERVICAL DISCECTOMY AND FUSION: A SINGLE INSTITUTE EXPERIENCE. J Southeast Asian Med Res. 2022;6:e0104. doi:10.55374/jseamed.v6i0.104

202. Wang Z, Zhu R, Yang H, et al. Zero-profile implant (Zero-p) versus plate cage benezech implant (PCB) in the treatment of single-level cervical spondylotic myelopathy. BMC Musculoskelet Disord. 2015;16(1):290. doi:10.1186/s12891-015-0746-4

203. Iampreechakul P, Srisawat C, Tirakotai W. Stand-alone cervical polyetheretherketone (PEEK) cage (cervios) for single to two-level degenerative disc disease. J Med Assoc Thai. 2011;94(2):185-192.

204. Chen JF, Wu CT, Lee ST. The use of a hollow polymethylmethacrylate cervical spacer with plating in the treatment of single level cervical disc disease. Chang Gung Med J. 2009;32(4):447-454.

205. Opsenak R, Kolarovszki B, Benco M, et al. Dysphagia after anterior cervical discectomy and interbody fusion - prospective study with 1-year follow-up. Rozhl Chir. 2019;98(3):115-120.

206. Wang JC, McDonough PW, Endow K, Kanim LE, Delamarter RB. The effect of cervical plating on single-level anterior cervical discectomy and fusion. J Spinal Disord. 1999;12(6):467-471.

207. Xiong X, Liu JM, Chen WW, et al. Outcomes of different zero-profile spacers in the treatment of two-level cervical degenerative disk disease. Eur Spine J. 2023;32(7):2448-2458. doi:10.1007/s00586-023-07756-8

208. Aguirre AO, Soliman MAR, Minissale NJ, et al. Outcomes of 2-Level Versus 3- or 4-Level Anterior Cervical Discectomy and Fusion Using a Biomimetic Surface Titanium Cage: Multicenter Experience. World Neurosurgery. 2023;177:e453-e459. doi:10.1016/j.wneu.2023.06.074

209. Baig AA, Aguirre AO, Soliman MAR, et al. Standalone versus Anterior Cervical Plating for One-To-Two Level Anterior Cervical Discectomy and Fusion: A Propensity Score-Matched Comparative Study. World Neurosurgery. 2023;177:e673-e679. doi:10.1016/j.wneu.2023.06.121

210. Zhuo W, Huang F, Zhu W, Liu F, Liu J, Jin D. Analysis of risk factors for non-fusion of bone graft in anterior cervical discectomy and fusion: A clinical retrospective study. Journal of Orthopaedic Science. Published online July 2023:S0949265823001902. doi:10.1016/j.jos.2023.07.002

211. Chanbour H, Bendfeldt GA, Johnson GW, et al. Longer Screws Decrease the Risk of Radiographic Pseudarthrosis Following Elective Anterior Cervical Discectomy and Fusion. Global Spine Journal. Published online November 11, 2023:21925682231214361. doi:10.1177/21925682231214361

212. Paziuk T, Mazmudar AS, Issa TZ, et al. Does operative level impact dysphagia severity following anterior cervical discectomy and fusion? a multicenter prospective analysis. Spine. Published online February 19, 2024. doi:10.1097/BRS.0000000000004965

213. Guo J, Jin W, Shi Y, et al. Is the Zero-P Spacer Suitable for 3-Level Anterior Cervical Discectomy and Fusion Surgery in Terms of Sagittal Alignment Reconstruction: A Comparison Study with Traditional Plate and Cage System. Brain Sciences. 2022;12(11):1583. doi:10.3390/brainsci12111583

214. Eseonu K, Laurent E, Bishi H, Raja H, Ravi K, Dannawi Z. A Retrospective Comparative Study of Long-Term Outcomes Following Cervical Total Disc Replacement Versus Anterior Cervical Discectomy and Fusion. Cureus. Published online December 11, 2022. doi:10.7759/cureus.32399

215. Tang L, Liu X, Lu Y, Liu Y, Yu J, Zhao J. Clinical and imaging outcomes of self-locking stand-alone cages and anterior cage-with-plate in three-level anterior cervical discectomy and fusion: a retrospective comparative study. J Orthop Surg Res. 2023;18(1):276. doi:10.1186/s13018-023-03726-4

216. D’Antonio N, Lambrechts MJ, Heard J, et al. Effect of Interbody Composition on the Development of Pseudarthrosis Following Anterior Cervical Discectomy and Fusion. Asian Spine J. 2023;17(3):518-528. doi:10.31616/asj.2022.0258

217. Wang H, Yu H, Zhang N, Xiang L. Incidence, Risk Factors, and Management of Postoperative Hematoma Following Anterior Cervical Decompression and Fusion for Degenerative Cervical Diseases. Neurospine. 2023;20(2):525-535. doi:10.14245/ns.2245066.533

218. Porche K, Vaziri S, Stein A, et al. The effect of myelopathic symptoms on hospital costs, length of stay, and discharge location in anterior cervical discectomy and fusion. Neurosurgical Focus. 2023;55(3):E8. doi:10.3171/2023.6.FOCUS23288

219. White MD, Farber SH, Pacult MA, et al. Pseudarthrosis after four-level anterior cervical discectomy and fusion without posterior fixation. Neurosurgical Focus. 2023;55(3):E4. doi:10.3171/2023.6.FOCUS23303

220. Opsenak R, Snopko P, Sutovsky J, et al. Incidence and severity of dysphagia after anterior cervical discectomy and fusion with zero-profile spacer: prospective study with 3-years follow-up. BLL. 2023;124(12):879-885. doi:10.4149/BLL_2023_136

221. Zhao HH, Xu HW, Wang SJ, Hu T, Wu DS. Anterior cervical discectomy and fusion with a zero-profile VA spacer device: a clinical and radiological study with two-year follow-up. J Orthop Surg Res. 2024;19(1):51. doi:10.1186/s13018-024-04539-9

222. Tang L, Chen Y, Wang F, et al. Safety and efficacy of day anterior cervical discectomy and fusion procedure for degenerative cervical spondylosis: a retrospective analysis. BMC Musculoskelet Disord. 2024;25(1):223. doi:10.1186/s12891-024-07356-7
